# Conservative versus early surgical treatment in the management of pyogenic spondylodiscitis: a systematic review and meta-analysis

**DOI:** 10.1038/s41598-023-41381-1

**Published:** 2023-09-20

**Authors:** Santhosh G. Thavarajasingam, Kalyan V. Vemulapalli, Sajeenth Vishnu K., Hariharan Subbiah Ponniah, Alexander Sanchez-Maroto Vogel, Robert Vardanyan, Jonathan Neuhoff, Andreas Kramer, Ehab Shiban, Florian Ringel, Andreas K. Demetriades, Benjamin M. Davies

**Affiliations:** 1 Faculty of Medicine, Imperial College London, Reynolds Building, St Dunstan’s Road, London, W6 8RP UK; 2 Department of Academic Neurosurgery, Addenbrooke’s Hospital, Cambridge University Hospital NHS Healthcare Trust, Cambridge, UK; 3 Imperial Brain and Spine Initiative, Imperial College London, London, UK; 4 Faculty of Medicine, Goethe-Universität Frankfurt, Frankfurt, Germany; 5 Center for Spinal Surgery and Neurotraumatology, Berufsgenossenschaftliche Unfallklinik Frankfurt am Main, Frankfurt, Germany; 6 Department of Neurosurgery, Universitätsmedizin Mainz, Mainz, Germany; 7 Department of Neurosurgery, Universitätsklinikum Augsburg, Augsburg, Germany; 8 Edinburgh Spinal Surgery Outcome Studies Group, Department of Neurosurgery, Division of Clinical Neurosciences, NHS Lothian, Edinburgh University Hospitals, Edinburgh, UK; 9 Spondylodiscitis Study Group, EANS Spine Section, Hamburg, Germany; 10 Department of Neurosurgery, University Medical Center Mainz, Mainz, Germany

**Keywords:** Neurology, Neurological disorders, Spinal cord diseases, Medical research, Outcomes research, Infectious diseases, Bacterial infection

## Abstract

Spondylodiscitis is the commonest spine infection, and pyogenic spondylodiscitis is the most common subtype. Whilst antibiotic therapy is the mainstay of treatment, some advocate that early surgery can improve mortality, relapse rates, and length of stay. Given that the condition carries a high mortality rate of up to 20%, the most effective treatment must be identified. We aimed to compare the mortality, relapse rate, and length of hospital stay of conservative versus early surgical treatment of pyogenic spondylodiscitis. All major databases were searched for original studies, which were evaluated using a qualitative synthesis, meta-analyses, influence, and regression analyses. The meta-analysis, with an overall pooled sample size of 10,954 patients from 21 studies, found that the pooled mortality among the early surgery patient subgroup was 8% versus 13% for patients treated conservatively. The mean proportion of relapse/failure among the early surgery subgroup was 15% versus 21% for the conservative treatment subgroup. Further, it concluded that early surgical treatment, when compared to conservative management, is associated with a 40% and 39% risk reduction in relapse/failure rate and mortality rate, respectively, and a 7.75 days per patient reduction in length of hospital stay (p < 0.01). The meta-analysis demonstrated that early surgical intervention consistently significantly outperforms conservative management in relapse/failure and mortality rates, and length of stay, in patients with pyogenic spondylodiscitis.

## Introduction

The incidence of spondylodiscitis, the commonest infection of the vertebral bodies and intervertebral discs, has recently been reported to be as high as 4.4 per 100,000 per year in the Western world, carrying a high mortality rate of up to 20%^1–7^. Chronic infection and subtherapeutic treatment can lead to persistent spinal deformity, neurological deficits, permanent reduction in quality of life, residual pain needing long-term analgesia, and mortality in otherwise healthy individuals^2,3,6,7^. Once predominantly caused by granulomatous spondylodiscitis, pyogenic spondylodiscitis now prevails due to improved diagnostics and a more susceptible population^6,8–10^.

At present, conservative treatment, the most commonly used treatment option, consists of long-term antibiotics, the duration and specifics of which are highly debated^11^. Indications for surgery include failure of conservative management, mechanical instability, and compression of neurological structures. Early surgery has been hypothesised to accelerate clearance and prevent deformity, but its role remains controversial. Given the significant implications of spondylodiscitis, and the increasing incidence, defining the role of early surgery is critical.

Present literature often cites age and co-morbidities as being vital in deciding optimal treatment strategies^12,13^. Lesion subtypes may be vital too; patients with spinal epidural abscesses (SEAs) may be preferentially managed with surgical debridement and decompression^14^. However, there is a clear source of heterogeneity in the findings of current studies. For example, the seminal review by Rutges et al. found that early antibiotics were important in improving outcomes and found an anterior surgical approach to be beneficial^15^. Nonetheless, they were unable to recommend early surgical or conservative management over the other, due to data heterogeneity. However, the authors did not attempt to explore or mitigate this.

To facilitate decision-making in the management of spondylodiscitis, a robust quantitative and qualitative synthesis is required. This study therefore aimed to define the role of early surgery in spondylodiscitis, in comparison to conservative management.

## Methods

### Search strategy and selection criteria

**Figure 1:**
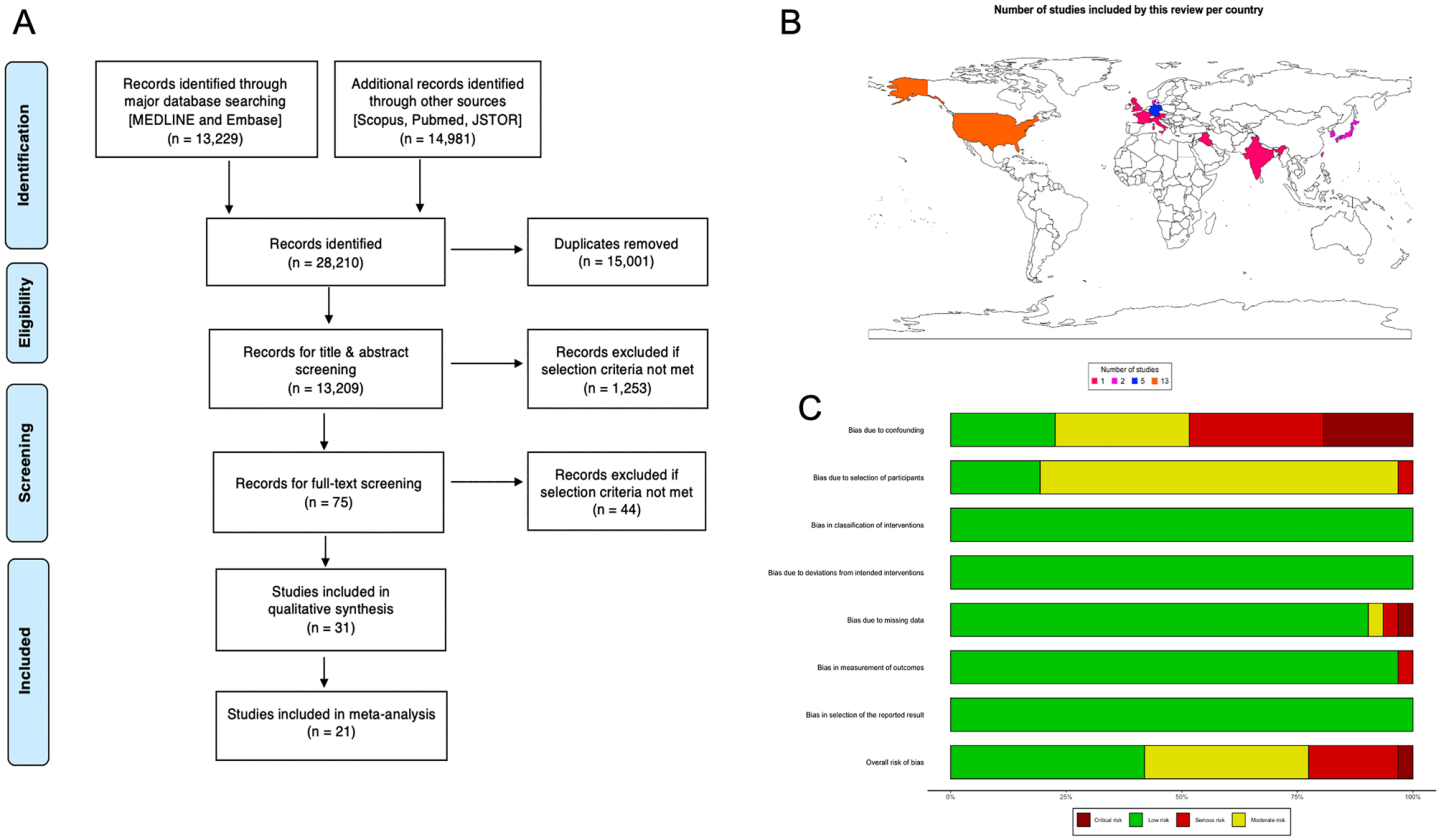
(**A**) The preferred Reporting Items for Systematic Reviews and Meta-Analyses (PRISMA) flowchart outlining the study selection process is shown. (**B**) A world map indicated the origin of publications included in this study (n = 31)^13,14,23–52^. The countries are coloured according to whether n = 1, 2, 5 or 13 studies from these countries have been included in this systematic review. The legend at the bottom indicates the colour coding. Following countries are coloured: United States of America (n = 13), United Kingdom (n = 1), France (n = 1), Italy (n = 1), Germany (n = 5), Austria (n = 1), Denmark (n = 2), Iraq (n = 1), India (n = 1), Taiwan (n = 1), Japan (2), South Korea (2). (**C**) A risk of bias summary plot for non-randomized studies with bar chart of the distribution of risk-of-bias judgments for all included studies (n = 31) across the domains of the ROBINS-I tool, shown in percentages (%) is shown. In the bottom, an overall risk of bias, which represents the collated risk-of-bias judgements for all domains, is depicted.

This systematic review was conducted using the guidelines outlined by the Cochrane Collaboration, and the Preferred Reporting Items for Systematic Reviews and Meta-Analyses (PRISMA). The completed PRISMA flowchart is shown in Fig. 1A. The literature search was carried out on the 30th of April 2022, using a search of MEDLINE, Embase, Scopus, PubMed, and JSTOR from 1943 to 2022, the complete search strategy can be found in Supplemental Digital Content 1: Supplementary Table S1. The inclusion and exclusion criteria for systematic review and meta-analysis are in Supplemental Digital Content 1: Supplementary Table S2. Only studies that compared outcomes of patients receiving conservative treatment versus early surgery were included in the meta-analysis. The definition of early surgery at the point of study selection was surgical intervention immediately after the diagnosis of spondylodiscitis (as opposed to delayed surgery). In the first abstract screening, conducted by two reviewers (SGT & ASMV), all original articles in the English language that reported on spondylodiscitis were included. Subsequently, only studies reporting on the management of spondylodiscitis which also fulfilled our inclusion criteria were included. All included papers were assessed for eligibility by two independent reviewers (SGT & ASMV). Any disagreements were resolved by consensus after discussion with a third (KV) and subsequently a fourth reviewer (RV).

### Data analysis

All relevant data were extracted manually using the Covidence data collection tool^16^. A list of extracted variables can be found in Supplemental Digital Content 1: Supplementary Table S3. In case of missing data, the respective studies’ corresponding author was contacted. All articles were critically appraised, and the risk of bias was determined against all the domains of the ROBINS-I tool by two independent reviewers (SGT & ASMV), and a consensus was reached by discussion with a third reviewer (KV)^17^. Results of the ROBINS-I analysis can be found in Supplemental Digital Content 1: Supplementary Table S4. Furthermore, the level of evidence for each included article was scored using the Oxford Centre of Evidence-Based Medicine (OCEBM) Levels of Evidence Table (Supplemental Digital Content 1: Supplementary Table S5), as well as GRADE scoring (Supplemental Digital Content: Supplementary Table S6). Definitions of early and delayed surgery used by each study are shown in Supplemental Digital Content 1: Supplementary Table S7.

An Egger’s regression and asymmetry test were used to assess publication bias (p < 0.05% = significant)^18^. Data preparation, statistical analysis, and forest plot synthesis were carried out by utilizing meta package with the R software (version 4.0.4)^19,20^. Firstly, a proportional meta-analysis was performed for mean proportions of mortality and relapse/failure among patients treated with early surgery and conservative treatment. The mortality and relapse data included both in-hospital and follow-up mortality. The most acute short-term postoperative outcome data (30 days, or 90 days) were used if longer or multiple follow-up periods were provided. All definitions of mortality and relapse/failure can be found in Table 1. Secondly, relative risk meta-analyses were computed for mortality and relapse/failure, and mean difference meta-analyses for length of stay. All outcome variable computation included 95%-CI, as well as heterogeneity measured by the I^2^ test^21,22^. The R Code used is available in Supplemental Digital Content 1: Supplementary Table S8. A detailed description of the computation, including subgroup meta-analyses, influence, and sensitivity analyses, is shown in Supplemental Digital Content 1: Supplementary File S1, and a detailed account of correlation analysis findings in Supplemental Digital Content 1: Supplementary File S2.

**Table 1:** Study characteristics of the included studies in this systematic review.

Study	Sample size	Study type and design	Country	Antibiotic used	Surgery used	Surgical approach	Indication for surgery	Definition of relapse/failure	Definition of mortality	Dropout/patients lost to follow up (LTFU)	Complications
Adogwa et al.^42^	n = 82	Cohort, R	USA	NR	Simple laminectomy Laminectomy with fusion Transpedicular decompression Anterior decompression with fusion	NR	Neurological deficit except when paraplegic or quadriplegic for > 48 h Evidence of progressive spinal deformity even in absence of neurological deficit	Worsening of the condition despite treatment given	Not defined	4.9% (4)	NR
Alas et al.^24^	n = 116	Cohort, R	USA	NR	NR	Posterior: 49.2% Combined anteroposterior: 11.3% Anterior only: 4.6%	Failure to respond to medical therapy Uncontrolled sepsis Increasing pain despite medical therapy Spinal instability due to osseous or soft tissue destruction Neurological compromise	Recurrence of the condition once this episode was deemed to be treated	30-day mortality: Death within 30 days of presentation 1 year mortality: Death Within 1 year of presentation	NR	NR
Berwick et al.^33^	n = 61	Cohort, R	USA	NR	Including but not limited to: Laminectomies with and without fusion Laminotomies with and without fusion	NR	NR	Needing surgery after failure of initial conservative therapy Relapse of the condition leading to readmission	Not defined	NR but LTFU mentioned as key limitation	NR
Canoui et al.^23^	n = 90	Cohort, R	France	Targeted towards bacteria sensitivity and culture IV antibiotics for 2 weeks, and then oral antibiotics	Drainage and debridement Laminectomy Instrumentation Iliac crest autograft (29%)	NR	Progressive neurologic deficits Progressive deformity Spinal instability with or without pain despite adequate antimicrobial therapy Persistent or recurrent bloodstream infection (without alternative source) Worsening pain despite medical therapy	Symptoms of haematogenous pyogenic vertebral osteomyelitis returning after end of antibiotic treatment with the same organism identified	Infection related deaths 12 months after presentation	NR	NR
Curry et al.^14^	n = 48	Case series, R	USA	NR	NR	NR	NR	Not defined	Not defined	NR	NR
Farber et al.^32^	n = 10,150	Cohort, R	USA	NR	Laminectomy reopening Exploration and decompression of spinal canal Excision or destruction of lesion of spinal cord Excision of intervertebral disc	NR	Reasons for **not** performing early surgery included: Deficiency anaemias Coagulopathy complicated diabetes Hypertension liver disease fluid/electrolyte disorder Obesity Renal failure	Not defined	Mortality as an inpatient	NR	Accidental puncture or laceration Hematoma Postoperative respiratory complication Foreign body inadvertently left in wound Cardiovascular complications, Wound complications Postoperative infection CSF leak Carotid or vertebral injury, Hoarseness Dysphagia Sepsis
Giampaolini et al.^48^	n = 182	Cohort, R	Italy	Vancomycin + 3rd or 4th generation cephalosporins. If allergic to above, daptomycin and quinolone	Included but not limited to: Debridement Laminectomy	Anterior Posterior	Symptoms of neurological impairment and two or more of the following: Segmental instability epidural abscess Psoas abscess Persistent fever up to temperature of 38 °C and/or sepsis Pain under appropriate analgesic treatment	Conservative: Needing surgery after failure of initial therapy Surgical: Needing further surgery after initial surgery	Not defined	15.3%	NR
Hasan et al.^25^	n = 40	Case series, R	Iraq, India	Vancomycin and ceftriaxone IV were most common	Debridement 360° fusion in 2 stages Anterior cervical discectomy and fusion Anterior cervical corpectomy and fusion (ACCF)	Debridement: posterior pedicle screws fixation 360° fusion was anterior followed by posterior Anterior cervical discectomy and fusion Anterior cervical corpectomy and fusion (ACCF)	Patients with neurologic deficit or grade II or III pyogenic spondylodiscitis that had significant destruction of the end plate and had features of instability on MRI, and failed conservative management with antibiotics at 6 weeks	Conservative: Needing surgery after failure of initial therapy Surgical: Not defined	Not defined	NR but patients who missed the 12 month follow up excluded	NR but no infectious complications after surgery
Hohenberger et al.^47^	n = 54	Cohort, R	Germany	Targeted towards bacteria sensitivity and culture	Open lumbar dorsal fusion Laminectomy	Abscess decompression was midline approach laminectomy Dorsal fusion technique was midline skin incision	Poor health Several comorbidities Signs of severe spondylodiscitis CRP > 100 mg/dl, Neurological deficits Signs of instability	Conservative: persistent lower back pain, CRP levels that remained > 100 mg/dl, development of new neurological deficits Surgical: patients with post-operative complication that needed (or may need) correction	Not defined	NR, but patients with incomplete medical records excluded	Poor wound healing (40%) Septic shock (20%) Epidural empyema (10%), Pacemaker infection (10%) Hip prosthesis infection (10%) Bone bruise of the iliac crest (10%)
Jin et al.^35^	n = 242	Cohort, R	USA	NR	Decompression Decompression with fusion	NR	Weakness (52.6%) Mechanical instability of kyphosis (21.4%) Failed medical management without instability Neurological deficit (10.4%) Failure of antibiotic Treatment (16.9%)	Conservative: Needing surgery after failure of initial therapy Surgical: Needing further surgery after initial surgery	Patients who had “died at time of chart review”	12% (33) LTFU and excluded from final analysis	Pseudoarthrosis (6/154, 3.9%) Wound washout required (5/154, 3.2%)
Jung et al.^44^	n = 92	Cohort, P	Germany	β-lactam Vancomycin/teicoplanin, or daptomycin if MSSA positive Vancomycin/teicoplanin, daptomycin, or linezolid if MRSA positive Rifampicin/Fosfomycin	Debridement (74%) Debridement with stabilization (24%), Implantation of foreign material (63%)	NR	Compression of neurological structures Mechanical instability Spinal deformity Failure of adequate conservative treatment Worse neurological deficits	Relapse of vertebral osteomyelitis or death within 1 year	Death within 1 year of admission to hospital	NR, Patients excluded if no follow up for 1 year	NR but high mortality rate following surgery noted due to various causes
Karikari et al.^41^	n = 104	Cohort, R	USA	NR	Simple laminectomy (62.5%) Laminectomy with fusion (12.5%) Transpedicular decompression (5%) Anterior decompression (with or without corpectomy) with fusion (20%)	NR	NR	Neurological worsening of condition	Death at the time of the latest follow up time of the patient (average 39.6 weeks)	3.8% (4 patients)	NR
Khanna et al.^51^	n = 41	Case series, R	USA	NR	Laminectomy with abscess drainage (66.7%) Anterior approach with bone graft fusion for (16.7%) Posterolateral approach with bone graft fusion (16.7%)	For patients undergoing bone graft fusion: Anterior approaches (n = 5) Posterolateral approach (n = 5)	Indications for **NO** surgery: No or minimal paresis (6 patients), Very poor medical condition (4 patients), Refused surgery (1 patient)	Conservative: Neurological examination showed deterioration despite adequate antibiotic therapy Surgical: Not defined	Not defined	NR	Death Sepsis Poor neurological outcome
Kim et al.^29^	n = 355	Case control, R	South Korea	NR	Surgical decompression	NR	Failed medical treatment Patient's wishes Surgeon's decision	Conservative: development of increasing back pain and/or neurologic deficits, progression of SEA on serial radiographic studies, ongoing sepsis, or death despite antibiotic treatment for more than 1 week	Mortality within 90 days of hospitalisation and in-hospital mortality	Total: 14.6% (52/355) Conservative:13% (13/100) Surgical: 15.3% (39/255)	Cardiopulmonary Death Sepsis Failed treatment
Kreutzträger et al.^43^	n = 134	Cohort, A	Germany	Targeted towards bacteria sensitivity and culture If no organism isolated, Fosfomycin and cefuroxime IV for 2 weeks, then Trimethoprim-Sulfamethoxazole and rifampin orally	Laminectomy	Dorsal approach If c-spine, ventral opening of spinal canal performed	Patients had surgery unless they refused or were not medically suitable for surgery	Requiring revision surgery	In-hospital mortality	NR	Urinary Tract Infection Decubitus Pneumonia Thromboembolism Gastrointestinal inflammation
Lee et al.^13^	n = 439	Cohort, R	South Korea	NR	Initial hemilaminectomy + drainage If major deformity or mechanical instability additional spinal instrumentation through long segment fixation with pedicle screw	Posterior approach in: 95% (n = 124/130) in non-instrumented 77% (n = 89/115) in instrumented group	Intractable pain due to abscess, substantial Aggravating neurologic deficits Major deformity or mechanical instability before or after abscess drainage	Not defined	Not defined	None, excluded if LTFU from final analysis	Complications occurred in: 31% (n = 60/194) conservatively treated patients 26% (n = 64/245) surgically treated patients (p = 0.267)
Lener et al.^26^	n = 197	Case–control, R	Austria	NR	Decompression Decompression and instrumentation with or without (partial) corpectomy	NR	Neurological deficits Progressive or intractable pain Radio- logical progression due to MRI findings despite maximum conservative treatment qualified for surgical treatment	Not defined	Not defined	NR	NR
Mann et al.^40^	n = 24	Case series, P	Germany	Initial treatment of intravenous antibiotics included dicloxacillin, clindamycin, and gentamicin Therapy adjusted to results of microbiological study thereafter	Spinal cord decompression Debridement of the affected vertebral body Realignment by transpedicular/intracorporal spondylodesis	Ventral Approach: 25% (n = 6/24) Dorsal Approach 17% (n = 4/24) Combined ventral and dorsal approach 58% (n = 14/24)	All patients underwent surgery Immediate surgical management was performed in patients with rapidly progressing neurological deficit	Not defined	Not defined	NR	Wound infection 16% (n = 4/24) Allergic reaction 4% (n = 1/24) Renal failure 4% (n = 1/25) Colitis 21% (n = 5/24) Fungal infection 21% (n = 5/24) Urinary tract infection 21% (n = 5/24) Pneumonia 8% (n = 2/24) Decubitus ulcers 16% (n = 4/24)
McHenry et al.^39^	n = 253	Cohort, R	USA	NR	NR	Anterior Posterior Posterolateral	Drainage of abscesses Relief of compression of the spinal cord, cauda equina, or nerve roots Spinal stabilization, debridement Excision of sinuses Removal of infected hardware Resection of contiguous infected aortic aneurysms or grafts with extra-anatomic bypass	Not defined	Associated or caused by persistent infection	NR	NR
Pitaro et al.^34^	n = 700	Cohort, R	USA	NR	NR	NR	NR	Not defined	Not defined	NR	NR
Shweikeh et al.^31^	n = 16	Cohort, R	USA	NR	87.5% (n = 7/8) of surgically treated patients had an anterior cervical fusion and discectomy, of which 2 also received a corpectomy 12.5% (n = 1/8) of surgically treated patients had a posterior decompression, and also received a corpectomy	Anterior Posterior	NR	Not defined	Not defined	25 (n = 4/16)	NR
Segreto et al.^28^	n = 34,465	Cohort, R	USA	NR	NR	Anterior Posterior Combined	NR	Not defined	Not defined	NR	Most common complication in same-day-surgery patients was anaemia (6.1%) Infection was the most common complication in patients with 1-day delay (7.9%) Subsequently, sepsis was the most common complication for each successive surgical delay group (range: 8.2%–19.3%)
Sobottke et al.^46^	n = 20	Case series, R	Germany	NR	Single 2-stage and ventral Dorsal approach	40% (n = 4/10) dorsal approach 10% (n = 1/10) ventral 10% (n = 1/10) one-stage surgery via combined ventrodorsal approach 40% (n = 4/10) two-stage surgery via dorsoventral approach	NR	Not defined	Not defined	45 (n = 9/20)	1 patient with re-spondylodesis after screw pull-out 1 patient with decubital ulcer, sepsis, psychosis, infect-associated anaemia 1 patient with stroke, aspiration pneumonia and death 1 patient with Psychosis 1 patient with Pneumothorax after central venous catheter 1 patient Infect exacerbation 1 patient with relapse 1 patient with dural tear and depression
Sur et al.^38^	n = 55	Case series, R	UK	Multiple (Flucloxacillin, vancomycin, piperacillin/ tazobactam, meropenem, gentamicin, teicoplanin, benzylpenicillin, rifampicin, fusidic acid, flucloxacillin co-trimoxazole, doxycycline, clindamycin, isoniazid, rifampicin and ethambutol)	NR	NR	Development of neurological deficit Significant bony destruction Progressive deformity Development of any epidural abscess at cord level Cord compromise Intolerable pain not responding to medical control	Not defined	Not defined	NR	NR
Tang et al.^30^	n = 46	Case–Control, R	Taiwan	NR	Posterior decompression and abscess drainage	Posterior	NR	Not defined	Not defined	NR	NR
Tani et al.^49^	n = 34	Cohort, P	Japan	Linezolid was selected as the post-biopsy antibiotic regimen prior to the establishment of a microbiologic diagnosis	Isolated PPS–Rod Fixation: 20.6% (n = 7/34) PPS–Rod Fixation + Posterior Decompression and Debridement + Surgical Sampling: 35.3% (n = 12/34) LLIF Procedure for Anterior Debridement with Iliac Bone Grafting: 8.8% (n = 3/34)	Posterior Transpsoas	Increased CRP and/or persistent or worsening posture-related back pain who did not respond to conservative management complicated pyogenic spondylodiscitis, with or without impending sepsis extensive anterior column destruction with potential instability and local kyphosis unsuccessful eradication of infection by posterior-based surgeries	Not defined	Not defined	11.8 (n = 4//34) (2 died, 2 lost to follow up)	NR
Tsai et al.^52^	n = 90	Cohort, R	Taiwan	NR	Transforaminal lumbar or costotransversect-omy thoracic approach interbody debridement from the posterior approach and fusion in 16.3% (n = 7/43) patients managed surgically Anterior radical debridement and fusion was performed in 32.6% (n = 14/43) surgically managed patients Anterior procedure completed with posterior instrumentation and fusion was carried out in 51.2% (n = 22/43) surgically managed patients	Anterior Transforaminal lumbar or costotransversect-omy thoracic approach	Patients who were diagnosed with an early-stage infection, had no signs of neurological deficit, and had Pittsburgh bacteraemia score of no more than 4	Not defined	Not defined	None	NR
Uchida et al.^50^	n = 37	Cohort, R	Japan	NR	Mini-incisional percutaneous suction drainage Mini-incisional curettage Major open surgery	Included: left sided retroperitoneal approach Psoas abscess surgically treated via anterior extraperitoneal approach	Collection of intradiscal fluid Suppurative lesions in epidural space, disc space, or parts of endplate Extensive destruction of anterior and middle columns Extension of suppurative lesions into spinal canal Failure of antibiotic treatment Neurological Complications	Conservative: Poor response to antibiotics Surgical: Not defined	Not defined	NR	3/7 patients still had neurological complications remaining after surgery
Valancius et al.^37^	n = 196	Cohort , R	Denmark	Dicloxacillin then adjusted following identification of microorganisms Cefuroxime used in cases of penicillin intolerance	Posterior debridement alone in 16.2% (n = 19/117) of surgically managed patients Posterior debridement with pedicle screw instrumentation in 64.1% (n = 75/117) of surgically managed patients Anterior debridement alone in 6.0% (n = 7/117) of surgically managed patients Anterior debridement combined with pedicle screw instrumentation in 13.7% (n = 16/117) of surgically managed patients	Real anterior-retroperitoneal Transthoracic Smith–Robinson approaches Posterior midline Anterolateral	Neurologic compromise Significant vertebral body destruction with segmental instability Epidural abscess formation Intractable back pain Failure of medical treatment	Not defined	Not defined	Conservative: n = 11 (8 died, 3 unknown) Surgical: n = 16 (5 non-infection morality,8 unknown, 3 followed up at another site)	9 Pneumonia 7 Pleuritis 5 Hypersensitivity reaction to dicloxacillin 4 Atrial fibrillation 4 Renal failure 3 Deep venous thrombosis 2 Ileus/bowel obstruction 2 Ischemic brain stroke 1 Meningitis 2 Pneumothorax (due to central venous catheter) 1 Pyelonephritis
Verla et al.^45^	n = 16	Case series, R	USA	NR	Laminectomy	Posterior-only Combined anterior–posterior approach	Indication for those who underwent initial laminectomy not given Indication for further instrumentation was if: Kyphosis progressed to greater than 108 increase from baseline at diagnosis Destruction of vertebral end plates by more than 50% Worsening stenosis at the apex of kyphosis Uncontrolled pain Progressive neurologic deficits	Not defined	Not defined	None, LTFU patients excluded from study	NR
Zadran et al.^27^	n = 125	Cohort, R	Denmark	NR	Debridement followed by circumferential fusion with posterior instrumentation, bone grafting (posterolateral and interbody), and then locally instilled antibiotics	NR	Mechanical instability Neurologic deficit Compression of spinal cord or cauda equina Epidural abscess formation Paravertebral abscess not suitable for ultrasound guided drainage Intolerable pain Failed nonsurgical therapy	Not defined	Not defined	None	NR

In this table, the study characteristics of all included studies in this systematic review (n = 31) are summarised. Following variables were extracted: Study (author and date of publication), sample size, study type and design, country, antibiotic used, surgery (surgical technique) used, surgical approach, indication for surgery, definition of relapse/failure, definition of mortality, dropout / patients Lost To Follow Up (LTFU) and complications.

*A* ambispective, *R* retrospective study, *P* prospective study, *CSF* cerebrospinal fluid, *MRSA* methicillin resistant *Staphylococcus aureus*, *LTFU* lost to follow-up, *NR* data not reported.

This systematic review was registered on PROSPERO CRD42022312573 under the title “Early surgical intervention vs expectant management in spondylodiscitis: a systematic literature review and meta-analysis” on the 28th of February 2022.

## Results

A total of 13,209 studies were screened. From these, 75 full texts were assessed using our inclusion criteria. A total of 31 studies were included in this systematic review. From these, 21 studies were also included in the meta-analysis (Fig. 1A). The total pooled sample size of the systematic review was 48,504 and the overall pooled sample size of the meta-analysis was 10,954 patients. A world map of publication origins is shown in Fig. 1B.

**Figure 2:**
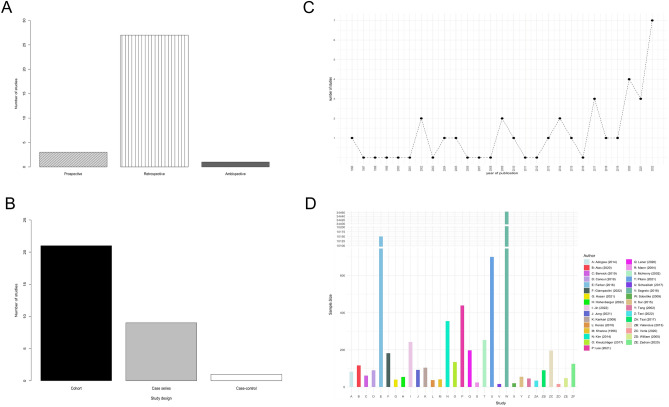
(**A**) Bar plot visualizes the number of prospective (n = 3), retrospective (n = 27) and ambispective (n = 1) studies included in the systematic review (n = 31)^13,14,23–52^. (**B**) Bar plot visualizes the number of included studies (n = 31) that are cohort studies (n = 21), case series (n = 9) and case–control studies (n = 1). (**C**) Line plot displays the number of studies for the following years of publications: 1996 (n = 1), 2002 (n = 2), 2004 (n = 1), 2009 (n = 2), 2010 (n = 1), 2013 (n = 1), 2014 (n = 2), 2017 (n = 3), 2017 (n = 1), 2018 (n = 1), 2019 (n = 1), 2020 (n = 4), 2021 (n = 3), 2022 (n = 7). Each year is indicated as black circle, and the circles are connect by an interrupted line to visualise the trend more clearly. (**D**) Bar plot shows the sample size of each included study in the systematic review (n = 31). Studies are named alphabetically A–Z, each letter refers to the cited studies in synchronized order, which is furthermore depicted in the legend on the right of the graph. The bar plot is interrupted to allow for adequate visualisation of all data points.

**Figure 3:**
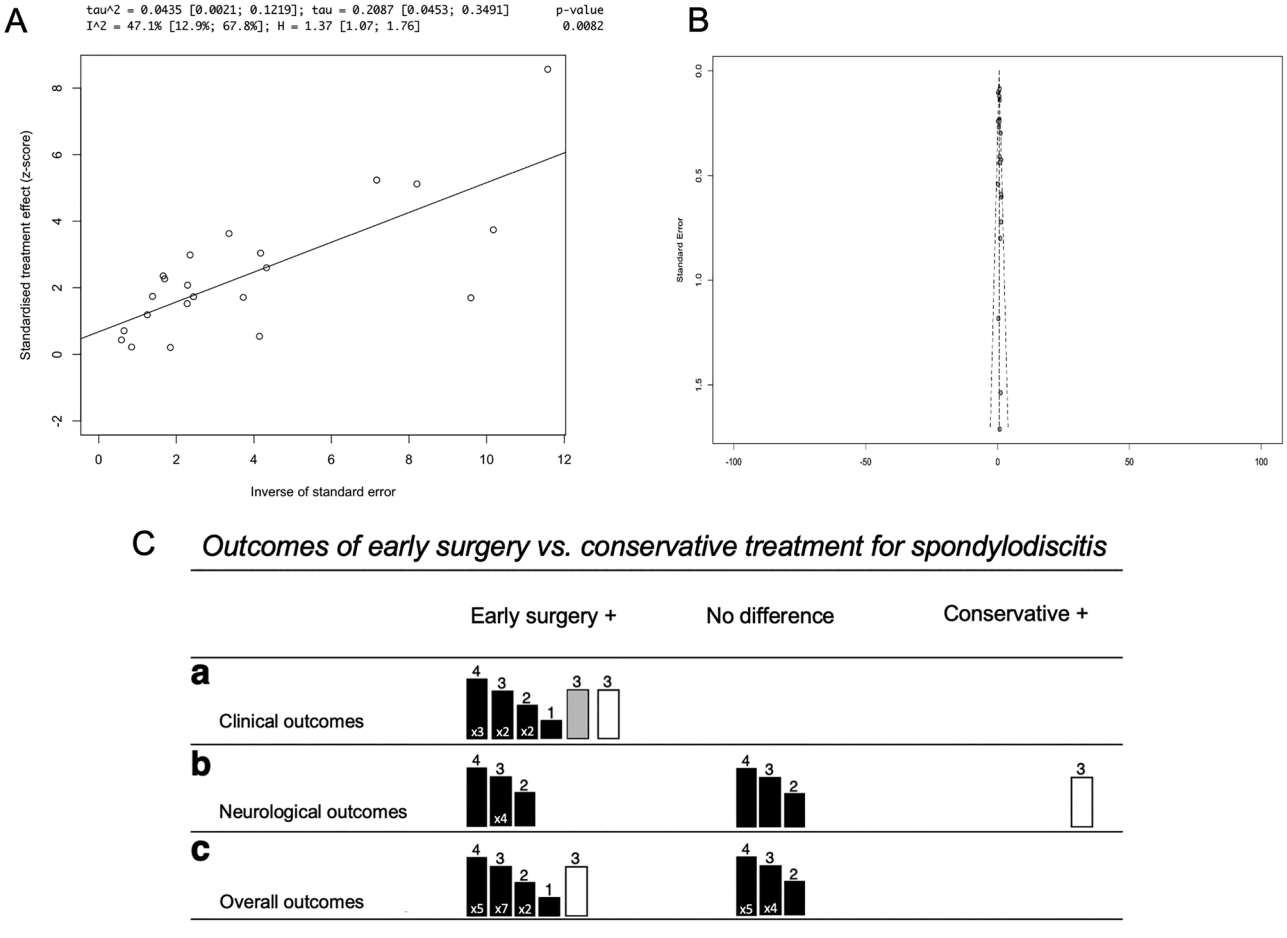
(**A**) An Egger’s asymmetry plot of all data points included in the meta-analysis (n = 21 studies)^13,23–26,29,32–35,37,38,41–43,47,48,50,52^; the x-axis represents the inverse of standard error, and the y-axis the standardized treatment effect (as z-score). Furthermore, at the top of the graph different parameters of heterogeneity, including I^2^, are shown. P-value < 0.05 is deemed to be significant and implicates publication bias. Egger’s asymmetry test yielded p = 0.0082, calculated running an Egger’s regression (see Egger’s regression line) on the collated DOR and standard errors of all data used in the meta-analysis (n = 21), indicating significant publication bias. (**B**) A funnel plot is shown, which plots every study included in the meta-analysis (n = 21). The observed effect sizes (diagnostic odds ratio) are on the x-axis against a measure of their standard error on the y-axis. All studies fall roughly within the parameters of the funnel plot, there are no gross outliers, indicating that there is no individual studies skewing the publication bias regression analysis. (**C**) The effects of early surgery versus conservative treatment for spondylodiscitis in terms of: (a) clinical [non-neurological] outcomes, (b) neurological outcomes, (c) overall outcomes, are visualized as harvest plot. The effects are stratified intro three columns: early surgery has better outcomes than conservative treatment (“Early surgery +), there is no difference between the two treatment modalities (“No difference”) and conservative treatment has better outcomes than early surgery (“Conservative +). A rectangle represents a single study, unless at bottom of the rectangle a number is specified as i.e. × 2 (= two studies). The colours of the rectangles correspond to the study design: black (retrospective), grey (ambispective), white (prospective). The number on top of the rectangle specifies the risk of bias in overall risk of bias (in line with risk of bias analysis, with 4 implying low risk of bias, 3 implying moderate risk, 2 serious risk and 1 critical risk). The height of the rectangle directly correlates to the risk of bias in outcome measurement, and the aforementioned number on top of the rectangle. Definitions for clinical and neurological outcomes are as follows: Clinical outcomes pools different definitions used by different studies including prognosis, recurrence, hospital stay, mortality rates, and lab parameters. Further in-depth investigation of these can be seen in the meta-analysis. On the other hand, the definition of neurological outcomes was split in two categories—the first being the presence or absence of neurological deficits, and the second being a graded scale of neurological deficits based on the American Spinal Injury Association Scale (ASIA scale).

Out of the 31 included studies, 14 were deemed to have a ‘low’ risk of bias^23–36^; 11 a ‘moderate’ risk of bias^13,14,37–45^; 6 a ‘serious’ risk of bias^46–51^, and 1 study had a ‘critical’ risk of bias^52^ using the ROBINS-I tool^17^. A scoring explanation is available in Supplemental Digital Content 1: Supplementary Table S4, a graphical summary in Fig. 1C. The OCEBM guidance was used to determine the level of evidence of each study. 21 studies were classified as 2b, three studies as level 3b, and seven studies as level 4 (Supplemental Digital Content 1: Supplementary Table S5). The GRADE scoring is shown in Supplemental Digital Content 1: Supplementary Table S6 and showed that, in terms of the study findings’ probability of being close to the estimated effect, 17 studies scored as moderate, 6 studies as high, 7 studies as low, 1 study as very low. The study characteristics are detailed in Table 1, and the main findings from each study are demonstrated in Table 2 (excluding studies that focused on purely spinal epidural abscesses) and Table 3 (including only studies that focused on purely spinal epidural abscesses). Study characteristics are additionally graphically presented in Fig. 2A–D. Egger’s asymmetry plot (Fig. 3A) yielded that there was a significant publication bias (p = 0.0082), however, a funnel plot (Fig. 3B) showed that there were no individual studies that skewed the publication bias regression analysis.

**Table 2:** Detailed summary of the results from each of the included studies in this systematic review (excluding studies that focused only on spinal epidural abscesses).

Study	Treatment	Positive cultures	Epidural abscess	Duration of antibiotic treatment	Additional surgical treatment required	Length of hospital stay (days)	Relapse/failure	Mortality	Main conclusion	Risk of bias
Alas et al.^24^	Conservative vs Surgery	90.5%	33.6%	NR	Surgical: 11.6% (n = 5/43)	Conservative: 40.5 ± 30.7 Surgical: 23.9 ± 18.2	Surgical: 11.6% (n = 5/43) Conservative: 16.4% (n = 12/73)	30 Day: Conservative: 17.8% (n = 13/73) Surgical: 2.3% (n = 1/43) 1 year: Conservative: 20.5% (n = 11/73) Surgical: 11.6% (n = 5/43)	Surgical intervention was associated with a lower mortality after 30 days and a lower mortality after 1 year when compared to patients who were treated conservatively A higher modified frailty index score was associated with higher mortality rates regardless of using surgical or conservative treatment methods	D1: Moderate D2: Moderate D3: Low D4: Low D5: Low D6: Low D7: Low D8: Low
Canoui et al.^23^	Conservative vs Surgery	NR	50%	Both groups: IV antibiotics for at least 2 weeks followed by oral antibiotics. Recommended duration was 12 weeks in total	NR	NR	Relapse: Surgical: 4% (n = 1/28) Conservative: 5% (n = 3/62)	Surgical: 11% (n = 3/28) Conservative: 11% (n = 7/62)	Surgery is a safe and effective treatment for pyogenic vertebral osteomyelitis However, patients undergoing surgery had similar outcomes to those who underwent nonsurgical treatment with respect to neurological and pain outcomes	D1: Moderate D2: Low D3: Low D4: Low D5: Low D6: Low D7: Low D8: Low
Giampaolini et al.^48^	Conservative with Antibiotic Treatment for < 6 weeks vs Conservative with antibiotic treatment for > 6 weeks vs Surgery	46.8%	NR	Conservative: one group > 6 weeks and second group < 6 weeks	Surgical: 8.7% (n = 6/69) Conservative: 7.8% (n = 10/146)	NR	Failure: Surgical: 8.7% (n = 6/69) Conservative: 7.8% (n = 10/146)	NR	Surgical treatment should be considered by the treating physician for patients if after 4 weeks of conservative therapy, there is not a reduction in the ESR levels to < 50 mm/h and CRP levels to < 2.7 g/dl The comparison between Surgical and conservative patients showed a reduction in the CRP at 4 weeks and better VAS score for pain at 3 months post-op in the Surgical cohort	D1: Critical D2: Moderate D3: Low D4: Low D5: Low D6: Low D7: Low D8: Serious
Hasan et al.^25^	Conservative vs Surgery	Blood culture: 42.5% Tissue culture: 82.5%	NR	12 weeks	Conservative: 11.5% (n = 3/26)	NR	Failure: Conservative: 11.5% (n = 3/26) Surgical: NR	NR	In patients with Pyogenic Spondylodiscitis, an excellent outcome was reported in both the Surgically and the conservatively treated groups There was no difference between the two groups in terms of the clinical outcomes using the COMI score, the ESR levels, or the CRP levels between the two groups at 12 months follow up	D1: Low D2: Moderate D3: Low D4: Low D5: Low D6: Low D7: Low D8: Low
Hohenberger et al.^47^	Conservative vs Surgery	55%	NR	IV antibiotics: average 4.5 weeks (31 days), followed by oral antibiotics average: 9.5 weeks	Conservative: 55.2% (n = 16/29) (these patients are therefore subsequently included in the surgical group)	Conservative: 30.3 Surgery 46.7	Failure Conservative: 55.2% (n = 16/29) Surgical group: 9.8% (n = 4/41)	NR	Patients with lumbar spondylodiscitis and certain other chronic conditions would benefit from early instrumented fixation when compared to patients who had late fusion or abscess evacuation only, resulting in an accelerated recovery, having a shorter hospital stay, and a better quality of life Patients who only have moderate CRP level increases and have no neurological deficits or any severe comorbidities would be suitable for antibiotic/conservative treatment	D1: Critical D2: Moderate D3: Low D4: Low D5: Low D6: Low D7: Low D8: Serious
Jin et al.^35^	Conservative vs Surgery	55.2%	72.7%	NR	Conservative: 31% (n = 41/129) (31%); Surgical: 11.7% (n = 18/154)	NR	Failure: Conservative: 31.8% (n = 41/129) Surgical: 11.7% (n = 18/154)	Conservative: 14.8% (13/88) Surgical: 9.7% (15/154)	The presence of epidural abscess, cervical involvement of the spine, thoracic involvement of the spine, and higher number of spinal levels that were involved in the disease were potential risk factors for the need for surgery in this study The lab values of ESR, CRP, WBC, creatinine, and albumin were not associated with an increased risk of requiring surgery	D1: Moderate D2: Moderate D3: Low D4: Low D5: Low D6: Low D7: Low D8: Low
Jung et al.^44^	Conservative vs Surgery	63%	62%	Median of 34 days [21–53 IQR] in hospital antibiotics	NR	NR	Total failure: 48% (n = 44/92) Total relapse: 2% (n = 2/92)	Total: 45.7% (n = 42/92)	Treatment failure is high in patients with *S. aureus* vertebral osteomyelitis patients primarily because of patient mortality within 3 months of diagnosis Risk factors include older age, having other comorbidities, and having neurologic deficits Surgery was associated with a better prognosis in patients with *S. aureus* vertebral osteomyelitis	D1: Serious D2: Low D3: Low D4: Low D5: Low D6: Low D7: Low D8: Moderate
Kreutzträger et al.^43^	Conservative vs Surgery	67%	79.1%	> 6 weeks	Surgical: 16% (n = 17/109)	Combined: 36.8	Failure: Conservative: NR Surgical: 16% (n = 17/109)	Overall: 11.2% (n = 15/134) Conservative: 20% (n = 5/25) Surgical: 9.2% (n = 10/109)	Mortality rate higher in patients receiving conservative treatment than those receiving surgical treatment However, patients who received conservative treatment tended to have a poorer health which was the reason they were not suitable for surgery, and this may impact the results	D1: Serious D2: Moderate D3: Low D4: Low D5: Low D6: Low D7: Low D8: Moderate
Lee et al.^13^	Conservative vs Surgery (antibiotics and surgery—instrumented or non-instrumented)	NR	53% of non-operative 88% of non-instrumented 97% of Instrumented	NR	NR	Conservative: 60 (53–72) Instrumented Surgery: 54 (47–63) Non instrumented surgery: 52 (47–59)	Recurrence in: Conservative: 11% (n = 22/194) Surgical: 12% (n = 30/245) (p = 0.771)	Overall 90 day mortality: Conservative: 8% (n = 15/194) Surgical: 10% (n = 24/245) Overall 1 year mortality: Conservative: 15% (n = 30/194) Surgical: 23% (n = 56/245) (p = 0.053)	Surgical instrumentation can be performed if indicated within reasonable risk	D1: Moderate D2: Serious D3: Low D4: Low D5: Moderate D6: Low D7: Low D8: Moderate
Lener et al.^26^	Conservative vs Surgery (antibiotics and surgery)	24%	32.5%	NR	NR	NR	NR	Deaths due to septic multiorgan failure occurred in: Conservative: 35% (n = 6/17) Surgical: 24% (n = 4/17) (p < 0.05)	Mortality rate was significantly greater in patients solely receiving conservative treatment	D1: Low D2: Moderate D3: Low D4: Low D5: Low D6: Low D7: Low D8: Low
Mann et al.^40^	Conservative (delayed surgery) vs Surgery (antibiotic and immediate surgery)	58%	62.5%	Minimum of 10 days of intravenous administration, followed by oral antibiotics for 3 months	NR	Combined: 33	Failure in 12% (n = 2/17) of patients treated conservatively	Mortality occurred in 8% (n = 2/24) of patients	Surgical treatment offers advantages of spinal cord decompression, immediate mobilization, and correction of spinal deformity and is the modality of choice in patients with acute spinal osteomyelitis	D1: Critical D2: Low D3: Low D4: Low D5: Low D6: Low D7: Low D8: Moderate
McHenry et al.^39^	Conservative vs Surgery (antibiotics and surgery)	69% -aspiration or biopsy 78%—surgical culture	17%	90% of those in conservative group received more than or equal to 4 weeks	NR	NR	Recurrence occurred in 14% (n = 36/253) of patients	Infection related mortality occurred in 11% (n = 29/253) of patients	Prolonged antimicrobial therapy and well-judged timely surgery are essential for optimal outcome	D1: Serious D2: Low D3: Low D4: Low D5: Low D6: Low D7: Low D8: Moderate
Segreto et al.^28^	Surgery: same day vs 1,2,3–6,7–14,14–30 day delay	NR	NR	NR	NR	Same day surgery: 4.2 Day 1 surgery 10.1 Day 2 surgery: 11.2 Day 3–6 surgery 14.0 Day 7–14 surgery: 20.6 Day 14–30 surgery: 34.0	NR	Mortality in same-day-surgery patients was 0.3%, with experiencing increased mortality rates in successive surgical delay groups, peaking at 5.5% (14–30-day delay, P < 0.001)	Surgery within 24 h of admission is more likely to have desirable outcomes. Delays in surgery had a significantly increased risk of complication, mortality, and neurologic deficits	D1: Moderate D2: Moderate D3: Low D4: Low D5: Low D6: Low D7: Low D8: Low
Sobottke et al.^46^	Conservative vs Surgery	75%	33.3%	NR	Conservative treatment failed in 2 patients and had to be treated surgically	Combined: 65.3	Relapse in: 10% (n = 1/10) in surgically treated patients (occurred due to patient noncompliance) 0% (n = 0/10) in conservatively managed patients	In patient: 1 in conservative group Outpatient: 1 conservative 2 surgical	Surgical management of spondylodiscitis in HIV- positive patients is not associated with an increased complication rate	D1: Critical D2: Moderate D3: Low D4: Low D5: Low D6: Low D7: Low D8: Serious
Sur et al.^38^	Conservative vs Surgery	84%	NR	NR	NR	Conservative: 42 (range 5–149) Surgical: 59 (range 9–209)	NR	NR	Intravenous antibiotic therapy is effective in managing adult spontaneous spondylodiscitis	D1: Serious D2: Moderate D3: Low D4: Low D5: Low D6: Low D7: Low D8: Moderate
Tani et al.^49^	Conservative vs Surgery	65%	47%	NR	NR	NR	NR	Overall mortality in 5.9% (n = 2/34) of patients	Provision of local stabilization without contamination of metalwork was possible with non-fused PPS–rod placements into infection-free vertebrae alone or in combination with posterior debridement. In cases of extensive vertebral body destruction, MIS LLIF allowed for direct access to the infected focus for bone grafting	D1: Critical D2: Moderate D3: Low D4: Low D5: Low D6: Low D7: Low D8: Serious
Tsai et al.^52^	Conservative vs Surgery (antibiotics and surgery)	NR	NR	2 to 4 weeks for patients treated surgically NR for patients treated conservatively	4.6% (n = 2/43) of patients managed surgically required further debridement	Conservative: 51.2 ± 23.2 Surgical: 33.4 ± 17.5	Failure requiring further surgery in: 4.6% (n = 2/43) in surgical group 0% (n = 0/47) in conservative	None reported^$^	Early surgical treatment achieves a better prognosis, shorter hospitalization period, and significant improvement in kyphotic deformity and quality of life	D1: Low D2: Low D3: Low D4: Low D5: Critical D6: Low D7: Low D8: Critical
Valancius et al.^37^	Conservative vs Surgery	NR	Surgical: 51.2% Conservative: 16.4%	NR	Conservative treatment failed in 13.1% (n = 12/91) patients and thus required surgery 20.5% (n = 24/117) of surgically managed patients required reoperation	NR	Conservative: 13.1% (12/91) Surgical: 20.5% (24/117)	Infection related mortality occurred in: Conservative: 3% (n = 3/91) Surgical: 0% (0/117)	For selected patients without spondylodiscitic complications, conservative measures are safe and effective, failure of conservative therapy requires thorough surgery	D1: Serious D2: Moderate D3: Low D4: Low D5: Low D6: Low D7: Low D8: Moderate
Verla et al.^45^	Conservative vs Surgery (antibiotics and surgery)	81.3%	NR	NR	Conservative treatment failed in 33.3% (n = 4/12) patients and required surgery Further surgery was required in 100% (n = 4/4) patients initially managed surgically	Surgical: 22.9 (range 10–58) Conservative: NR	See previous cell	NR	Long segment instrumentation in addition to decompression at initial surgery may be beneficial especially due to proclivity for kyphotic deformity at the TL junction. Laminectomy alone may increase progression of kyphosis	D1: Low D2: Moderate D3: Low D4: Low D5: Serious D6: Low D7: Low D8: Moderate
Zadran et al.^27^	Conservative vs Surgery (antibiotics and surgery)	NR	NR	IV antibiotics for 10 to 14 days and oral antibiotics for a duration of 10 weeks for an overall duration of 3 months	NR	NR	NR	Overall mortality: at 1 year:13.6% (n = 17/125) at 2 year 16.8% (n = 21/125)	No difference in mortality between patients undergoing surgical management for vertebral osteomyelitis according to standardized and agreed-upon guidelines and those managed conservatively	D1: Low D2: Moderate D3: Low D4: Low D5: Low D6: Low D7: Low D8: Low

In this table, a detailed summary of the results from each of the included studies in this systematic review (excluding studies that focused only on spinal epidural abscesses) is presented. Following variables were extracted: study (author and date of publication), treatment, positive cultures (%), epidural abscess (%), duration of antibiotic treatment, additional surgical treatment required, length of hospital stay (days), relapse/failure, mortality, main conclusion of the article, and risk of bias scoring. The number of patients who required additional surgery after having had an initial treatment regimen or procedure is shown in the ‘Additional Surgical treatment required’ column. The number of patients who had a relapse or representation of their condition or failed their initial treatment regimen or procedure is shown in the ‘Relapse/failure’ column. The bias of each study is calculated by the ROBINS-I tool is shown in the ‘Bias’ column.

*NR* data not reported, *IV* intravenous, *D1* bias due to confounding, *D2* bias due to selection of participants, *D3* bias in classification of interventions, *D4* bias due to deviations from intended interventions, *D5* bias due to missing data, *D6* bias in measurement of outcomes, *D7* bias in selection of the reported result, *D8* overall bias, *COMI* core outcome measure index, *ESR* erythrocyte sedimentation rate, *CRP* C-reactive protein, *WBC* white blood cell count, *IQR* inter-quartile range, *PPS* percutaneous pedicle screw, *MIS-LLIF* minimally invasive spine surgery-lateral lumbar interbody fusion, *TL* thoracolumbar.

**Table 3:** Detailed summary of the results from each of the included studies in this systematic review that only focussed on spinal epidural abscesses.

Study	Treatment	Positive cultures	Epidural abscess	Duration of antibiotic treatment	Additional surgical treatment required	Length of hospital stay (days)	Relapse/failure	Mortality %	Main conclusion of the article	Risk of bias
Adogwa et al.^42^	Conservative vs Surgery	87.8%	100%	NR	NR	NR	Poor outcome: Conservative: 1.9% (1/52) Surgical: 6.7% (2/30)	Conservative: 21% (11/52) Surgical: 30% (9/30)	Surgical decompression and antibiotic treatment combined does not have a better clinical outcome than antibiotic treatment alone when treating patients over the age of 50 with epidural abscesses	D1: Serious D2: Moderate D3: Low D4: Low D5: Low D6: Low D7: Low D8: Moderate
Berwick et al.^33^	Conservative (antibiotics only or antibiotics with interventional radiology paraspinal abscess aspiration) vs surgical	82% (identifiable pathogen)	100%	In hospital: Overall: 23.8 Days Conservative: 35.0 days Surgical: 13.6 days At Home: Overall: 84.9 days Conservative: 43.2 days Surgical: 123.6 days	Conservative: 9/29 (31%)	Conservative: 19.9 ± 30.7 Surgical: 12.6 ± 10.8	Relapse with readmission: Conservative: 28% (8/29) Surgical 25% (8/32)	NR	Almost 1/3 of patients initially receiving conservative treatment and needed surgery However, there was no statistically significant differences in outcomes between the two groups Patients with a history of infections may need more aggressive treatment, but further research needed to draw a conclusion as to the best management strategies	D1: Moderate D2: Moderate D3: Low D4: Low D5: Low D6: Low D7: Low D8: Low
Curry et al.^14^	Conservative vs Surgery	60.4% (n = 29/48)	100%	NR	Conservative treatment failed in 47.8% (n = 11/23) patients and required surgery	NR	8% (n = 2/25) worsened in surgical group 52% (n = 12/23) worsened in conservative group	NR	Patients treated without early surgery were significantly at more risk to deteriorate and suffer poor outcomes	D1: Serious D2: Moderate D3: Low D4: Low D5: Low D6: Low D7: Low D8: Moderate
Farber et al.^32^	Conservative vs Early Surgery (< 48 h) vs delayed surgery	NR	100% (include intraspinal abscesses)	NR	NR	Conservative: 17.3 ± 16.6 Early Surgical: 14.1 ± 14.7 Delayed Surgical: 22.8 ± 19.8	NR	During hospitalisation only: Early surgical 2.7% (n = 173/6281) Delayed Surgical: 5.01% (n = 158/3167) Conservative: 8.6% (n = 31/702)	Patients undergoing medical management for intraspinal abscesses have fewer complications However, those that undergo early surgical management within 48 h of presentation have better outcomes, and lower healthcare costs Therefore, early diagnosis and intervention is important for intraspinal abscess patients	D1: Moderate D2: Moderate D3: Low D4: Low D5: Low D6: Low D7: Low D8: Low
Karikari et al.^41^	Conservative vs Surgery	88.50%	100%	> 6 weeks	NR	NR	Worsening outcomes: Conservative: 5% (3/62) Surgical: 5% (2/38)	Conservative: 17% (11/62) Surgical: 23% (9/38)	No statistical evidence to suggest that patients who do not undergo early surgery have a worse prognosis than those who do undergo early surgery Surgical management is not superior to conservative management The data in this study suggests that the treatment plan should be partly determined by the anatomy of the SEA because this makes a difference for the treatment plan	D1: Serious D2: Moderate D3: Low D4: Low D5: Low D6: Low D7: Low D8: Moderate
Khanna et al.^51^	Conservative treatment (including needle aspiration) vs Surgery (open surgery)	92.7%	100%	NR	Conservative: 31.25% (5/16)	NR	Failure: Conservative: 50% (8/16) Poor outcomes: Conservative: 36.4% (4/11) Surgical: 43.3% (13/30)	Total: 19.5% (8/41)	Surgical drainage and antibiotics is the recommended treatment However, the authors note that conservative treatment with antibiotics alone can also be used for certain patients These patients were selected using a grading system taking into account the age of the patient, the degree of the thecal sac compression, and the duration of the patient's symptoms	D1: Serious D2: Moderate D3: Low D4: Low D5: Low D6: Serious D7: Low D8: Serious
Kim et al.^29^	Conservative vs Surgery	92.1%	100%	IV for at least 6–8 weeks, followed by oral antibiotics	Conservative: 29.6% (42/142)	NR	Failure: Conservative: 38% (54/142)	Overall: 14.6% Conservative: 14% (14/100) Surgical: 10.2% (26/255)	SEA that is treated with non-operative treatment only has a very high risk for failure if the following risk factors are present: age > 65 years with diabetes, MRSA infection, neurologic compromise If there are none of these risk factors, a clinician can consider non-operative treatment in these patients	D1: Low D2: Low D3: Low D4: Low D5: Low D6: Low D7: Low D8: Low
Pitaro et al.^34^	Conservative vs Surgery	NR	100%	NR	NR	Conservative: 11.2 ± 11.4 Surgical:: 14.1 ± 12.6	30-day readmission rate: 17.1% (n = 60/350) in surgically managed patients 21.4% (n = 75/350) in conservatively managed (p = 0.15) 90-day readmission rate: 26.0% (n = 91/350) in surgically managed patients 35.1% (n = 123/350) in conservatively managed patients (p < 0.05)	None^$^	In patients with a low comorbidity burden, readmission rate was significantly lower for surgically managed patients than conservatively managed patients	D1: Moderate D2: Moderate D3: Low D4: Low D5: Low D6: Low D7: Low D8: Low
Shweikeh et al.^31^	Conservative vs Surgery	NR	100%	NR	NR	Conservative: 9.4 Surgical: 8.25	NR	Mortality occurred in 12.5% (n = 2/16) of patients	Both patient factors and multidisciplinary efforts should be considered to improve prognosis in patients with cervical spine epidural abscess	D1: Moderate D2: Moderate D3: Low D4: Low D5: Low D6: Low D7: Low D8: Low
Tang et al.^30^	Conservative vs Surgery	70% (32/46)	100%	NR	NR	Combined: 32	Relapse in 15% (n = 7/46) of patients	Overall mortality in 10.9% (n = 5/46) of patients	Immediate MRI warranted for febrile patients with localized back pain with significant risk of epidural abscess. Presence of thrombocytopenia, evidence of spinal cord compression or extremely elevated ESR should prompt aggressive treatment	D1: Low D2: Moderate D3: Low D4: Low D5: Low D6: Low D7: Low D8: Low
Uchida et al.^50^	Conservative vs Surgery	NR	100%	NR	Conservative: 62.5% (16/26)	Conservative: 79.4 ± 21.1 Surgical: 43.0 ± 18.2	Failure needing surgery: Conservative: 62.5% (16/26)	0—reported that there was no mortality in their patient cohort	Surgical drainage with continuous intravenous antibiotic management is the preferred mode of treatment Neurological prognosis is better for patients who are treated with surgical treatment without delay	D1: Critical D2: Moderate D3: Low D4: Low D5: Low D6: Low D7: Low D8: Serious

In this table, a detailed summary of the results from each of the included studies in this systematic review (including studies that focused only on spinal epidural abscesses) is presented. Following variables were extracted: study (author and date of publication), treatment, positive cultures (%), epidural abscess (%), duration of antibiotic treatment, additional surgical treatment required, length of hospital stay (days), relapse/failure, mortality, main conclusion of the article, and risk of bias scoring.

*PPS* percutaneous pedicle screw, *MIS-LLIF* minimally invasive spine surgery-lateral lumbar interbody fusion, *TL* thoracolumbar; the number of patients who required additional surgery after having had an initial treatment regimen or procedure is shown in the ‘Additional Surgical treatment required’ column. The number of patients who had a relapse or representation of their condition or failed their initial treatment regimen or procedure is shown in the ‘Relapse/failure’ column. The bias of each study is calculated by the ROBINS-I tool is shown in the ‘Bias’ column. *NR* data not reported, *LTFU* lost to follow up, *SEA* spontaneous epidural abscess, *MRSA* methicillin resistant *Staphylococcus aureus*, *IV* intravenous, *MRI* magnetic resonance imaging scan, *D1* bias due to confounding, *D2* bias due to selection of participants, *D3* bias in classification of interventions, *D4* bias due to deviations from intended interventions, *D5* bias due to missing data, *D6* bias in measurement of outcomes, *D7* bias in selection of the reported result, *D8* overall bias, *ESR* erythrocyte sedimentation rate.

### Treatment outcomes

#### Conservative treatment

Conservative treatment mostly consisted of intravenous and/or oral antibiotics. The antibiotic regimen was not specified in most studies^13,24,26,28–35,39,41,42,45,46,50–52^. Several studies mentioned that antibiotic therapy was targeted toward an isolated organism^23,37,40,43,47,49^. However, blood or tissue culture positivity rates ranged from 24^26^ to 93%^51^ meaning that in several cases, broad-spectrum antibiotics were required. When antibiotic regimes were specified, common treatments included vancomycin^38,44,48^, beta-lactams^37,38,40,44,49^, and linezolid^44,49^ among others. Where antibiotic therapy duration was specified, the average duration ranged between 4 and 12 weeks^27,39,40^.

#### Early surgical treatment

The most common operations performed were laminectomies, debridement surgeries, and decompression surgeries. Several different approaches were used for surgery, with a posterior approach being most referenced in 17 studies^13,24,25,28,30,31,37,39,40,43,45–49,51,52^, and an anterior approach in 13 studies^24,25,28,31,37,39,40,45,46,48,50–52^. The most common indication for surgery was the presence or worsening of a neurological deficit (n = 19 studies)^13,23–27,32,35,37–40,42,44,45,47,48,50,52^, followed by failure of conservative management with antibiotics (n = 12 studies)^23–27,29,35,35,37,44,50,50^. Definitions of early surgery were heterogeneous, and a list of definitions used can be found in Supplemental Digital Content 1: Supplementary Table S7. Twenty studies did not provide information on how much time had elapsed between patient admission or diagnosis and when they had surgery^23–27,30,31,34,35,38,39,41–46,48,50,51^. Five studies reported that patients had surgery ‘immediately’ once the diagnosis was made, but did not define this time frame quantitively^29,37,40,49,52^.

#### Early surgical treatment vs conservative treatment

A graphical summary of qualitative comparative findings is shown in Fig. 3C. Ten studies stated that the clinical outcomes (non-neurological) of early surgical treatment were superior^14,27,28,32,39,40,44,48,50,52^, while six studies stated that there was no significant difference between the two modalities (Fig. 3C[a])^23,25,33,41,42,51^. No studies reported that conservative treatment had superior clinical outcomes. It is noted, however, that a range of definitions was used to determine clinical outcomes in patients including prognosis, recurrence, hospital stay, mortality rates, and lab parameters. The definition of neurological outcomes was split in two categories—the first being the presence or absence of neurological deficit^14,39,40,44,51^ and the second being a graded scale of neurological deficit based on the American Spinal Injury Association Scale (ASIA scale)^13,23,28,50^. In terms of these neurological outcomes, six studies reported that surgical treatment resulted in superior neurological outcomes^13,14,28,39,40,50^, one study reported that conservative treatment resulted in superior neurological outcomes^44^, and three studies reported that there was no significant difference between the two modalities (Fig. 3C[b])^23,42,51^. Sixteen studies stated that overall, when taking into account both neurological and clinical outcomes, early surgery yielded better outcomes^13,14,24,26,28,29,32,34,39,40,43,44,48,50–52^, while 10 studies stated that there was no difference^23,25,27,30,31,33,37,41,42,47^. No study stated that conservative treatment was superior (Fig. 3C[c]).

### Meta-analysis

#### Mortality

**Figure 4:**
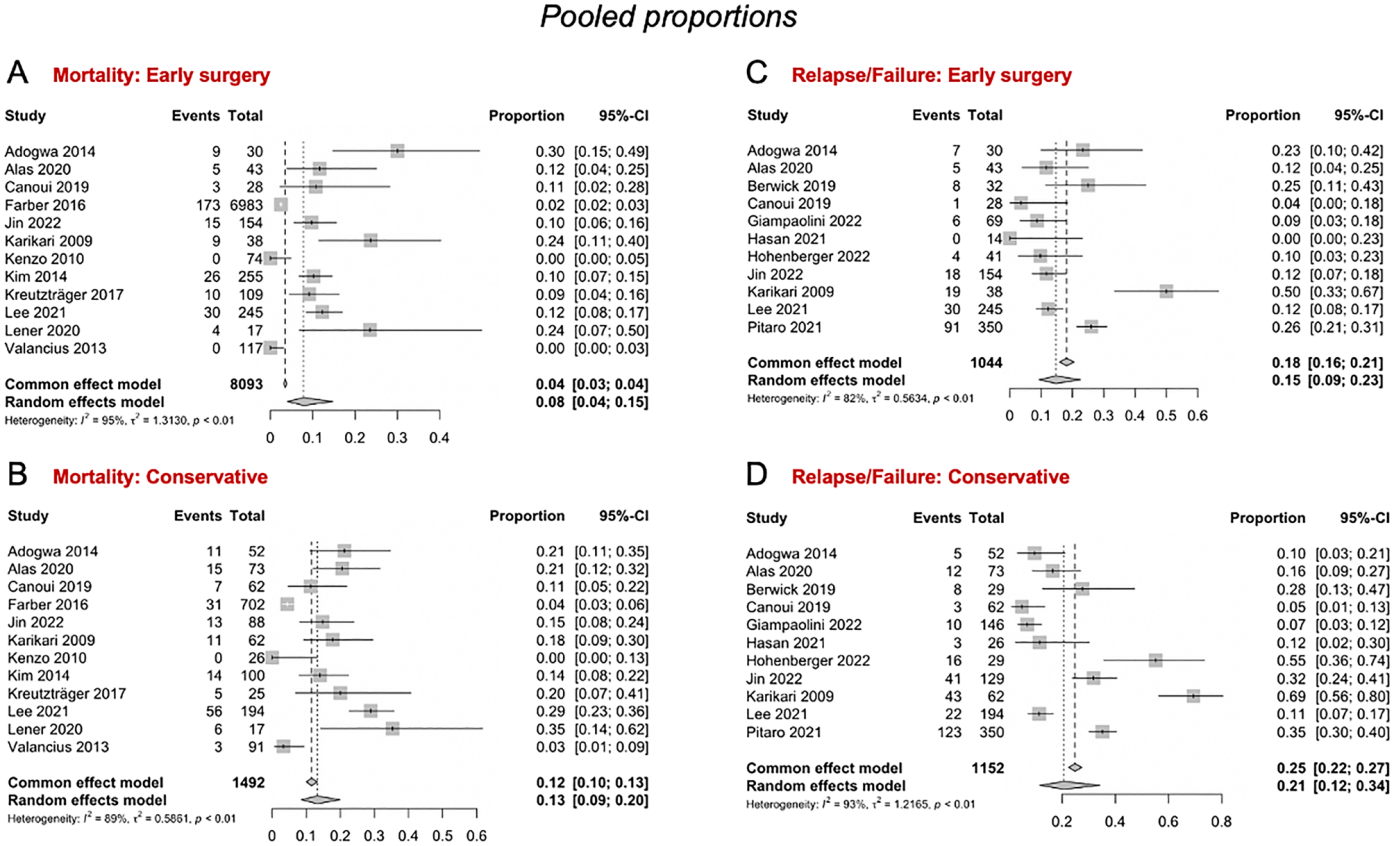
Four forest plot indicating and visualizing the proportion in mortality and relapse/failure in the context of spondylodiscitis following early surgical management (treatment arm) versus conservative management (control arm) is shown, pooling the results of all the studies included in the meta-analysis. (**A**) The pooled proportional mortality after early surgery is shown, (**B**) pooled proportional mortality after conservative treatment, (**C**) pooled proportional relapse/failure after early surgery, (**D**) pooled proportional relapse/failure after conservative treatment. The size of the grey square of the “Proportion” visual correlates to study sample size and the straight line indicated the confidence interval. The diamond at the bottom indicates the overall pooled proportion. Heterogeneity is indicated by the chi-squared statistic (*I*^2^) with associated r^2^ and p-value. The 95% confidence intervals (CI) are shown in squared bracket ([ ]). P-value < 0.05 is deemed significant. Furthermore, for every study the following are displayed: study author with publication date (“Study”), total sample size number for each study for the respective treatment arm (“Total”), number of deaths/relapses (“Events”) per respective treatment arm, and proportion of deaths/relapses (“Proportion”), test for significance of overall effect size as t_n_ and p-value, and weighting of each study in percentage (%).

For mortality, eleven studies^13,23,24,26,29,32,35,37,41–43^ (five scoring moderate risk of bias^13,37,41–43^) with a pooled sample size of n = 8,798 patients were included. The pooled proportion of mortality among patients treated with early surgery was 0.08 (CI 95% 0.04 – 0.15), or 8% (Fig. 4A), and 0.13 (CI 95% 0.09–0.20), or 13% (Fig. 4B), for patients treated conservatively.

#### Relapse/Failure

For relapse/failure, defined as the need for repeat surgery or admission after initial treatment, eleven studies^13,23–25,33–35,41,42,47,48^ (two scoring serious risk of bias^47,48^ and three scoring moderate risk^13,41,42^) were included with a pooled overall sample size of n = 2,196 of surgically and conservatively treated patients. The pooled proportion of relapse/failure among patients treated with early surgery was 0.15 (CI 95% 0.09–0.23), or 15% (Fig. 4C), and 0.21 (CI 95% 0.12–0.34), or 21%, for patients treated conservatively, in the random effects model (Fig. 4D).

#### Relative risk reduction

**Figure 5:**
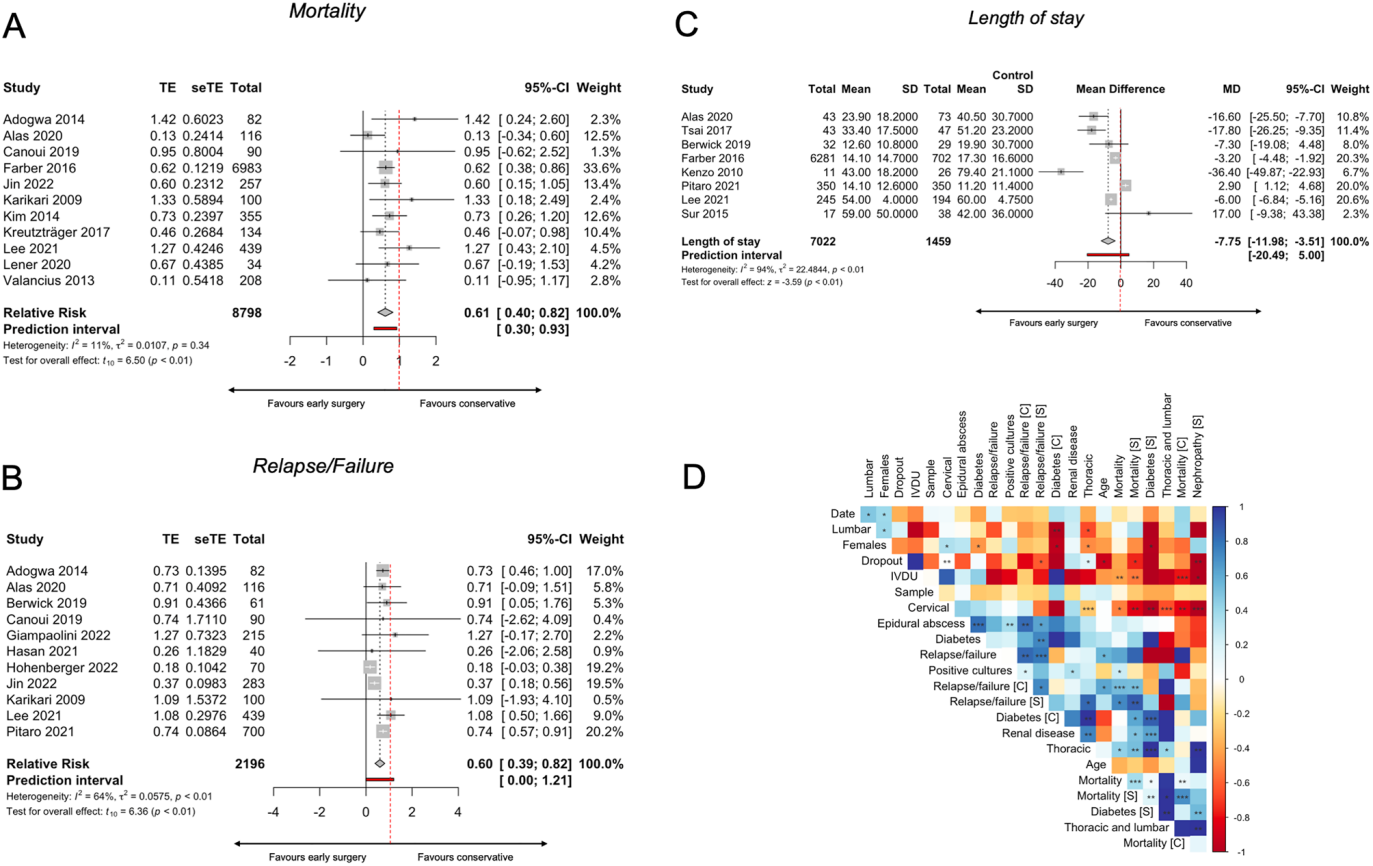
(**A**) A forest plot indicating and visualizing the treatment effect (“TE”) size in relative risk in the context of comparing the mortality rate of spondylodiscitis following early surgical management (treatment arm) versus conservative management (control arm) is shown, pooling the results of all the 11 studies included in the meta-analysis. The size of the grey square of the “Relative Risk” visual correlates to study sample size and the straight line indicated the confidence interval. The diamond at the bottom indicates the overall pooled relative risk ratio. The red bar below it indicates the prediction interval. Heterogeneity is indicated by the chi-squared statistic (*I* ^2^) with associated r^2^ and p-value. The 95% confidence intervals (CI) are shown in squared bracket ([ ]). P-value < 0.05 is deemed significant. Furthermore, for every study the following are displayed: study author with publication date (“Study”), total sample size number for each study (“Total”), and standard error of the treatment effect (“seTE”), test for significance of overall effect size as t_n_ and p-value, and weighting of each study in percentage (%). The weighting of each study represented in the percentage (%) is derived from the inverse of the variance of each study's effect estimate. This means that more weight is given to the studies that provide more detailed information or have less variability in their outcomes, giving a balanced representation of the available data in the pooled analysis. A significant pooled relative risk was yielded overall (p < 0.01), indicating that early surgical management vs conservative has a relative risk of 0.61 in the context of overall mortality. Effectively this means that early surgical management of spondylodiscitis achieves a 39% risk reduction (overall mortality) when compared to conservative management. (**B**) A forest plot indicating and visualizing the treatment effect (“TE”) size in relative risk in the context of comparing the relapse/failure/recurrence rate of spondylodiscitis following early surgical management (treatment arm) versus conservative management (control arm) is shown, pooling the results of all the 17 studies included in the meta-analysis. The size of the grey square of the “Relative Risk” visual correlates to study sample size and the straight line indicates the confidence interval. The diamond at the bottom indicates the overall pooled relative risk ratio. The red bar below it indicates the prediction interval. Heterogeneity is indicated by the chi-squared statistic (*I*^2^) with associated r^2^ and p-value. The 95% confidence intervals (CI) are shown in squared bracket ([ ]). P-value < 0.05 is deemed significant. Furthermore, for every study the following are displayed: study author with publication date (“Study”), total sample size number for each study (“Total”), and standard error of the treatment effect (“seTE”), test for significance of overall effect size as t_n_ and p-value, and weighting of each study in percentage (%). A significant pooled relative risk was yielded overall (p < 0.01), indicating that early surgical management vs conservative has a relative risk of 0.6 in the context of leading to relapse/failure/recurrence. Effectively this means that early surgical management of spondylodiscitis achieves a 40% risk reduction (relapse/failure/recurrence) when compared to conservative management. (**C**) A forest plot indicating and visualizing the treatment effect (“TE”) size in relative risk in the context of comparing the mean length of hospital stay (in daysI of spondylodiscitis patients following early surgical management (treatment arm) versus conservative management (control arm) is shown, pooling the results of all the studies included in the meta-analysis. The size of the grey square of the “Mean Difference” visual correlates to study sample size and the straight line indicated the confidence interval. The diamond at the bottom indicates the overall pooled mean difference. The red bar below it indicates the prediction interval. Heterogeneity is indicated by the chi-squared statistic (*I*^2^) with associated r^2^ and p-value. The 95% confidence intervals (CI) are shown in squared bracket ([ ]). P-value < 0.05 is deemed significant. Furthermore, for every study the following are displayed: study author with publication date (“Study”), total sample size number for each study (“Total”), and standard error of the treatment effect (“seTE”), test for significance of overall effect size as t_n_ and p-value, and weighting of each study in percentage (%). A significant pooled mean difference was yielded overall (p < 0.01), indicating that early surgical management vs conservative has -7.75 day mean difference in the context of overall length of stay, effectively meaning that surgery is associated with a mean 7.75 day reduction in length of stay. (**D**) A correlation matrix visualizes the relationships of following parameters among all studies included in the systematic review (n = 31): The following parameters are used here: Date of publication, lumbar location of infection, proportion of females overall, dropout rate, proportion of intravenous drug users, sample size, cervical location of infection, proportion of epidural abscesses, proportions of diabetics, mean overall relapse/failure rate, proportion of positive cultures (tissues and blood), relapse/failure rate in conservatively treated patient (“Relapse failure [C]”), relapse/failure rate in surgically treated patients (“Relapse failure [S]”), proportion of diabetics in conservatively treated patients, proportion of patients with diabetes, thoracic location of infection, mean age of study population, mortality rate overall, proportion of diabetics in surgically treated patients, combined thoracic and lumbar location of infection, mean overall mortality, mean mortality in surgically treated patients, proportion of nephropathy in surgically managed patients (“Nephropathy [S]”), and mean mortality in conservatively treated patients. The legend bar at the right of the matrix explains the coloring. Red hue indicates a negative association between two parameters, and a blue hue a positive association. One asterisk (*) indicates a statistical significance of p < 0.05, two asterisks (**) indicate p < 0.01, three asterisks (***) indicate p < 0.001.

The mortality risk reduction comparing early surgery to conservative treatment was 0.61 RR (CI 95% 0.40–0.82) (p < 0.01) (Fig. 5A), indicating a 39% risk reduction when using early surgery. The pooled relative risk reduction in relapse/failure rates when comparing early surgery to conservative treatment was 0.60 RR (CI 95% 0.39–0.82) (p < 0.01) (Fig. 5B), indicating a 40% risk reduction when using early surgery over conservative treatment.

#### Length of stay

For length of stay, eight studies were included with a pooled overall sample size of n = 8,481^13,24,32–34,38,50,52^, four scoring a low risk of bias^24,32–34^, two scoring a moderate risk^13,38^, one scoring a serious risk^50^, and one study scoring a critical risk of bias^52^. The overall mean difference between early surgical management and conservative management was − 7.75 (CI 95% − 11.98 to − 3.51) (p < 0.01) (Fig. 5C), indicating that early surgical management of spondylodiscitis achieves a length of stay reduction of − 7.75 days per patient when compared to conservative treatment.

#### SEA-only and SEA-excluded analyses

Six additional subgroup meta-analyses were run, two on mortality, two on relapse/failure, and two on length of stay: for each outcome variable, a meta-analysis was computed including only studies that focus solely on patients with spinal epidural abscesses (SEA); and then a meta-analysis was computed excluding the studies that focus solely on patients with SEA (Supplemental Digital Content 1: Supplementary Fig. S1A–F). The meta-analysis on relapse/failure including studies that only focussed on patients with SEA yielded 0.74 RR (CI 95% 0.68–0.80) (p < 0.01), for mortality 0.56 RR (CI 95% 0.22–0.89) (p < 0.01), for length of stay a mean difference of − 6.53 (CI 95% − 13.13 to 0.08) (p = 0.05). The meta-analysis on relapse/failure excluding studies that only focus on patients with SEA yielded 0.46 RR (CI 95% 0.12–0.80) (p = 0.02), for mortality 0.67 RR (CI 95% 0.24–1.10), with t = 6.70 (p = 0.02), for length of stay a mean difference of − 6.53 (CI 95% − 13.13 to 0.08) (p = 0.05).

#### Influence analysis and linear regression

The exclusion of outlier studies based on a set of three influence analyses (Supplemental Digital Content 1: Supplementary Figs. S2, S3, S4), did not yield a significant change in effect size (Supplemental Digital Content 1: Supplementary Figs. S5, S6, S7). The exclusion of outlier studies based on high levels of risk of bias scoring did not yield any significant changes to effect size of any of the outcome variables (Supplemental Digital Content 1: Supplementary Figs. S8, S9). The meta-regressions scored the influence of all co-variates on the overall effect size of the relapse/failure meta-analysis, mortality meta-analysis, and length of stay meta-analysis (Table 4). Only for the relapse/failure meta-analysis there were significant (p < 0.05) co-variates that were found: “IVDU” and “diabetes”. None of the exclusion subgroup meta-analyses (excluding studies with high proportions of diabetics, and the studies with high proportions of intravenous drug users) yielded strong differences in the meta-analysis effect size (Supplemental Digital Content 1: Supplementary Figs. S10, S11).

**Table 4:** Mixed-effects single-variate meta-regression.

	Relapse/failure	Mortality	Length of stay
~ Covariates			
~ Sample size	0.0004 (0.0004)	0.0000 (0.0000)	− 0.0012 (0.0018)
~ Study type	0.3530 (cohort) (1.0381)	− 0.1294 (0.3061)	24.9786 (24.3461)
~ Study design	− 0.6658 (retrospective) (0.6336)	0.1521 (retrospective) (0.3177)	NA
~ Age [S]	0.0015 (0.0334)	− 0.0069 (0.1456)	2.6759 (1.7955)
~ Age [C]	0.0019 (0.0228)	− 0.0163 (0.0612)	0.9864 (1.0817)
~ Females [S]	0.0056 (0.0127)	0.0114 (0.0307)	0.1521 (0.4568)
~ Females [C]	0.0009 (0.0054)	0.0388 (0.0209)	0.4324 (0.5072)
~ Females	0.0132 (0.0111)	0.0222 (0.0437)	7.7313 (2.4611)
~ Age	0.0049 (0.0252)	− 0.0204 (0.0382)	1.0235 (3.1723)
~ Diabetes	0.0293 * [p = 0.0426] (0.0100)	0.0519 (0.0351)	0.3603 (0.6885)
~ IVDU	− 0.0175 ** [p = 0.0077] (0.0015)	− 0.0139 (0.0536)	NA
~ Nephropathy	0.0200 (0.0075)	0.0300 (0.0475)	NA
~ Epidural abscess	− 0.3347 (0.1686)	1.1587 (1.2233)	− 13.7690 (10.6932)
~ Date of publication	− 0.0326 (0.0310)	− 0.0014 (0.0368)	− 0.1823 (1.0143)
~ Cervical	NA	0.0338 (0.0580)	NA
~ Thoracic	NA	0.0446 (0.0273)	NA
~ Lumbar	NA	0.0021 (0.0189)	NA

The results of the meta-regression of the meta-analyses for relapse/failure and mortality in spondylodiscitis management (surgical versus conservative) are presented in this table. A meta-regression analysis was run for each of the covariates (Sample size, study type, study design, age of surgically treated patients, age of conservatively treated patients, proportion of females of surgically treated patients, proportion of females of conservatively treated patients, proportion of intravenous drugs users (“IVDU”), proportion of patients with nephropathy, with epidural abscess, date of publication, infection localised in cervical, thoracic or lumbar spine) as independent variable to the dependent variable relative risk. In round brackets is the standard error. If significance is yielded (denoted with * and bold regression coefficient), the p-value of the regression coefficient is shown in squared bracket only if significant, otherwise assume non-significance. Significance is assumed for p < 0.05. If a covariate was covered by < 4 studies for a respective relapse or mortality, then a regression analysis was omitted (“–”) for this respective relationship due to insufficient data for strong regression analysis, the respective cells are marked as NA (“not applicable). The different explanatory variables were calculated singularly as sole covariates in separate meta-regression.

#### Multivariate correlation analysis

In Fig. 5D, a multivariate correlation matrix visualises and compares the occurrence of all numerical study characteristics and patient characteristics, extracted from all studies included in the systematic review (n = 31). It confirmed the influence of IVDU (positive prognostic factor in surgically managed patients), and diabetes (negative prognostic factor). An important positive prognostic factor was found to be a cervical localisation of infection (p < 0.01). Important negative prognostic factors were found to be: thoracic and/or lumbar location of infection (p < 0.001), positive cultures (tissues and blood) (p < 0.01), presence of epidural abscesses (p < 0.05), and advanced age (p < 0.05). A list of all correlations can be found in Supplemental Digital Content 1: Supplementary File S2.

## Discussion

This is the first meta-analysis, to compare early surgical versus conservative management for spondylodiscitis. The meta-analysis included 21 studies, comprising data from 10,954 patients. The findings showed that early surgery had lower mortality rates (8% vs. 13% for conservative treatment) and lower relapse/failure rates (15% vs. 21%). Early surgery also led to a shorter hospital stay of 7.75 days per patient. These results consistently favoured early surgical management for pyogenic spondylodiscitis.

Surgical debridement is a widely accepted therapy for the treatment of infectious diseases, to reduce the infection load and facilitate faster infection control, while also providing tissue samples that may help to optimise adjunct antibiotic therapy^53–55^. Generally, surgery is most effective for infection poorly penetrated by antibiotics, as well as locally contained infections such as abscesses^56–58^. However, interestingly, our meta-analysis found that while early surgery was more effective than conservative therapy for patients with purely SEA, early surgery was even more effective in spondylodiscitis (without SEA) (10.06 day versus 6.5 length of stay reduction, 44% reduction in mortality versus 33%; 54% reduction in relapse rate compared to 26%).

This finding instigates a question: Could the mechanism by which surgery achieves better outcomes for spondylodiscitis patients involve more than just debridement? One hypothesis suggests spinal stabilization achieved by surgical intervention may more substantial contributing factor^59–62^. Even though antibiotics are essential in treating the infection, they are unable to provide spinal stability^59–65^. Infection may lead to spinal macro-instability, predisposing patients to experience more pain, decreased postural control, and a decreased arc of movement. However, we recognize the existing evidence base may not be robust enough to draw definitive conclusions about the mechanism and invite further studies to explore this hypothesis.

So how should this study inform clinical practice? Whilst we undertook an exhaustive search, enabling the largest pooled analysis of its kind, alongside multiple robust approaches to managing data heterogeneity, ultimately the source evidence was largely retrospective and/or cohort by design, suffered heterogeneity with outcome reporting and definition, and held moderate risk of bias. Furthermore, the included studies largely did not report on the use of intra-operative, localised antibiotics, which have shown promising results in recent studies, hence it was not possible to perform a sensitivity analysis on this parameter^66^. Despite the seemingly promising outcomes associated with early surgery, we recognize and emphasize the limitations inherent in our study. The primary studies included in our meta-analysis were largely retrospective and cohort by design, harbouring a moderate risk of bias and outcome reporting heterogeneity. Also, it is crucial to account for the probable selection bias in these studies, where the healthiest patients were more likely to be selected for early surgery. This selection bias may partially explain the observed lower mortality and relapse rates in the early surgery group. Moreover, apart from differences in patient health, disease severity may also influence the choice and timing of treatment, as well as outcomes. However, most studies did not provide data on disease severity. Potentially, the surgical approach may act as a proxy marker of disease severity, however, the data on surgical approaches were too heterogeneous to be compared. Future studies reporting on disease severity, as well as using consensus-based and comparable operative protocols, will be required to allow for robust sensitivity analyses. Furthermore, there was a statistical suggestion of publication bias, albeit extensive subgroup analysis did not identify specific outlying studies or factors. Considering these limitations, the absolute changes in outcome thresholds in a population with probable selection bias, where relapse/failure of early surgery is still high (15% versus 21%), remain difficult to interpret. No study considered the health economics of early surgery, and superficially saving eight hospital bed days may not be a sufficient trade-off for the costs and risks of routine surgery. When considering the reconfiguration of services to enable early surgery would be substantial (as spinal surgery is a tertiary specialty), it is clear that there remain significant knowledge translation gaps. The most striking finding may be the lack of any randomised comparison. This is for three reasons: firstly, the strong rationale and current evidence, secondly, the significant and increasing burden of spondylodiscitis disease, and finally, the evidence of field-wide equipoise, a premise for any randomised comparison.

However, it is important to acknowledge the obstacles to enabling a randomised control trial on spondylodiscitis management. Firstly, there is no clear consensus on what constitutes early surgery or conservative therapy, and perhaps most importantly what constitutes spondylodiscitis (particularly in the context of SEAs). The principal outcome measures or success criteria also remain undefined. Secondly, whilst there may be clinical equipoise at a field-wide level, this does not necessarily translate into institutional or physician-level equipoise—future efforts must be made to reduce local deviations from field-level recommendations of practice, including increased communications of the latest findings to raise awareness. Finally, the relative infrequency of spondylodiscitis, the population, and treatment heterogeneity, coupled with the discrimination of outcome measures for pain or neurological function, suggest any trial would require a large, probably multi-national collaboration. This will be an immense logistical challenge and will require a sufficient clinical buy-in and research funding. Despite these challenges, given the uncertainty of the clinical approach for spondylodiscitis, combined with variations in definitions and a lack of a uniformed ICD-10 for spondylodiscitis, the authors believe that these deficiencies demand for clinical equipoise to enable randomised comparison, as well as the need for expert consensus on treatment and pathology definitions in order to provide the best care for spondylodiscitis patients.

## Conclusion

This meta-analysis, with an overall pooled sample size of 10,954 patients, suggests that early surgical management may be more effective than conservative therapy for spondylodiscitis, and is associated with a 40% risk reduction in relapse/failure, a 39% risk reduction in mortality and a 7.75 days per patient reduction in length of hospital stay (p < 0.01). Excluding SEAs, these benefits were magnified. However, given the modest quality of the source evidence, probable selection bias, and remaining unanswered questions critical for implementation, we recommend treating these findings with cautious optimism. Recognising the increasing burden of the disease and the existing limitations of current research, there is a clear call for a well-designed, multi-national randomised controlled trial.

### Supplementary Information

Supplementary Information.

## Data Availability

All relevant data supporting the findings of this study can be accessed within the Supplementary Digital Content attached to the article. Additionally, a comprehensive dataset used for the meta-analysis is freely available and can be retrieved from the public GitHub repository. To ensure transparency and replicability of the research, the repository includes both raw data and processed data utilized in the study. Please visit the following link for access: https://github.com/santhoshgthava/SpondylodiscitisMA. We strongly encourage researchers and interested parties to utilize these resources in their own investigations and analyses.

## Abbreviations

ASIA: American spinal injury association
CI: Confidence intervals
CSF: Cerebrospinal fluid
CT: Computer tomography
EMBASE: Excerpta medica dataBASE
GRADE: Grading of recommendations, assessment, development and evaluations
IQR: Interquartile range
MRI: Magnetic resonance imaging
MRSA: Methicillin-resistant Staphylococcus aureus
MSSA: Methicillin-sensitive Staphylococcus aureus
NR: Not reported
OCEBM: Oxford centre of evidence-based medicine
PRISMA: Preferred reporting items for systematic reviews and meta-analyses
PROSPERO: International prospective register of systematic reviews
ROBINS-I tool: Risk of bias in non-randomized studies-of interventions
SD: Standard deviation
SEA: Spinal epidural abscess
seTE: Standard error of the treatment effect
TE: Treatment effect
