# Supplementary material for: Conservative versus early surgical treatment in the management of pyogenic spondylodiscitis: a systematic review and meta-analysis
Source: Sci Rep. 2023 Sep 20;13:15647. doi: 10.1038/s41598-023-41381-1 (PMC10511402; doi:10.1038/s41598-023-41381-1)
Supplement: Supplementary file 1 — Supplementary Information. [file 41598_2023_41381_MOESM1_ESM.docx]

**Supplemental Digital Content 1**

**Conservative versus early surgical treatment**

**in the management of pyogenic spondylodiscitis:**

**a** **systematic review & meta-analysis.**

Santhosh G. Thavarajasingam, BSc MBBS^1,2,3,9^

Kalyan V. Vemulapalli, BSc MBBS^1.3^ ; Sajeenth Vishnu K., BSc MBBS ^1,3^

Hariharan Subbiah Ponniah, BSc^1,3^ ; Alexander Sanchez-Maroto Vogel^3,4^

Robert Vardanyan BSc MBBS^1,2.3^ ; Jonathan Neuhoff, MD^5,9^ ; Andreas Kramer, MD^6,9^

Ehab Shiban, MD PhD^7,9^ ; Florian Ringel, MD PhD^6,9^ ; Andreas K. Demetriades BSc MBBS FRCS^8,9^

Benjamin M. Davies BSc MBBS MRCS^2,9^

**INSTITUTION:**

1. Faculty of Medicine, Imperial College London, London, United Kingdom.
2. Department of Academic Neurosurgery, Addenbrooke’s Hospital, Cambridge University Hospital NHS Healthcare Trust, Cambridge, United Kingdom.
3. Imperial Brain & Spine Initiative, Imperial College London, London, United Kingdom.
4. Faculty of Medicine, Goethe-Universität Frankfurt, Frankfurt, Germany.
5. Center for Spinal Surgery and Neurotraumatology, Berufsgenossenschaftliche Unfallklinik Frankfurt am Main, Germany
6. Department of Neurosurgery, Universitätsmedizin Mainz, Mainz, Germany.
7. Department of Neurosurgery, Universitätsklinikum Augsburg, Augsburg, Germany.
8. Edinburgh Spinal Surgery Outcome Studies Group, Department of Neurosurgery, Division of Clinical Neurosciences, NHS Lothian, Edinburgh University Hospitals, Edinburgh, United Kingdom.
9. Spondylodiscitis Study Group, EANS Spine Section

Table of Contents

[Supplementary Figure 1: 5](#_Toc138678524)

[Supplementary Figure 2: 6](#_Toc138678525)

[Supplementary Figure 3 7](#_Toc138678526)

[Supplementary Figure 4 8](#_Toc138678527)

[Supplementary Figure 5 9](#_Toc138678528)

[Supplementary Figure 6 10](#_Toc138678529)

[Supplementary Figure 7 11](#_Toc138678530)

[Supplementary Figure 8 12](#_Toc138678531)

[Supplementary Figure 9 13](#_Toc138678532)

[Supplementary Figure 10 14](#_Toc138678534)

[Supplementary Figure 11 15](#_Toc138678536)

[Supplementary Table 1 16](#_Toc138678538)

[Supplementary Table 2 19](#_Toc138678539)

[Supplementary Table 3 19](#_Toc138678540)

[Supplementary Table 4 21](#_Toc138678541)

[Supplementary Table 5 23](#_Toc138678542)

[Supplementary Table 6 24](#_Toc138678543)

[Supplementary Table 7: 26](#_Toc138678544)

[Supplementary Table 8 28](#_Toc138678545)

[Supplementary File 1: 71](#_Toc138678546)

[Supplementary File 2 73](#_Toc138678549)

[References 75](#_Toc138678550)

# **Supplementary** Figure 1: Influence analysis for the relapse/failure meta-analysis.


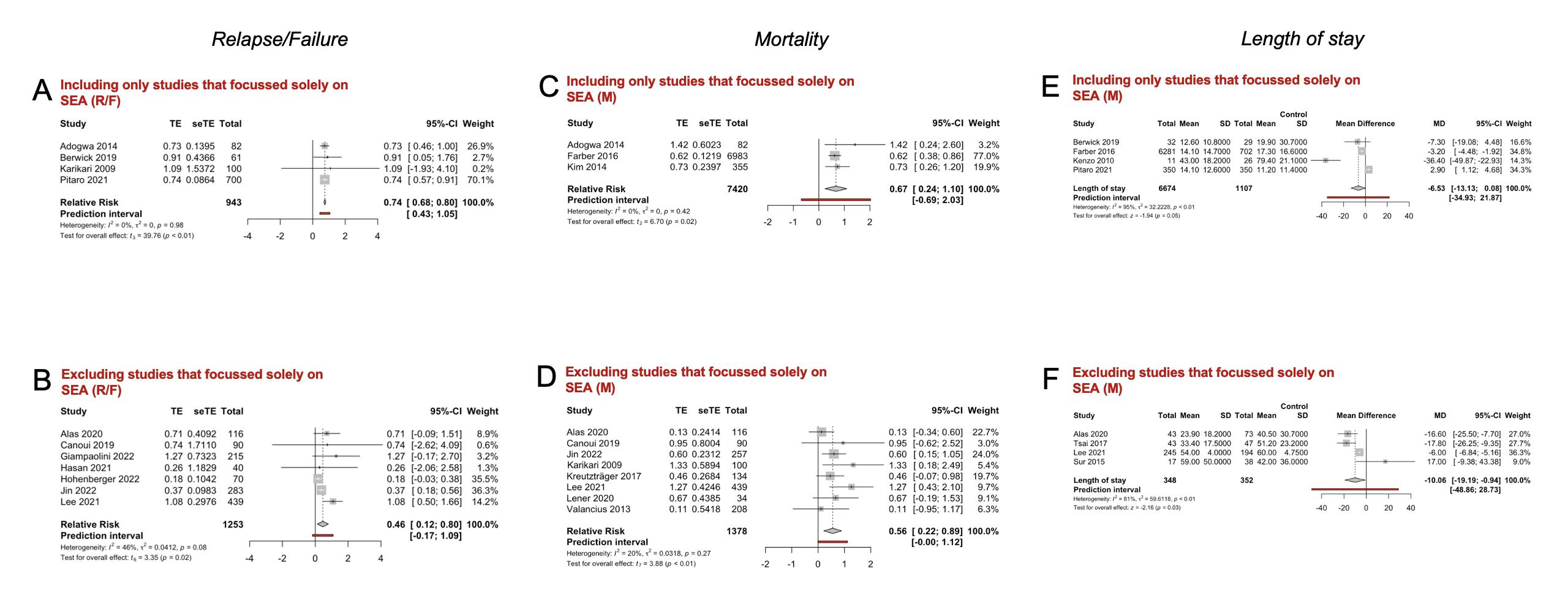


In Supplementary Figure 1A-B two forest plots indicating and visualizing the treatment effect (“TE”) size in relative risk in the context of comparing the relapse/failure following early surgery management (treatment arm) versus conservative management (control arm) for spondylodiscitis is shown, pooling the results of all the studies included in the meta-analysis, in Figure 1A focussed on studies that examined only patients with spinal epidural abscesses (SEA), in Figure 1B the same is computed but excluding studies that focussed solely on SEA. In Figure 1C-D the same is applied but for mortality as TE size, in Figure 1E-F for length of stay. The size of the grey square of the “Relative Risk” visual (mean difference for length of stay) correlates to study sample size and the straight line indicated the confidence interval. The diamond at the bottom indicates the overall pooled relative risk ratio, or pooled mean difference for length of stay. The red bar below it indicates the prediction interval. Heterogeneity is indicated by the chi-squared statistic (*I* ^2^) with associated r^2^ and p-value. The 95% confidence intervals (CI) are shown in squared bracket ([ ]). P-value < 0.05 is deemed significant. Furthermore, for every study the following are displayed: study author with publication date (“Study”), total sample size number for each study (“Total”), and standard error of the treatment effect (“seTE”), test for significance of overall effect size as t_n_ and p-value, and weighting of each study in percentage (%).

Supplementary Figure 2: Influence analysis for Relapse/Failure meta-analysis.


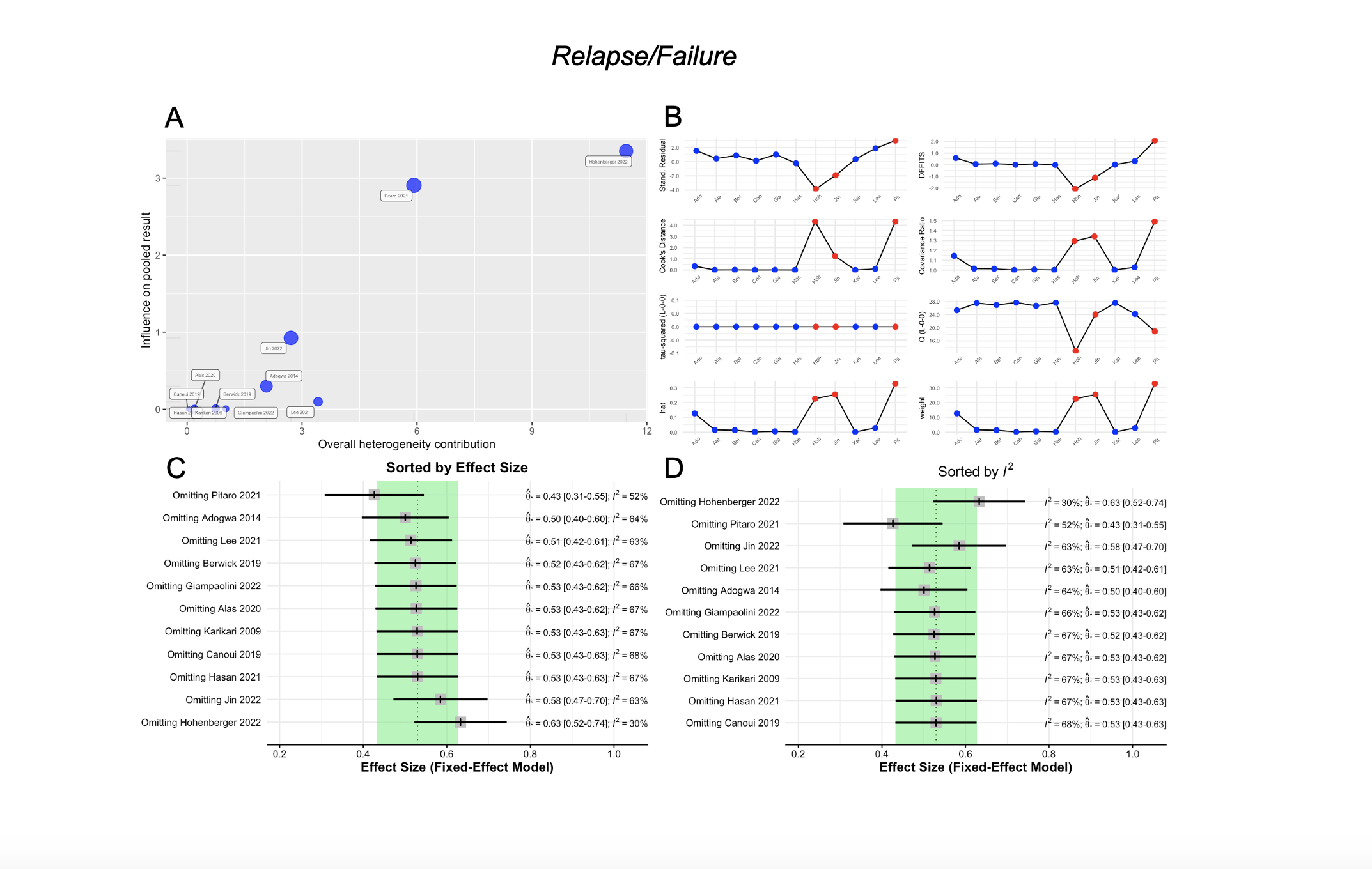


In Supplementary Figure 2 an influence analysis for the relapse/failure meta-analysis*,* is shown in four different diagnostic graphs (Supplementary Figure 2A-D). The influence analysis influence analysis aids in identifying studies that contribute highly to the between-study heterogeneity found in the meta-analysis (e.g., outliers) and could therefore be excluded in a sensitivity analysis, or have a large impact on the pooled effect size of a meta-analysis, meaning that the overall effect size may change considerably when the respective study is removed. Supplementary Figure 2A shows a Baujat plot (Baujat et al. (2002), which maps the overall heterogeneity contribution against the influence on pooled results.^1^ The studies are denoted as blue circles, with circle size corresponding to overall effect on either parameter. Supplementary Figure 2B shows an Influence Characteristics plot, which includes several influence analysis diagnostics proposed by Viechtbauer & Cheung (2010).^2^ The studies, determined to be skewing factors, using the "rules of thumb" described in Viechtbauer & Cheung (2010), are shown in red. Supplementary Figure 2C shows a forest plot for the leave-one-out analysis, sorted by effect size. The graph displays the effect size and *I^2^*-heterogeneity when omitting one of the included studies each time.^3-13^ The plot is stratified by effect size to determine which studies or effect sizes particularly affect the overall effect size, towards both extremes. The area shaded green indicates lower impact, if results fall outside, it indicates higher impact. Supplementary Figure 2D shows a forest plot for the leave-one-out analysis, sorted by *I^2^*-heterogeneity. This graph shows which studies, if excluded, result in the greatest reduction of heterogeneity.^3-13^ The area shaded green indicates lower impact, if results fall outside, it indicates higher impact.

Supplementary Figure 3**:** Influence analysis for Mortality meta-analysis.


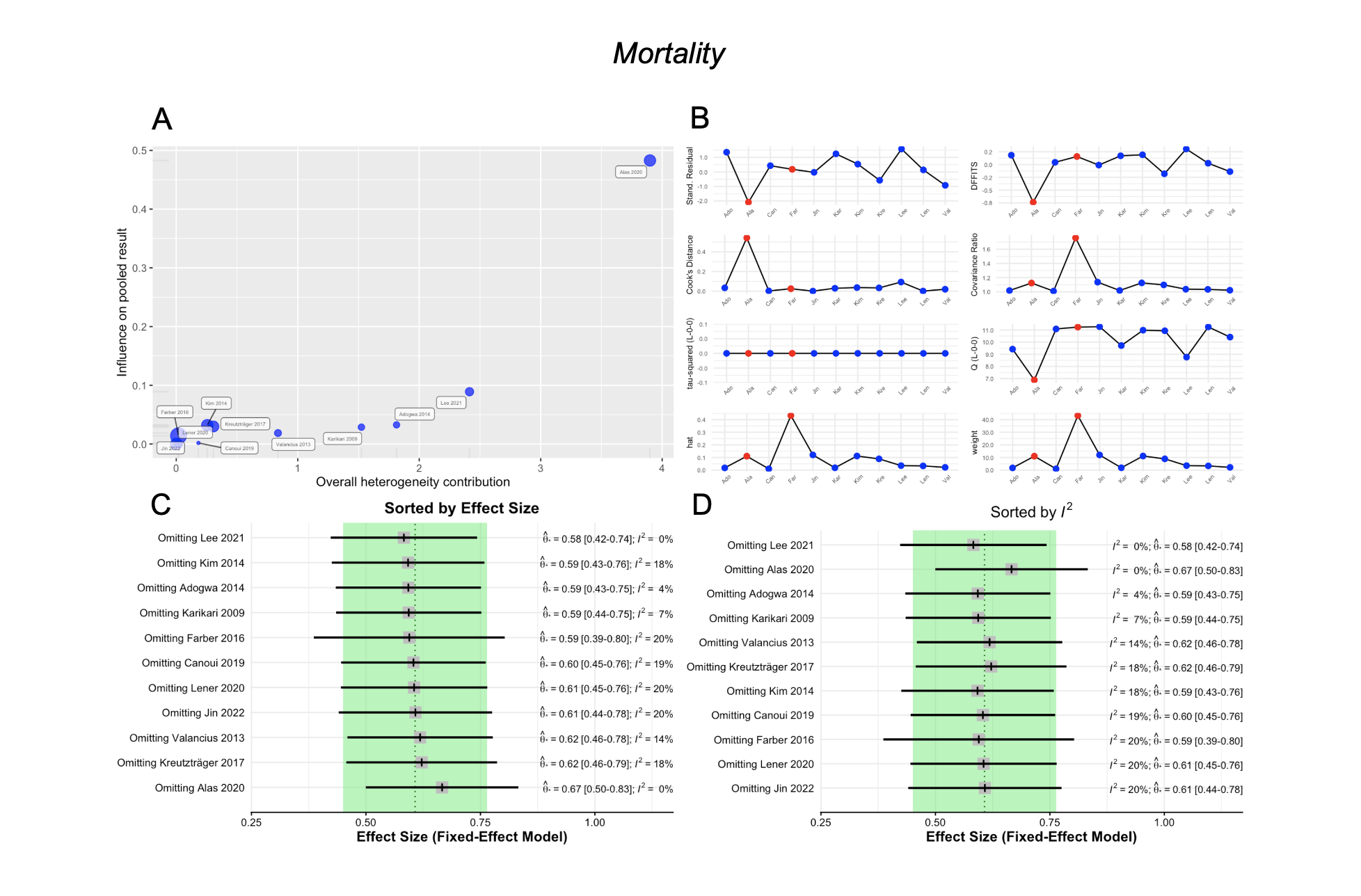


In Supplementary Figure 3 an influence analysis for the mortality meta-analysis is shown in four different diagnostic graphs (Supplementary Figure 3A-D). The influence analysis aids in identifying studies that contribute highly to the between-study heterogeneity found in the meta-analysis (e.g., outliers) and could therefore be excluded in a sensitivity analysis, or have a large impact on the pooled effect size of a meta-analysis, meaning that the overall effect size may change considerably when the respective study is removed. Supplementary Figure 3A shows a Baujat plot (Baujat et al. (2002),^1^ which maps the overall heterogeneity contribution against the influence on pooled results. The studies are denoted as blue circles, with circle size corresponding to overall effect on either parameter. Supplementary Figure 3B shows an Influence Characteristics plot, which includes several influence analysis diagnostics proposed by Viechtbauer & Cheung (2010).^2^ The studies, determined to be skewing factors, using the "rules of thumb" described in Viechtbauer & Cheung (2010), are shown in red. Supplementary Figure 3C shows a forest plot for the leave-one-out analysis, sorted by effect size.^4,5,8-10,12,14-18^ The graph displays the effect size and *I^2^*-heterogeneity when omitting one of the included studies each time. The plot is stratified by effect size to determine which studies or effect sizes particularly affect the overall effect size, towards both extremes. The area shaded green indicates lower impact, if results fall outside, it indicates higher impact. Supplementary Figure 3D shows a forest plot for the leave-one-out analysis, sorted by *I^2^*-heterogeneity. This graph shows which studies, if excluded, result in the greatest reduction of heterogeneity.^4,5,8-10,12,14-18^ The area shaded green indicates lower impact, if results fall outside, it indicates higher impact.

Supplementary Figure 4**:** Influence analysis for Length of stay meta-analysis.


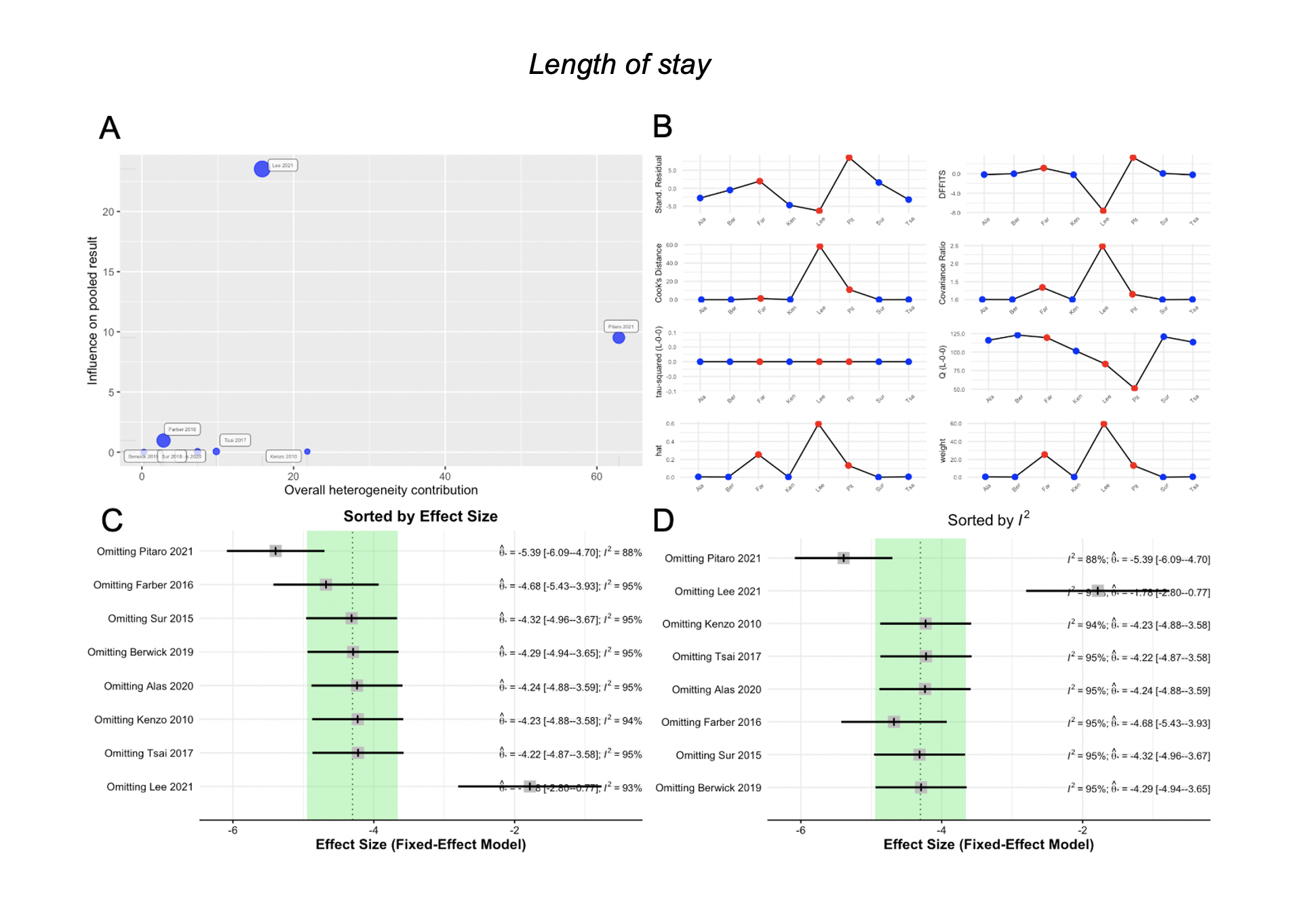


In Supplementary Figure 4 an influence analysis for the length of stay meta-analysis*,* is shown in four different diagnostic graphs (Supplementary Figure 4A-D). The influence analysis aids in identifying studies that contribute highly to the between-study heterogeneity found in the meta-analysis (e.g., outliers) and could therefore be excluded in a sensitivity analysis, or have a large impact on the pooled effect size of a meta-analysis, meaning that the overall effect size may change considerably when the respective study is removed. Supplementary Figure 4A shows a Baujat plot (Baujat et al. (2002),^1^ which maps the overall heterogeneity contribution against the influence on pooled results. The studies are denoted as blue circles, with circle size corresponding to overall effect on either parameter. Supplementary Figure 4B shows an Influence Characteristics plot, which includes several influence analysis diagnostics proposed by Viechtbauer & Cheung (2010).^2^ The studies, determined to be skewing factors, using the "rules of thumb" described in Viechtbauer & Cheung (2010), are shown in red. Supplementary Figure 4C shows a forest plot for the leave-one-out analysis, sorted by effect size. The graph displays the effect size and *I^2^*-heterogeneity when omitting one of the included studies each time.^3,5,6,8,15,19-21^ The plot is stratified by effect size to determine which studies or effect sizes particularly affect the overall effect size, towards both extremes. The area shaded green indicates lower impact, if results fall outside, it indicates higher impact. Supplementary Figure 4D shows a forest plot for the leave-one-out analysis, sorted by *I^2^*-heterogeneity. This graph shows which studies, if excluded, result in the greatest reduction of heterogeneity.^3,5,6,8,15,19-21^ The area shaded green indicates lower impact, if results fall outside, it indicates higher impact.

Supplementary Figure 5**:** Relapse/Failure meta-analysis excluding outlier studies.


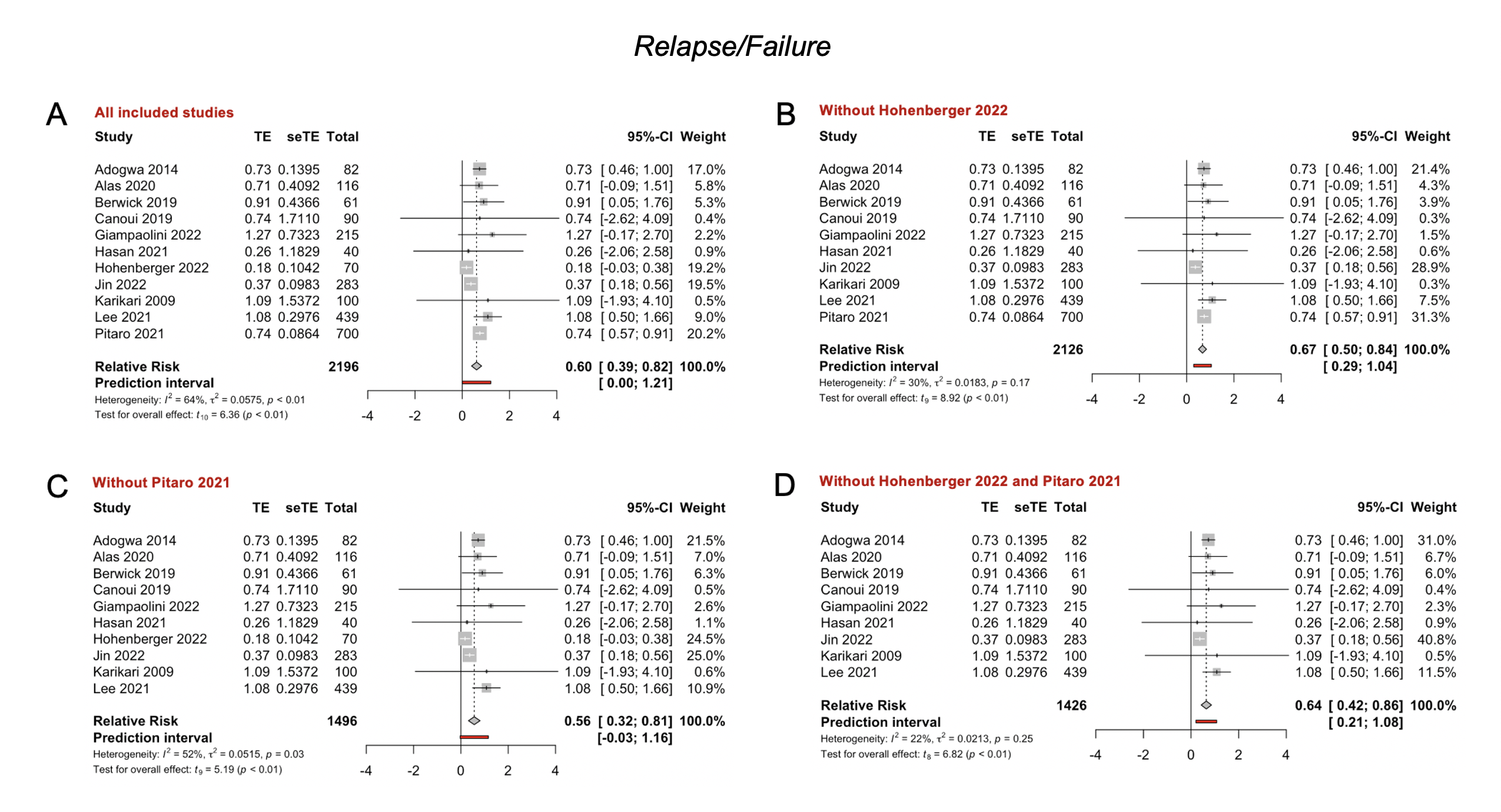


Supplementary Figure 5**:** In Supplementary Figure 5 four forest plot indicating and visualizing the treatment effect (“TE”) size in relative risk in the context of comparing the relapse/failure/recurrence rate of spondylodiscitis following early surgical management (treatment arm) versus conservative management (control arm) is shown, pooling the results of all the 17 studies included in the Relapse/failure meta-analysis in Supplementary Figure 5A,^3-13^ as well as excluding studies identified by the influence analysis as outliers, excluding Hohenberger et al. (2022) in Supplementary Figure 5B,^13^ excluding Pitaro et al. (2021) in Supplementary Figure 5C,^3^ and excluding Hohenberger et al. (2022) and Pitaro et al. (2021) in Supplementary Figure 5D.^3,13^ The size of the grey square of the “Relative Risk” visual correlates to study sample size and the straight line indicates the confidence interval. The diamond at the bottom indicates the overall pooled relative risk ratio. The red bar below it indicates the prediction interval. Heterogeneity is indicated by the chi-squared statistic (*I* ^2^) with associated r^2^ and p-value. The 95% confidence intervals (CI) are shown in squared bracket ([ ]). P-value < 0.05 is deemed significant. Furthermore, for every study the following are displayed: study author with publication date (“Study”), total sample size number for each study (“Total”), and standard error of the treatment effect (“seTE”), test for significance of overall effect size as t_n_ and p-value, and weighting of each study in percentage (%). Exclusion of outlier studies had no strong effect on effect size.

Supplementary Figure 6**:** Mortality meta-analysis excluding outlier studies.


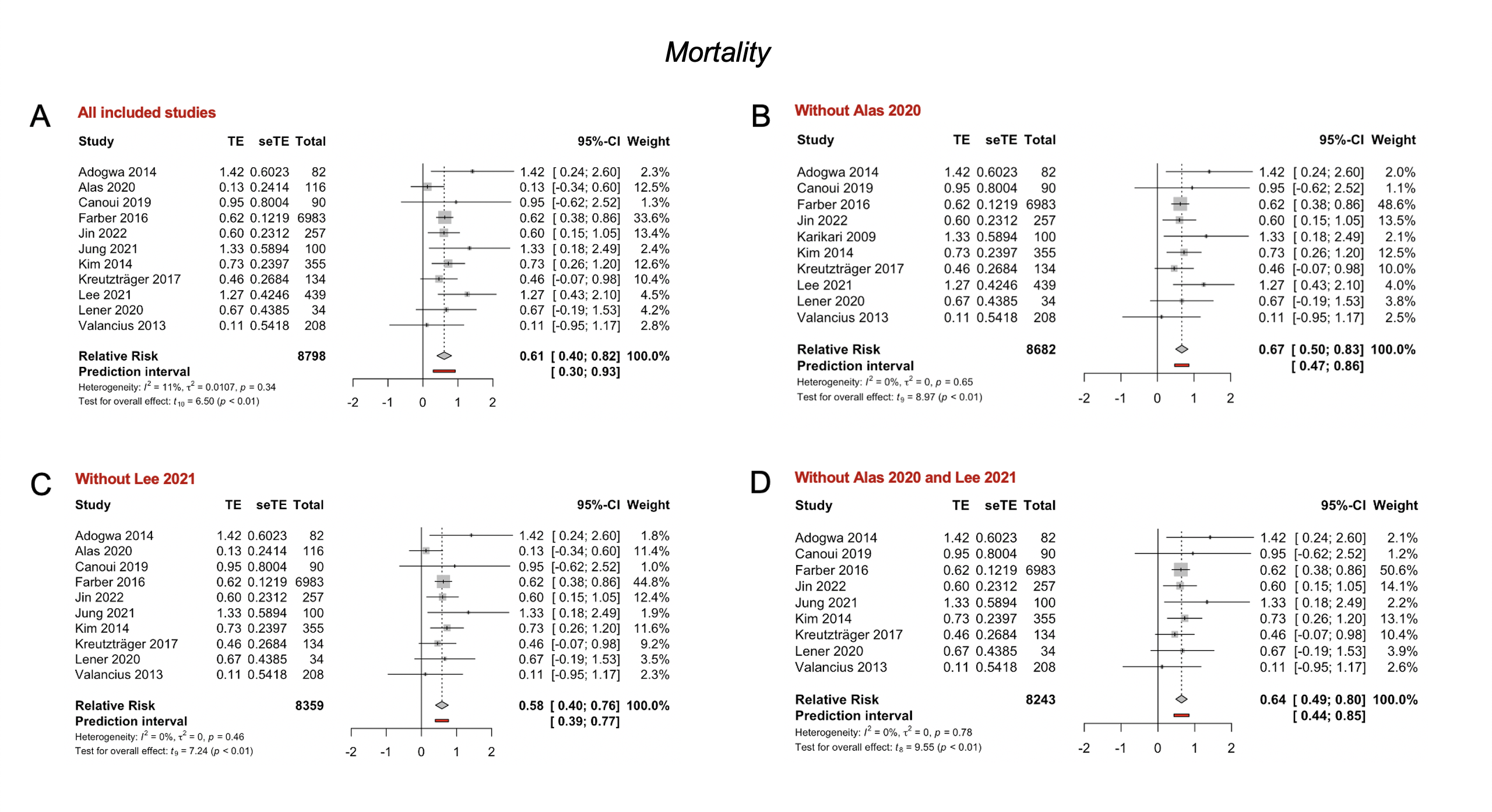


In Supplementary Figure 6, four forest plots indicating and visualizing the treatment effect (“TE”) size in relative risk in the context of comparing the mortality rate of spondylodiscitis following early surgical management (treatment arm) versus conservative management (control arm) is shown, pooling the results of all the 11 studies included in the meta-analysis in Supplementary Figure 6A, as well as excluding studies identified by the influence analysis as outliers:^4,5,8,10,12,14-18,22^ excluding Alas et al. (2020) in Supplementary Figure 6B,^8^ excluding Lee et al. (2021) in Supplementary Figure 6C,^5^ and excluding Alas et al. (2020) and Lee et al. (2021) in Supplementary Figure 6D. ^5,8^ The size of the grey square of the “Relative Risk” visual correlates to study sample size and the straight line indicated the confidence interval. The diamond at the bottom indicates the overall pooled relative risk ratio. The red bar below it indicates the prediction interval. Heterogeneity is indicated by the chi-squared statistic (*I* ^2^) with associated r^2^ and p-value. The 95% confidence intervals (CI) are shown in squared bracket ([ ]). P-value < 0.05 is deemed significant. Furthermore, for every study the following are displayed: study author with publication date (“Study”), total sample size number for each study (“Total”), and standard error of the treatment effect (“seTE”), test for significance of overall effect size as t_n_ and p-value, and weighting of each study in percentage (%). Exclusion of outlier studies had no strong effect on effect size.

Supplementary Figure 7**:** Length of stay meta-analysis excluding outlier studies.


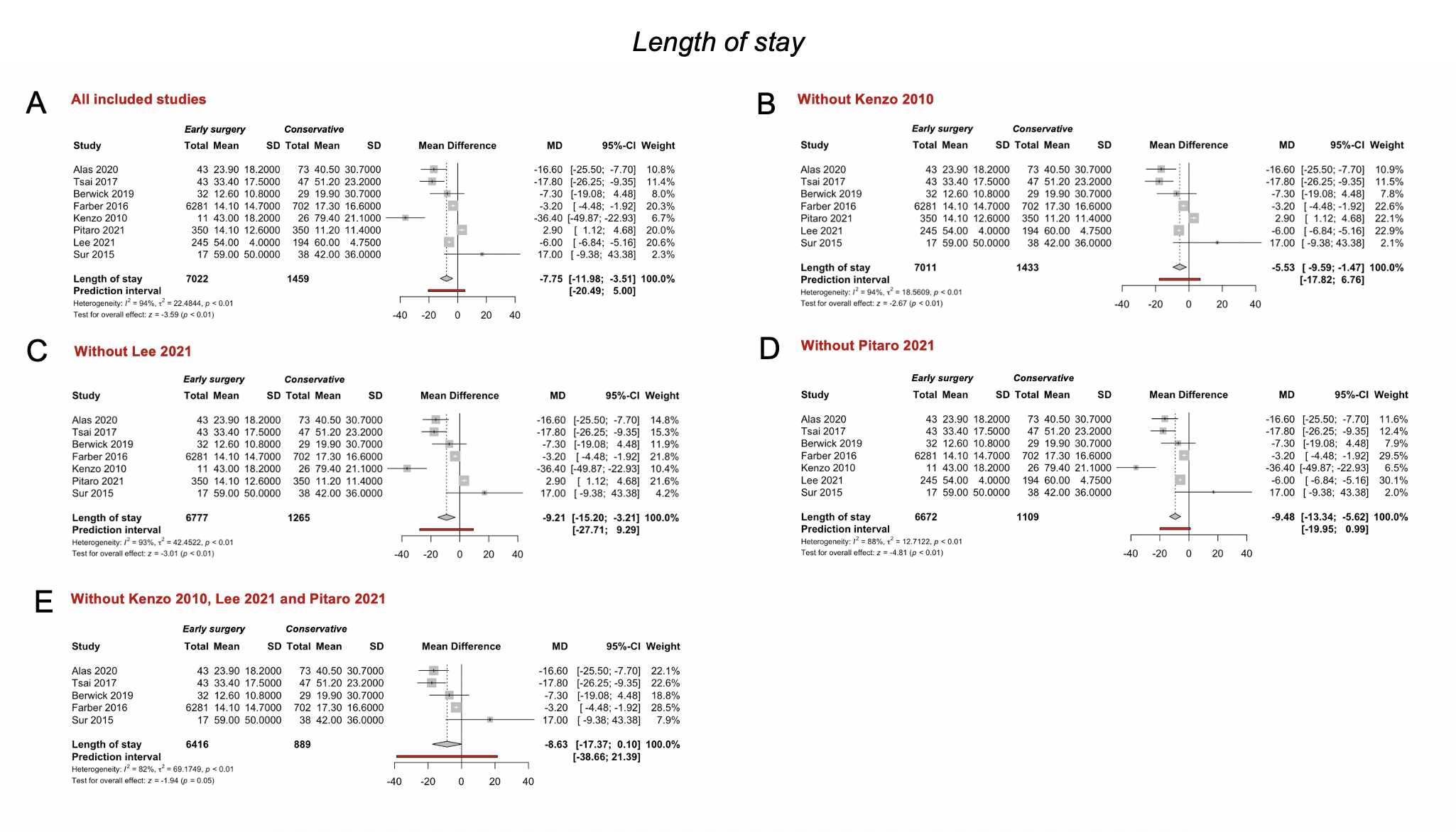


In Supplementary Figure 7 five forest plots indicating and visualizing the treatment effect (“TE”) size in relative risk in the context of comparing the mean length of hospital stay of spondylodiscitis patients following early surgical management (treatment arm) versus conservative management (control arm) is shown, pooling the results of all the studies included in the meta-analysis in Supplementary Figure 7A, as well as excluding studies identified by the influence analysis as outliers: ^3,5,6,8,15,19-21^ excluding Kenzo et al. (2010) in Supplementary Figure 7B,^20^ excluding Lee et al. (2021) in Supplementary Figure 7C,^5^ excluding Pitaro et al. (2021),^3^ and excluding all of these three in Supplementary Figure 7D.^3,5,20^ The size of the grey square of the “Mean Difference” visual correlates to study sample size and the straight line indicated the confidence interval. The diamond at the bottom indicates the overall pooled mean difference. The red bar below it indicates the prediction interval. Heterogeneity is indicated by the chi-squared statistic (*I* ^2^) with associated r^2^ and p-value. The 95% confidence intervals (CI) are shown in squared bracket ([ ]). P-value < 0.05 is deemed significant. Furthermore, for every study the following are displayed: study author with publication date (“Study”), total sample size number for each study (“Total”), and standard error of the treatment effect (“seTE”), test for significance of overall effect size as t_n_ and p-value, and weighting of each study in percentage (%).

Supplementary Figure 8: Relapse/Failure meta-analysis excluding studies that scored high risk of bias.

**
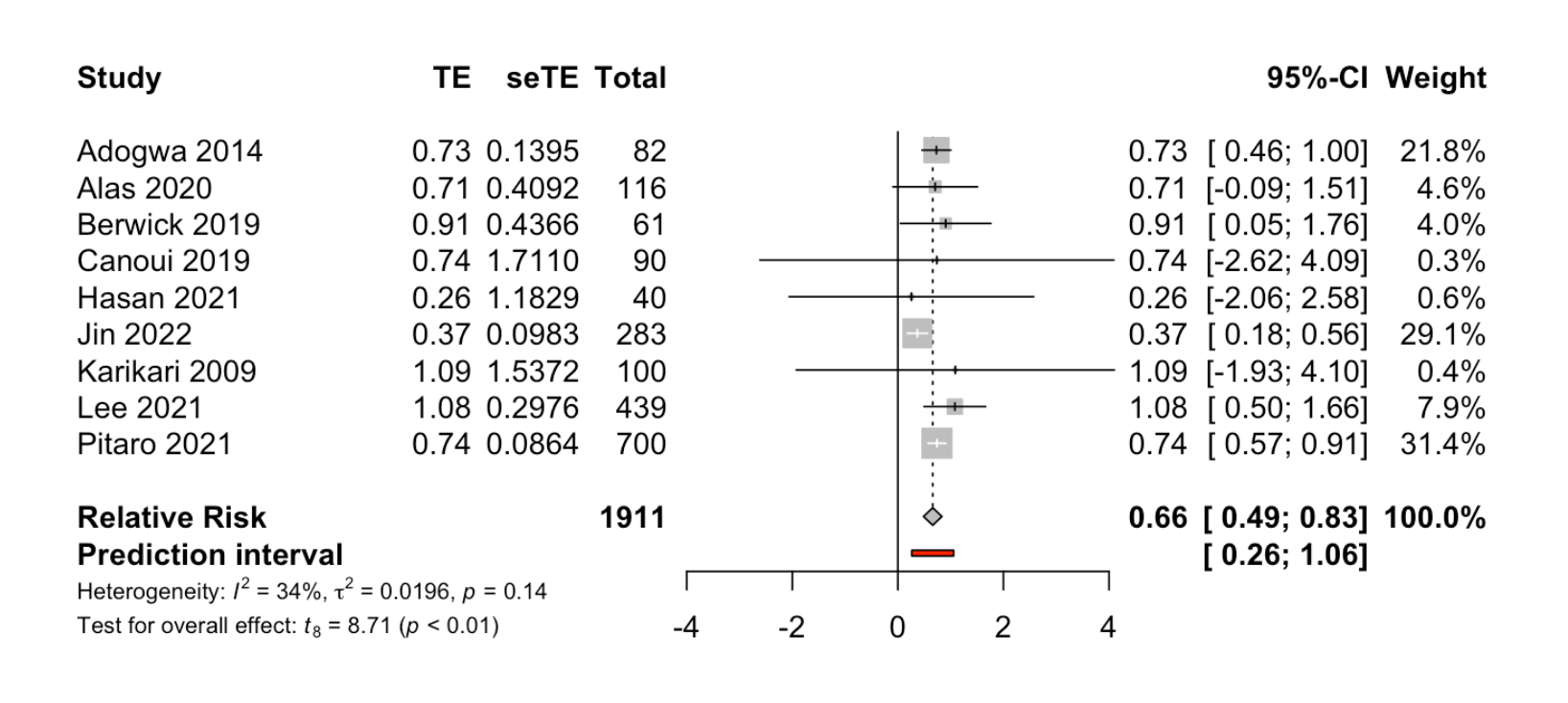
**

In Supplementary Figure 8 a forest plot indicating and visualizing the treatment effect (“TE”) size in relative risk in the context of comparing the relapse/failure rate of spondylodiscitis following operative management (treatment arm) versus conservative management (control arm) is shown, pooling the results of all the 17 studies included in the meta-analysis, excluding Hohenberger et al. (2022) and Giampaloni et al.(2020), which scored both “serious” in the risk of bias analysis.^3-6,8-12^ The size of the grey square of the “Relative Risk” visual correlates to study sample size and the straight line indicated the confidence interval. The diamond at the bottom indicates the overall pooled odds ratio. The red bar below it indicates the prediction interval. Heterogeneity is indicated by the chi-squared statistic (*I* ^2^) with associated r^2^ and p-value. The 95% confidence intervals (CI) are shown in squared bracket ([ ]). P-value < 0.05 is deemed significant. Furthermore, for every study the following are displayed: study author with publication date (“Study”), total sample size number for each study (“Total”), and standard error of the treatment effect (“seTE”), test for significance of overall effect size as tn and p-value, and weighting of each study in percentage (%). A significant pooled relative risk was yielded overall (p<0.01), indicating that operative management vs conservative has a relative risk of 0.66 in the context of leading to relapse/failure/recurrence. Effectively this means that operative management of spondylodiscitis achieves a 34% risk reduction (relapse/failure) when compared to conservative management.

Supplementary Figure 9: Length of stay meta-analysis excluding studies that scored high risk of bias.


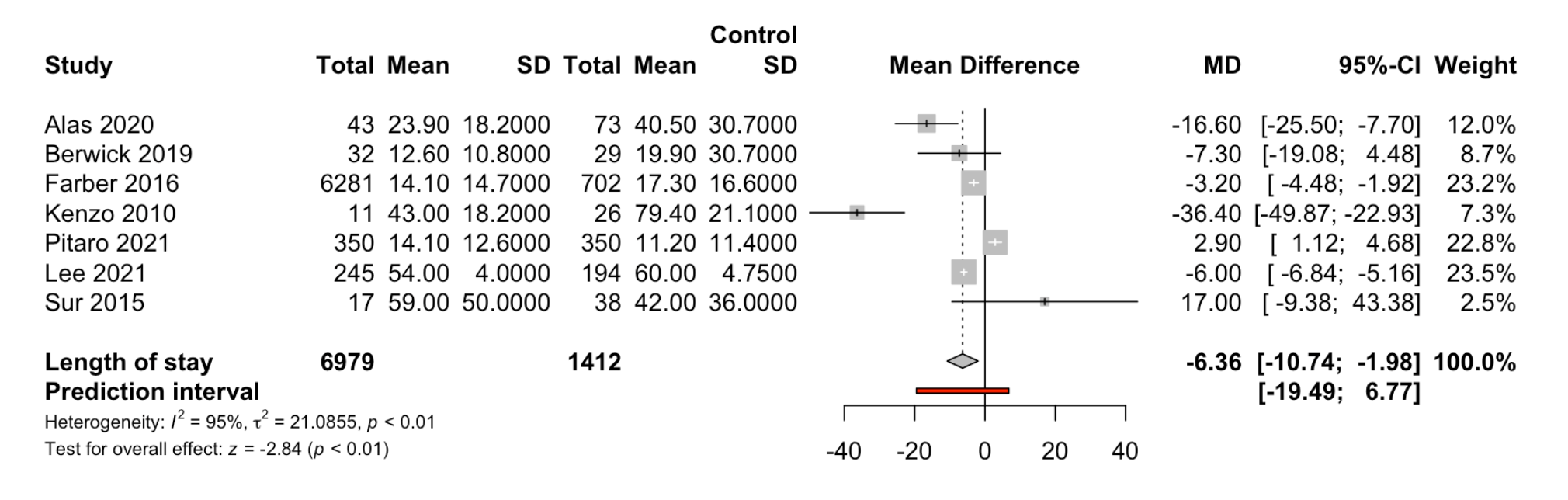


In Supplementary Figure 9 forest plots indicating and visualizing the treatment effect (“TE”) size in relative risk in the context of comparing the mean length of hospital stay of spondylodiscitis patients following surgical management (treatment arm) versus conservative management (control arm) is shown, pooling the results of all the studies included in the meta-analysis in^3,5,6,8,15,19,20^ excluding Tsai et al. (2017),^21^ that scored highly on the risk of bias analysis.^23^ The size of the grey square of the “Relative Risk” visual correlates to study sample size and the straight line indicated the confidence interval. The diamond at the bottom indicates the overall pooled odds ratio. The red bar below it indicates the prediction interval. Heterogeneity is indicated by the chi-squared statistic (*I* ^2^) with associated r^2^ and p-value. The 95% confidence intervals (CI) are shown in squared bracket ([ ]). P-value < 0.05 is deemed significant. Furthermore, for every study the following are displayed: study author with publication date (“Study”), total sample size number for each study (“Total”), and standard error of the treatment effect (“seTE”), test for significance of overall effect size as tn and p-value, and weighting of each study in percentage (%). A significant pooled relative risk was yielded overall (p<0.01), indicating that operative management vs conservative has a -6.36 days length of stay reduction. Effectively this means that operative management of spondylodiscitis achieves a 6.36 days reduction in lenth od stay when compared to conservative management.

Supplementary Figure 10: Relapse/Failure meta-analysis excluding studies that had high proportion of diabetics.


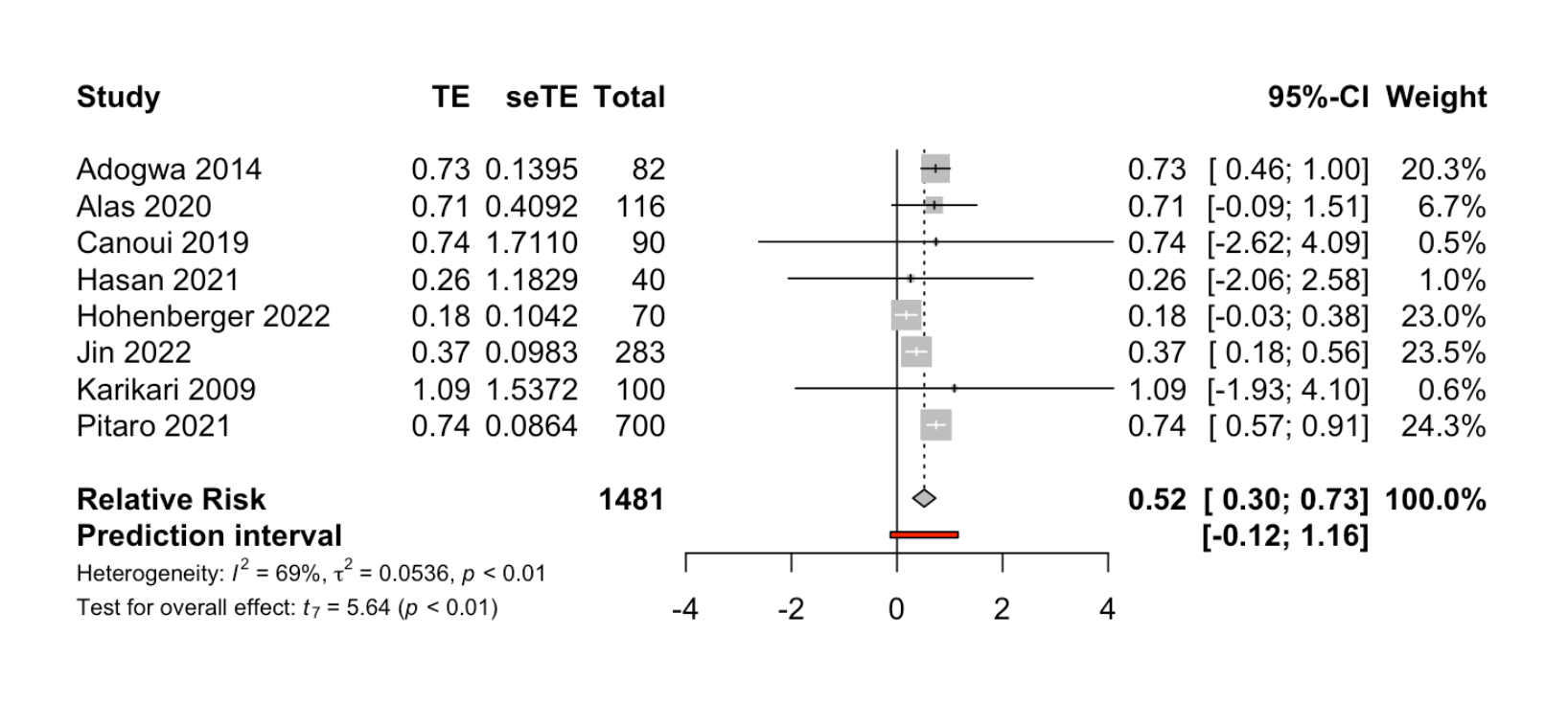


In Supplementary Figure 10 a forest plot indicating and visualizing the treatment effect (“TE”) size in relative risk in the context of comparing the relapse/failure rate of spondylodiscitis following operative management (treatment arm) versus conservative management (control arm) is shown, pooling the results of all 11 studies included in the meta-analysis,^3,4,7-13^ excluding Berwick et al. (2019) and Lee et al. (2021),^5,6^ which both had relatively high proportions of diabetics. The size of the grey square of the “Relative Risk” visual correlates to study sample size and the straight line indicated the confidence interval. The diamond at the bottom indicates the overall pooled odds ratio. The red bar below it indicates the prediction interval. Heterogeneity is indicated by the chi-squared statistic (*I* ^2^) with associated r^2^ and p-value. The 95% confidence intervals (CI) are shown in squared bracket ([ ]). P-value < 0.05 is deemed significant. Furthermore, for every study the following are displayed: study author with publication date (“Study”), total sample size number for each study (“Total”), and standard error of the treatment effect (“seTE”), test for significance of overall effect size as tn and p-value, and weighting of each study in percentage (%). A significant pooled relative risk was yielded overall (p<0.01), indicating that operative management vs conservative has a relative risk of 0.53 in the context of leading to relapse/failure/recurrence. Effectively this means that operative management of spondylodiscitis achieves a 47% risk reduction (relapse/failure) when compared to conservative management.

Supplementary Figure 11: Relapse/Failure meta-analysis excluding studies that had high proportion of IV drug users


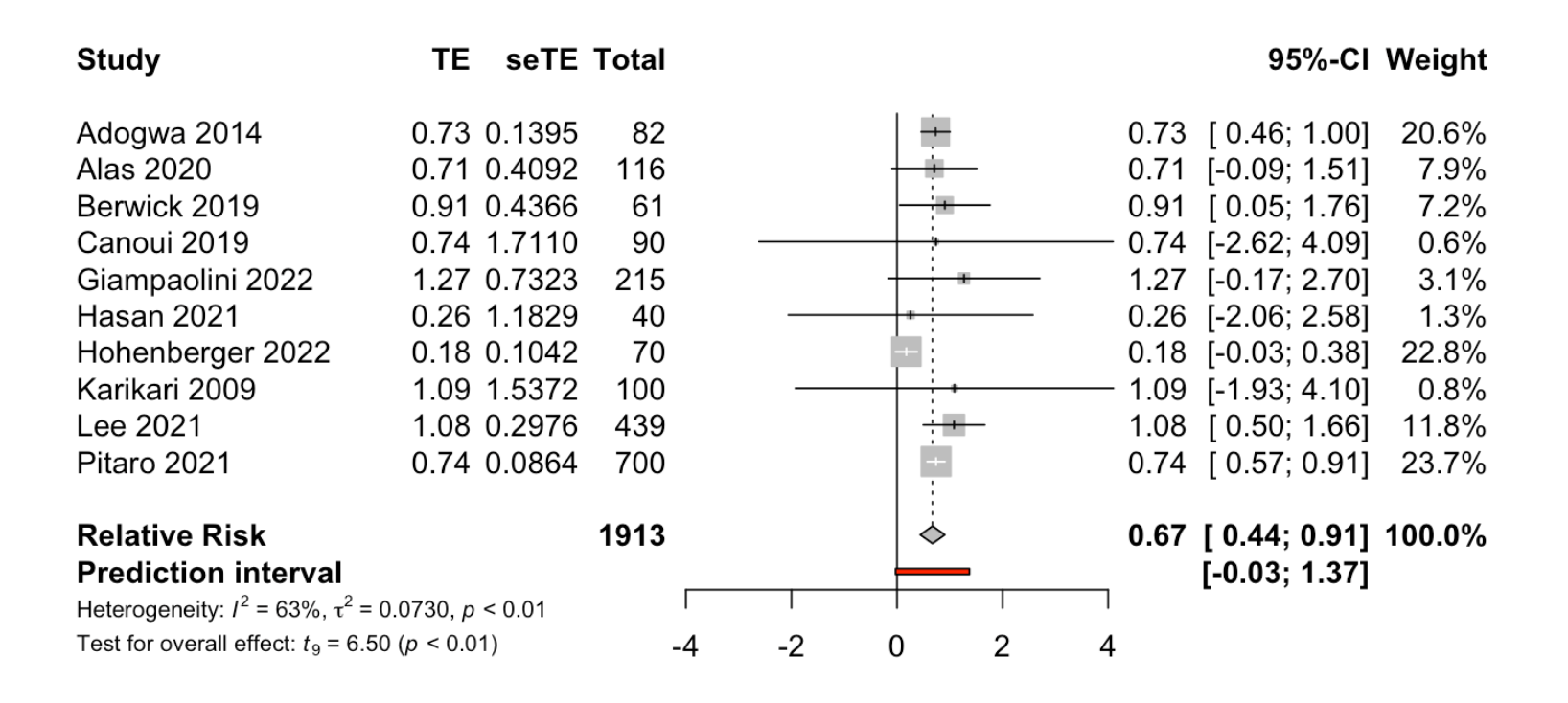


In Supplementary Figure 11 a forest plot indicating and visualizing the treatment effect (“TE”) size in relative risk in the context of comparing the relapse/failure rate of spondylodiscitis following operative management (treatment arm) versus conservative management (control arm) is shown, pooling the results of all the 11 studies included in the meta-analysis in,^3-11,13^ excluding Jin et al. (2022),^12^ the study with the highest proportion of IVDU, being a clear outlier with 22.3% of IVDU in the patient sample. The size of the grey square of the “Relative Risk” visual correlates to study sample size and the straight line indicated the confidence interval. The diamond at the bottom indicates the overall pooled odds ratio. The red bar below it indicates the prediction interval. Heterogeneity is indicated by the chi-squared statistic (*I* ^2^) with associated r^2^ and p-value. The 95% confidence intervals (CI) are shown in squared bracket ([ ]). P-value < 0.05 is deemed significant. Furthermore, for every study the following are displayed: study author with publication date (“Study”), total sample size number for each study (“Total”), and standard error of the treatment effect (“seTE”), test for significance of overall effect size as tn and p-value, and weighting of each study in percentage (%). A significant pooled relative risk was yielded overall (p<0.01), indicating that operative management vs conservative has a relative risk of 0.67 in the context of leading to relapse/failure/recurrence. Effectively this means that operative management of spondylodiscitis achieves a 43% risk reduction (relapse/failure) when compared to conservative management.

Supplementary Table 1: Search strategy.

| **Database** | **Search terms** | **Publication dates** | **Results (n)** |
| --- | --- | --- | --- |
| Medline | ((Spondylodiscitis or vertebral osteomyelitis or osteodiscitis or (discitis and spondylitis) or spinal abscess or intradural abscess or epidural abscess or intraspinal abscess or spinal infection) and (Treatment or therapy or antibiotics or surgery or treat or antibiotic or antimicrobial or conservative or debride or debridement or radical or antibacterial)).mp. [mp=ti, ab, hw, tn, ot, dm, mf, dv, kf, fx, dq, nm, ox, px, rx, ui, sy] | 1946 - 2022 | n = 8,359 |
| Embase | ((Spondylodiscitis or vertebral osteomyelitis or osteodiscitis or (discitis and spondylitis) or spinal abscess or intradural abscess or epidural abscess or intraspinal abscess or spinal infection) and (Treatment or therapy or antibiotics or surgery or treat or antibiotic or antimicrobial or conservative or debride or debridement or radical or antibacterial)).mp. [mp=ti, ab, hw, tn, ot, dm, mf, dv, kf, fx, dq, nm, ox, px, rx, ui, sy] | 1947 - 2022 | n = 4,870 |
| Scopus | ( ( spondylodiscitis OR vertebral AND osteomyelitis OR osteodiscitis OR ( discitis AND spondylitis ) OR spinal AND abscess OR intradural AND abscess OR epidural AND abscess OR intraspinal AND abscess OR spinal AND infection ) AND ( treatment OR therapy OR antibiotics OR surgery OR treat OR antibiotic OR antimicrobial OR conservative OR debride OR debridement OR radical OR antibacterial ) ) | 1943 - 2022 | n = 6,190 |
| Pubmed | ((Spondylodiscitis OR vertebral osteomyelitis OR osteodiscitis OR (discitis AND spondylitis) OR spinal abscess OR intradural abscess OR epidural abscess OR intraspinal abscess and (Treatment OR therapy OR antibiotics OR surgery OR treat OR antibiotic OR antimicrobial OR conservative OR debride OR debridement OR radical OR antibacterial)) | 1943 - 2022 | n = 8,724 |
| JSTOR | spondylodiscitis | 1943 - 2022 | n = 67 |

In Supplementary Table 1 the search strategy performed on 1^st^ November 2021 is shown below outlining the respective databases, the search terms, publication dates chosen as limiting factors, and number of results from each database are shown.

Supplementary Table 2**:** Inclusion and exclusion criteria.

| **Inclusion criteria** | **Exclusion criteria** |
| --- | --- |
| - Published in the English language - Peer-reviewed journals - Adult human patients with spondylodiscitis (see search string for synonyms) - Studies comparing conservative (antibiotics) to surgical treatment (+/- antibiotics, +/- delayed surgery) - For meta-analysis: Comparison of outcomes in patients treated with conservative versus early surgery surgery, with comparable outcome measures | - All non-English languages - Commentaries, case reports, narrative reviews, letters to editors, books - Any animal studies and lab-based studies - Studies on children and adolescents (<18 years) - Patient with previous/recent spinal instrumentation |

In Supplementary Table 2, the inclusion and exclusion criteria used when filtering studies based off search results are shown.

Supplementary Table 3**:** Table of extracted variables.

| **Extracted variables for qualitative synthesis** | **Extracted variables for quantitative synthesis** |
| --- | --- |
| - Study, Sample size, Study type and design, Country, Antibiotic used, Surgery used, Surgical Approach, Indication for surgery, Definition of Relapse/Failure, Definition of Mortality, Dropout / Patients Lost To Follow Up (LTFU), Complications, Treatment, Positive cultures, Epidural Abscess, Duration of antibiotic treatment, Additional surgical treatment required, Length of hospital stay (days), Relapse/failure, Mortality, Main conclusion, Risk of bias. | - Relapse/Failure & Mortality RR Meta-analyses each: Author, TE, seTE, Sample, lower, upper, pval, Sample size, Study type, Study design, Age [S], Females [S], Age [C], Females [C], Age, Females, Diabetes, IVDU, Nephropathy, Epidural abscess, Date. - Relapse/Failure & Mortality Total Meta-analyses each: Author, TE, seTE, Sample, lower, upper, pval, Sample size, Study type, Study design, Age [S], Females [S], Age [C], Females [C], Age, Females, Diabetes, IVDU, Nephropathy, Epidural abscess, Date. - Length of stay meta-analysis: Author, n.s, mean.s, sd.s, n.c, mean.c, sd.c, Sample size, Study type, Study design, Age [S], Females [S], Age [C], Females [C], Age, Females, Diabetes, IVDU, Nephropathy, Epidural abscess, Date. |

In Supplementary Table 3, a table shows the extracted variables in the qualitative synthesis (systematic review) and quantitative synthesis (meta-analysis).

Supplementary Table 4: Risk of bias analysis of all included studies (ROBINS-I tool).

| **Study** | **D1** | **D2** | **D3** | **D4** | **D5** | **D6** | **D7** | **D8** |
| --- | --- | --- | --- | --- | --- | --- | --- | --- |
| Adogwa et al. (2014) | SERIOUS | MODERATE | LOW | LOW | LOW | LOW | LOW | MODERATE |
| Alas et al. (2020) | MODERATE | MODERATE | LOW | LOW | LOW | LOW | LOW | LOW |
| Berwick et al. (2019) | MODERATE | MODERATE | LOW | LOW | LOW | LOW | LOW | LOW |
| Canouï et al (2019) | MODERATE | LOW | LOW | LOW | LOW | LOW | LOW | LOW |
| Farber et al. (2016) | MODERATE | MODERATE | LOW | LOW | LOW | LOW | LOW | LOW |
| Giampaolini et al. (2022) | CRITICAL | MODERATE | LOW | LOW | LOW | LOW | LOW | SERIOUS |
| Hasan et al. (2013) | LOW | MODERATE | LOW | LOW | LOW | LOW | LOW | LOW |
| Hohenberger et al. (2022) | CRITICAL | MODERATE | LOW | LOW | LOW | LOW | LOW | SERIOUS |
| Jin et al. (2022) | MODERATE | MODERATE | LOW | LOW | LOW | LOW | LOW | LOW |
| Jung et al. (2021) | SERIOUS | LOW | LOW | LOW | LOW | LOW | LOW | MODERATE |
| Karikari et al. (2009) | SERIOUS | MODERATE | LOW | LOW | LOW | LOW | LOW | MODERATE |
| Kenzo et al. (2010) | CRITICAL | MODERATE | LOW | LOW | LOW | LOW | LOW | SERIOUS |
| Khanna et al. (1996) | SERIOUS | MODERATE | LOW | LOW | LOW | SERIOUS | LOW | SERIOUS |
| Kim et al. (2014) | LOW | LOW | LOW | LOW | LOW | LOW | LOW | LOW |
| Kreutzträger et al. (2022) | SERIOUS | MODERATE | LOW | LOW | LOW | LOW | LOW | MODERATE |
| Lee et al. (2021) | MODERATE | SERIOUS | LOW | LOW | MODERATE | LOW | LOW | MODERATE |
| Lener et al. (2020) | LOW | MODERATE | LOW | LOW | LOW | LOW | LOW | LOW |
| Mann et al. (2004) | CRITICAL | LOW | LOW | LOW | LOW | LOW | LOW | MODERATE |
| McHenry et al. (2002) | SERIOUS | LOW | LOW | LOW | LOW | LOW | LOW | MODERATE |
| Pitaro et al. (2021) | MODERATE | MODERATE | LOW | LOW | LOW | LOW | LOW | LOW |
| Segreto et al. (2018) | MODERATE | MODERATE | LOW | LOW | LOW | LOW | LOW | LOW |
| Shweikeh et al. (2017) | MODERATE | MODERATE | LOW | LOW | LOW | LOW | LOW | LOW |
| Sobbotke et al. (2009) | CRITICAL | MODERATE | LOW | LOW | LOW | LOW | LOW | SERIOUS |
| Sur et al. (2015) | SERIOUS | MODERATE | LOW | LOW | LOW | LOW | LOW | MODERATE |
| Tang et al. (2002) | LOW | MODERATE | LOW | LOW | LOW | LOW | LOW | LOW |
| Tani et al. (2022) | CRITICAL | MODERATE | LOW | LOW | LOW | LOW | LOW | SERIOUS |
| Tsai et al. (2017) | LOW | LOW | LOW | LOW | CRITICAL | LOW | LOW | CRITICAL |
| Valancius et al. (2013) | SERIOUS | MODERATE | LOW | LOW | LOW | LOW | LOW | MODERATE |
| Verla et al. (2020) | LOW | MODERATE | LOW | LOW | SERIOUS | LOW | LOW | MODERATE |
| William et al. (2005) | SERIOUS | MODERATE | LOW | LOW | LOW | LOW | LOW | MODERATE |
| Zadran et al. (2020) | LOW | MODERATE | LOW | LOW | LOW | LOW | LOW | LOW |

Supplementary Table 4 shows the results of the risk of bias analysis of all included studies using the ROBINS-I tool: Giampaloni et al. (2022), Hohenberger et al. (2022), Kenzo et al. (2022), Khanna et al. (1996), Sobbotke et al. (2009) and Tani et al. (2022) was scored as serious risk of bias as there was no sufficient analysis of confounding factors. Confounding factors include patient characteristics, and if omitted also leads to a lack of adjunct propensity score matching and sensitivity analyses, which are essential for validity of findings. Tsai et al. (2017) was scored as at critical risk of bias, as seven patients who initially met the criteria for Group 1 (conservative management group) later required surgery due to poor response to antibiotics and were subsequently no longer included in either group, and practically excluded from the subsequent analysis.

Supplementary Table 5: : Level of evidence of each of the included studies based on the Oxford Centre of Evidence-Based Medicine (OCEBM) Levels of Evidence.

| **Study number** | **Author, Year** | **Level of evidence** |
| --- | --- | --- |
| 1 | Adogwa et al. (2014) | 2b |
| 2 | Alas et al. (2020) | 2b |
| 3 | Berwick et al. (2019) | 2b |
| 4 | Canouï et al (2019) | 2b |
| 5 | Farber et al. (2016) | 2b |
| 6 | Giampaolini et al. (2022) | 2b |
| 7 | Hasan et al. (2013) | 4 |
| 8 | Hohenberger et al. (2022) | 2b |
| 9 | Jin et al. (2022) | 2b |
| 10 | Jung et al. (2021) | 2b |
| 11 | Karikari et al. (2009) | 2b |
| 12 | Kenzo et al. (2010) | 2b |
| 13 | Khanna et al. (1996) | 4 |
| 14 | Kim et al. (2014) | 3b |
| 15 | Kreutzträger et al. (2022) | 2b |
| 16 | Lee et al. (2021) | 2b |
| 17 | Lener et al. (2020) | 3b |
| 18 | Mann et al. (2004) | 4 |
| 19 | McHenry et al. (2002) | 2b |
| 20 | Pitaro et al. (2021) | 2b |
| 21 | Segreto et al. (2018) | 2b |
| 22 | Shweikeh et al. (2017) | 2b |
| 23 | Sobbotke et al. (2009) | 4 |
| 24 | Sur et al. (2015) | 4 |
| 25 | Tang et al. (2002) | 3b |
| 26 | Tani et al. (2022) | 2b |
| 27 | Tsai et al. (2017) | 2b |
| 28 | Valancius et al. (2013) | 2b |
| 29 | Verla et al. (2020) | 4 |
| 30 | William et al. (2005) | 4 |
| 31 | Zadran et al. (2020) | 2b |

Supplementary Table 5 shows the results of the risk of bias analysis of all included studies using the Oxford Centre of Evidence-Based Medicine (OCEBM) Levels of Evidence tool.

Supplementary Table 6: GRADE (Grading of Recommendations, Assessment, Development and Evaluations) scoring for all studies.

| Study | GRADE Risk of Bias | GRADE Imprecision | GRADE Incosistency | GRADE Indirectness | Publication bias | GRADE Overall |
| --- | --- | --- | --- | --- | --- | --- |
| Adogwa et al. (2014) | MODERATE | LOW | LOW | MODERATE | LOW | MODERATE |
| Alas et al. (2020) | LOW | LOW | LOW | LOW | LOW | HIGH |
| Berwick et al. (2019) | LOW | LOW | LOW | MODERATE | LOW | MODERATE |
| Canouï et al (2019) | LOW | LOW | LOW | LOW | LOW | HIGH |
| Farber et al. (2016) | LOW | LOW | LOW | MODERATE | LOW | MODERATE |
| Giampaolini et al. (2022) | HIGH | LOW | LOW | LOW | LOW | LOW |
| Hasan et al. (2013) | LOW | LOW | LOW | MODERATE | LOW | MODERATE |
| Hohenberger et al. (2022) | HIGH | LOW | MODERATE | LOW | LOW | LOW |
| Jin et al. (2022) | LOW | LOW | LOW | MODERATE | LOW | MODERATE |
| Jung et al. (2021) | MODERATE | LOW | LOW | MODERATE | LOW | MODERATE |
| Karikari et al. (2009) | MODERATE | LOW | LOW | MODERATE | LOW | MODERATE |
| Kenzo et al. (2010) | HIGH | LOW | LOW | MODERATE | LOW | LOW |
| Khanna et al. (1996) | HIGH | LOW | LOW | MODERATE | LOW | LOW |
| Kim et al. (2014) | LOW | LOW | LOW | MODERATE | LOW | MODERATE |
| Kreutzträger et al. (2022) | MODERATE | LOW | LOW | MODERATE | LOW | MODERATE |
| Lee et al. (2021) | MODERATE | MODERATE | LOW | LOW | LOW | MODERATE |
| Lener et al. (2020) | LOW | LOW | LOW | LOW | LOW | HIGH |
| Mann et al. (2004) | MODERATE | LOW | LOW | MODERATE | LOW | MODERATE |
| McHenry et al. (2002) | MODERATE | LOW | LOW | LOW | LOW | MODERATE |
| Pitaro et al. (2021) | LOW | LOW | LOW | LOW | LOW | HIGH |
| Segreto et al. (2018) | LOW | MODERATE | LOW | MODERATE | LOW | MODERATE |
| Shweikeh et al. (2017) | LOW | LOW | LOW | LOW | LOW | HIGH |
| Sobbotke et al. (2009) | HIGH | LOW | LOW | LOW | LOW | LOW |
| Sur et al. (2015) | MODERATE | MODERATE | LOW | MODERATE | LOW | LOW |
| Tang et al. (2002) | LOW | LOW | LOW | MODERATE | LOW | MODERATE |
| Tani et al. (2022) | HIGH | LOW | LOW | LOW | LOW | LOW |
| Tsai et al. (2017) | HIGH | LOW | MODERATE | LOW | LOW | VERY LOW |
| Valancius et al. (2013) | MODERATE | LOW | MODERATE | LOW | LOW | MODERATE |
| Verla et al. (2020) | MODERATE | LOW | LOW | MODERATE | LOW | MODERATE |
| William et al. (2005) | MODERATE | LOW | LOW | MODERATE | LOW | MODERATE |
| Zadran et al. (2020) | LOW | LOW | LOW | LOW | LOW | HIGH |

Supplementary Table 6 shows the results of the risk of bias analysis of all included studies using the GRADE (Grading of Recommendations, Assessment, Development and Evaluations) scoring.

Supplementary Table 7: The definitions of early and delayed surgery used by each study.

| **Study number** | **Author, Year** | **Definition of early and delayed surgery** |
| --- | --- | --- |
| 1 | Adogwa et al. (2014) | Not defined |
| 2 | Alas et al. (2020) | Not defined |
| 3 | Berwick et al. (2019) | Not defined, but surgical patients had surgery within 14 days of inpatient antibiotic treatment or else excluded |
| 4 | Canouï et al (2019) | Not defined |
| 5 | Farber et al. (2016) | Early: <48 hours from admission  Delayed: >48 hours from admission |
| 6 | Giampaolini et al. (2022) | Not defined |
| 7 | Hasan et al. (2013) | Not defined |
| 8 | Hohenberger et al. (2022) | Early: <10 days from admission  Delayed: >10 days from admission |
| 9 | Jin et al. (2022) | Not defined |
| 10 | Jung et al. (2021) | Not defined |
| 11 | Karikari et al. (2009) | Not defined |
| 12 | Kenzo et al. (2010) | Not defined |
| 13 | Khanna et al. (1996) | Not defined |
| 14 | Kim et al. (2014) | Early: ‘immediate’ surgical treatment after diagnosis  Delayed: After 1 week of antibiotic therapy |
| 15 | Kreutzträger et al. (2022) | Not defined |
| 16 | Lee et al. (2021) | Early: < 6 weeks from admission  Delayed: > 6 weeks (excluded from paper) |
| 17 | Lener et al. (2020) | Not defined |
| 18 | Mann et al. (2004) | Early: Immediately after diagnosis of spinal infection  Delayed: 3 or more days after diagnosis of spinal infection |
| 19 | McHenry et al. (2002) | Not defined |
| 20 | Pitaro et al. (2021) | Not defined |
| 21 | Segreto et al. (2018) | Stratified into:   - 0-day delay (same day) - 1 day delay - 2-day delay - 3–6-day delay - 7–14-day delay - 14–30-day delay |
| 22 | Shweikeh et al. (2017) | Not defined |
| 23 | Sobbotke et al. (2009) | Not defined |
| 24 | Sur et al. (2015) | Not defined |
| 25 | Tang et al. (2002) | Not defined |
| 26 | Tani et al. (2022) | Surgical group had ‘immediate’ surgery |
| 27 | Tsai et al. (2017) | Early: Immediately after confirmation of diagnosis |
| 28 | Valancius et al. (2013) | Immediate: directly after diagnosis  Early: Within 48 hours of diagnosis |
| 29 | Verla et al. (2020) | Not defined |
| 30 | William et al. (2005) | Early: Within 12 hours of diagnosis  Delayed: After 1 week of antibiotics |
| 31 | Zadran et al. (2020) | Not defined |

Supplementary Table 7 shows a comprehensive list of all the definitions used by studies to define early and delayed surgery.

Supplementary Table 8: The R code that was utilised for the meta-analyses and associated figures, ROB graphs,

as well as the meta-regressions and influence analyses.

#Install relevant packages

install.packages(c("robumeta", "metafor", "dplyr"))

install.packages("meta")

install.packages("dmetar")

install.packages("readxl")

#into library load

library("robumeta")

library("metafor")

library("dplyr")

library("mada")

library("meta")

library("dmetar")

library("readxl")

#install updated packaged for Rtools required now (2022)

write('PATH="${RTOOLS40_HOME}\\usr\\bin;${PATH}"', file = "~/.Renviron", append = TRUE)

Sys.which("make")

## "C:\\rtools40\\usr\\bin\\make.exe"

install.packages("jsonlite", type = "source")

#Install relevant packages

install.packages(c("robumeta", "metafor", "dplyr"))

install.packages("meta")

install.packages("devtools")

install.packages("readxl")

install.packages("devtools")

install_github("mcguinlu/robvis")

install.packages("robvis")

install.packages("utf8")

#into library load

library("robumeta")

library("metafor")

library("dplyr")

library("mada")

library("meta")

library("readxl")

library("robvis")

library("utf8")

if (!require("devtools")) {

install.packages("devtools")

}

devtools::install_github("MathiasHarrer/dmetar")

library(dmetar)

#ROB analysis

#loading excel sheet

library(readxl)

Spondylodiscitis_ROB <- read_excel("Desktop/Cambridge/Spondylodiscitis /Spondylodiscitis ROB Copy.xlsx")

#running Risk of bias analysis

rob_summary(Spondylodiscitis_ROB, tool = "ROBINS-I", weighted = FALSE , overall = TRUE)

####################### Study characteristics graphs ##################

library(readxl)

Spondylodiscitis_Study_Characteristics <- read_excel("Desktop/Cambridge/Spondylodiscitis /Spondylodiscitis Study Characteristics.xlsx")

####### 1. Make bar plot for study design

#count prospective, retrospective, ambispective

studydesigntable <- table(Spondylodiscitis_Study_Characteristics$`Study design`)

print(studydesigntable)

#Create dataframe

data1 <- data.frame(

name=c("Prospective", "Retrospective", "Ambispective") ,

value=c(3, 27, 1)

)

barplot_studytype_spond <- barplot(height = data1$value,

names.arg = data1$name,

xlab = "Study design",

ylab = "Number of studies",

ylim = c(0,30), xpd = TRUE,

col="black", angle = c(45, 90, 70), density = c(90,30,300))

####### 2. Make bar plot for study type

#count case-control, cohort and case series

studytypetable <- table(Spondylodiscitis_Study_Characteristics$`Study type`)

print(studytypetable)

#Create dataframe

data1 <- data.frame(

name=c("Cohort", "Case series", "Case-control") ,

value=c(21, 9, 1)

)

barplot_studytype_spond <- barplot(height = data1$value,

names.arg = data1$name,

xlab = "Study design",

ylab = "Number of studies",

ylim = c(0,25), xpd = TRUE,

col=c("black", "grey", "white"))

###### 3. Make bar plot for study sample size

library(readxl)

Spondylodiscitis_Study_Characteristics <- read_excel("Desktop/Cambridge/Spondylodiscitis /Spondylodiscitis Study Characteristics NEW.xlsx")

data2 <- data.frame(

name=Spondylodiscitis_Study_Characteristics$Study,

value=Spondylodiscitis_Study_Characteristics$`Sample size`

)

library(RColorBrewer)

n <- 60

qual_col_pals = brewer.pal.info[brewer.pal.info$category == 'qual',]

col_vector = unlist(mapply(brewer.pal, qual_col_pals$maxcolors, rownames(qual_col_pals)))

pie(rep(1,n), col=sample(col_vector, n))

p <- barplot(height = data2$value,

names.arg = data2$name,

xlab = "Study",

ylab = "Sample size",

ylim = c(0, 37000),

col = col_vector,

xpd = TRUE,

beside = TRUE,

legend.text = Spondylodiscitis_Study_Characteristics$Author,

args.legend = list(xjust = 0.15,

cex = 0.6, x.intersp = 0.1, bty = "n"))

#make broken bar plot with ggplot2

library(tidyverse)

install.packages("randomcoloR")

library(randomcoloR)

install.packages("ggbreak")

library(ggbreak)

distinctColorPalette(k = 31, altCol = FALSE, runTsne = FALSE)

Study <- Spondylodiscitis_Study_Characteristics$Study

Sample.Size <- Spondylodiscitis_Study_Characteristics$`Sample size`

Author <- Spondylodiscitis_Study_Characteristics$Author

p1 <- ggplot(data=data2,

aes(x=Study, y=Sample.Size, fill=Author)) +

geom_bar(stat="identity" , width=0.5) + theme_minimal() +

scale_fill_manual(values=c("#D0E7E9","#E45456", "#C268DD", "#B8A2DE", "#8EBFDD",

"#50635F", "#ECE440", "#67E949", "#E4C8E2", "#646BCB",

"#B6A69E", "#D49653", "#E1C34E", "#67EAC9", "#BDE856",

"#D64BA2", "#DC3FEA", "#E096E5", "#BBEEC7", "#74A1DF",

"#7046DE", "#7DB8A7", "#72AF5A", "#D8C08D", "#D17CA1",

"#79E2E7", "#60E37A", "#E4DCC2", "#EAA09C", "#DAE999", "#97E997"))

p1

p2 <- p1 + scale_y_break(c(710, 10100, 10200, 34400))

p2

randomColor(count=31)

######make plot for world map

install.packages(c("cowplot", "googleway", "ggplot2", "ggrepel",

"ggspatial", "libwgeom", "sf", "rnaturalearth", "rnaturalearthdata"))

install.packages("rworldmap",dependencies=TRUE)

library("ggplot2")

theme_set(theme_bw())

library("sf")

#prep

Spondylodiscitis_World_Map <- read_excel("Desktop/Cambridge/Spondylodiscitis /Spondylodiscitis World Map.xlsx")

worldprep <- joinCountryData2Map(Spondylodiscitis_World_Map, joinCode = "ISO3", nameJoinColumn = "ISO3V10", nameCountryColumn = "Country", suggestForFailedCodes = FALSE, mapResolution = "coarse", projection = NA, verbose = FALSE)

worldmapspondy <- mapCountryData(mapToPlot = worldprep, nameColumnToPlot = "Studies",

xlim = NA, ylim = NA, mapRegion = "world",

catMethod = c(0:14), colourPalette = "rainbow",

addLegend = FALSE, borderCol = "black", mapTitle = "Number of studies per country",

aspect = 1, missingCountryCol = NA, add = FALSE,

nameColumnToHatch = TRUE, lwd = 0.5, oceanCol = NA)

do.call(addMapLegendBoxes

, c(worldmapspondy

,list(legendText=c("1","2","5","13")

, x="top",title="Number of studies",horiz=TRUE)))

###### 4. Make bar plot for study year

studysamplesizetable <- table(Spondylodiscitis_Study_Characteristics$Year)

print(studysamplesizetable)

plot(studysamplesizetable)

data3 <- data.frame(

year=c(1996:2022),

number=c(1,0,0,0,0,0,2,0,1,1,0,0,0,2,1,0,0,1,2,1,0,3,1,1,4,3,7)

)

barplot(height = data3$value,

names.arg = data3$name,

xlab = "Year of study publication",

ylab = "Number of studies",

col = "black",

ylim = NULL, xpd = TRUE)

gap.barplot(data3,gap,xaxlab,xtics,yaxlab,ytics,xlim=NA,ylim=NA,xlab=NULL,

ylab=NULL,horiz=FALSE,col,...)

library(ggplot2)

library(dplyr)

install.packages("ggthemes")

library(ggthemes)

# Plot

data3 %>%

ggplot(aes(x=year, y=number)) +

geom_line(linetype="dashed", color="black", size=0.3) +

geom_point(size=3, colour = "black") + theme_minimal() +

theme(axis.text.x = element_text(angle = 90, vjust = 0.5)) +

labs(y = "number of studies", x ="year of publication") +

scale_x_continuous(name= waiver(), breaks = data3$year) +

scale_y_continuous(name= waiver(), breaks = 1:7)

######################## Meta-analysis: Relapse Failure ########################################

#load Excel file

library(readxl)

MA_Spondylodiscitis_Relapse_or_Failure <- read_excel("Desktop/Cambridge/Spondylodiscitis /Meta-analysis Spondylodiscitis Relapse or Failure.xlsx")

#meta-analysis

meta_Spondylodiscitis_Relapse <- metagen(TE = MA_Spondylodiscitis_Relapse_or_Failure$TE,

seTE = MA_Spondylodiscitis_Relapse_or_Failure$seTE,

studlab = MA_Spondylodiscitis_Relapse_or_Failure$Author,

title = "Relative Risk: Relapse",

data = MA_Spondylodiscitis_Relapse_or_Failure,

sm = "",

fixed = FALSE,

random = TRUE,

method.tau = "DL",

lower = MA_Spondylodiscitis_Relapse_or_Failure$`lower `,

upper = MA_Spondylodiscitis_Relapse_or_Failure$upper,

pval = MA_Spondylodiscitis_Relapse_or_Failure$pval,

hakn = TRUE,

n.e = MA_Spondylodiscitis_Relapse_or_Failure$`Sample `,

text.random = "Relative Risk",

label.e = "",

prediction = TRUE,

backtransf = TRUE)

forest.meta(meta_Spondylodiscitis_Relapse,

lab.e = "",

JAMA.pval = FALSE,

test.overall.random = TRUE,

fs.hetstat = 9)

#find outliers

find.outliers(meta_Spondylodiscitis_Relapse) #Hohenberg et al. is outlier, pitaro maybe too?

#make graph without Hohenberg - STILL NEEDS TO BE DONE

Spondy_Rel <- InfluenceAnalysis(meta_Spondylodiscitis_Relapse)

plot(Spondy_Rel)

#Meta-analysis without Hohenberger

library(readxl)

Meta_analysis_Spondylodiscitis_Relapse_or_Failure_without_Hohenberger <- read_excel("Desktop/Cambridge/Spondylodiscitis /Meta-analysis Spondylodiscitis Relapse or Failure without Hohenberger.xlsx")

#meta-analysis

meta_Spondylodiscitis_Relapse_without_Hohenberger <- metagen(TE = Meta_analysis_Spondylodiscitis_Relapse_or_Failure_without_Hohenberger$TE,

seTE = Meta_analysis_Spondylodiscitis_Relapse_or_Failure_without_Hohenberger$seTE,

studlab = Meta_analysis_Spondylodiscitis_Relapse_or_Failure_without_Hohenberger$Author,

title = "Relative Risk: Relapse",

data = Meta_analysis_Spondylodiscitis_Relapse_or_Failure_without_Hohenberger,

sm = "",

fixed = FALSE,

random = TRUE,

method.tau = "DL",

lower = Meta_analysis_Spondylodiscitis_Relapse_or_Failure_without_Hohenberger$`lower `,

upper = Meta_analysis_Spondylodiscitis_Relapse_or_Failure_without_Hohenberger$upper,

pval = Meta_analysis_Spondylodiscitis_Relapse_or_Failure_without_Hohenberger$pval,

hakn = TRUE,

n.e = Meta_analysis_Spondylodiscitis_Relapse_or_Failure_without_Hohenberger$`Sample `,

text.random = "Relative Risk",

label.e = "",

prediction = TRUE,

backtransf = TRUE)

forest.meta(meta_Spondylodiscitis_Relapse_without_Hohenberger,

lab.e = "",

JAMA.pval = FALSE,

test.overall.random = TRUE,

fs.hetstat = 9)

#Meta-analysis without Pitaro

library(readxl)

Meta_analysis_Spondylodiscitis_Relapse_or_Failure_without_Pitaro <- read_excel("Desktop/Cambridge/Spondylodiscitis /Meta-analysis Spondylodiscitis Relapse or Failure without Pitaro.xlsx")

#meta-analysis

meta_Spondylodiscitis_Relapse_without_Pitataro <- metagen(TE = Meta_analysis_Spondylodiscitis_Relapse_or_Failure_without_Pitaro$TE,

seTE = Meta_analysis_Spondylodiscitis_Relapse_or_Failure_without_Pitaro$seTE,

studlab = Meta_analysis_Spondylodiscitis_Relapse_or_Failure_without_Pitaro$Author,

title = "Relative Risk: Relapse",

data = Meta_analysis_Spondylodiscitis_Relapse_or_Failure_without_Pitaro,

sm = "",

fixed = FALSE,

random = TRUE,

method.tau = "DL",

lower = Meta_analysis_Spondylodiscitis_Relapse_or_Failure_without_Pitaro$`lower `,

upper = Meta_analysis_Spondylodiscitis_Relapse_or_Failure_without_Pitaro$upper,

pval = Meta_analysis_Spondylodiscitis_Relapse_or_Failure_without_Pitaro$pval,

hakn = TRUE,

n.e = Meta_analysis_Spondylodiscitis_Relapse_or_Failure_without_Pitaro$`Sample `,

text.random = "Relative Risk",

label.e = "",

prediction = TRUE,

backtransf = TRUE)

forest.meta(meta_Spondylodiscitis_Relapse_without_Pitataro,

lab.e = "",

JAMA.pval = FALSE,

test.overall.random = TRUE,

fs.hetstat = 9)

#Meta-analysis without Hohenberger and Pitaro

library(readxl)

Meta_analysis_Spondylodiscitis_Relapse_or_Failure_without_Hohenberg_and_Pitaro <- read_excel("Desktop/Cambridge/Spondylodiscitis /Meta-analysis Spondylodiscitis Relapse or Failure without Hohenberg and Pitaro.xlsx")

#meta-analysis

meta_Spondylodiscitis_Relapse_without_Hohenberg_and_Pitaro <- metagen(TE = Meta_analysis_Spondylodiscitis_Relapse_or_Failure_without_Hohenberg_and_Pitaro$TE,

seTE = Meta_analysis_Spondylodiscitis_Relapse_or_Failure_without_Hohenberg_and_Pitaro$seTE,

studlab = Meta_analysis_Spondylodiscitis_Relapse_or_Failure_without_Hohenberg_and_Pitaro$Author,

title = "Relative Risk: Relapse",

data = Meta_analysis_Spondylodiscitis_Relapse_or_Failure_without_Hohenberg_and_Pitaro,

sm = "",

fixed = FALSE,

random = TRUE,

method.tau = "DL",

lower = Meta_analysis_Spondylodiscitis_Relapse_or_Failure_without_Hohenberg_and_Pitaro$`lower `,

upper = Meta_analysis_Spondylodiscitis_Relapse_or_Failure_without_Hohenberg_and_Pitaro$upper,

pval = Meta_analysis_Spondylodiscitis_Relapse_or_Failure_without_Hohenberg_and_Pitaro$pval,

hakn = TRUE,

n.e = Meta_analysis_Spondylodiscitis_Relapse_or_Failure_without_Hohenberg_and_Pitaro$`Sample `,

text.random = "Relative Risk",

label.e = "",

prediction = TRUE,

backtransf = TRUE)

forest.meta(meta_Spondylodiscitis_Relapse_without_Hohenberg_and_Pitaro,

lab.e = "",

JAMA.pval = FALSE,

test.overall.random = TRUE,

fs.hetstat = 9)

#post-meta-regression analyses

#Meta-analysis without Hohenberger and Pitaro

library(readxl)

Meta_analysis_Spondylodiscitis_Relapse_or_Failure_without_Hohenberger_and_Hasan <- read_excel("Desktop/Cambridge/Spondylodiscitis /Meta-analysis Spondylodiscitis Relapse or Failure without Hohenberger and Hasan.xlsx")

#meta-analysis

meta_Spondylodiscitis_Relapse_without_Hohenberg_and_Hasan <- metagen(TE = Meta_analysis_Spondylodiscitis_Relapse_or_Failure_without_Hohenberger_and_Hasan$TE,

seTE = Meta_analysis_Spondylodiscitis_Relapse_or_Failure_without_Hohenberger_and_Hasan$seTE,

studlab = Meta_analysis_Spondylodiscitis_Relapse_or_Failure_without_Hohenberger_and_Hasan$Author,

title = "Relative Risk: Relapse",

data = Meta_analysis_Spondylodiscitis_Relapse_or_Failure_without_Hohenberger_and_Hasan,

sm = "",

fixed = FALSE,

random = TRUE,

method.tau = "DL",

lower = Meta_analysis_Spondylodiscitis_Relapse_or_Failure_without_Hohenberger_and_Hasan$`lower `,

upper = Meta_analysis_Spondylodiscitis_Relapse_or_Failure_without_Hohenberger_and_Hasan$upper,

pval = Meta_analysis_Spondylodiscitis_Relapse_or_Failure_without_Hohenberger_and_Hasan$pval,

hakn = TRUE,

n.e = Meta_analysis_Spondylodiscitis_Relapse_or_Failure_without_Hohenberger_and_Hasan$`Sample `,

text.random = "Relative Risk",

label.e = "",

prediction = TRUE,

backtransf = TRUE)

forest.meta(meta_Spondylodiscitis_Relapse_without_Hohenberg_and_Hasan,

lab.e = "",

JAMA.pval = FALSE,

test.overall.random = TRUE,

fs.hetstat = 9)

### Meta-regression: Relapse/Failure ################

#variables only included if half of included studies reported on it

#sample size - insignificant

meta_Spondy_Sample <- metareg(meta_Spondylodiscitis_Relapse,

~ `Sample size`)

print(meta_Spondy_Sample)

#study type - insignificant

meta_Spondy_StudyType <- metareg(meta_Spondylodiscitis_Relapse,

~ `Study type`)

print(meta_Spondy_StudyType)

#Study design - insignificant

meta_Spondy_StudyDesign <- metareg(meta_Spondylodiscitis_Relapse,

~ `Study design`)

print(meta_Spondy_StudyDesign)

#Age [S] - insignificant

meta_Spondy_AgeS <- metareg(meta_Spondylodiscitis_Relapse,

~ `Age [S]`)

print(meta_Spondy_AgeS)

#Age [C] - insignificant

meta_Spondy_AgeC <- metareg(meta_Spondylodiscitis_Relapse,

~ `Age [C]`)

print(meta_Spondy_AgeC)

#Females [S] - insignificant

meta_Spondy_FemaleS <- metareg(meta_Spondylodiscitis_Relapse,

~ `Females [S]`)

print(meta_Spondy_FemaleS)

#Females [C] - insignificant

meta_Spondy_FemaleC <- metareg(meta_Spondylodiscitis_Relapse,

~ `Females [C]`)

print(meta_Spondy_FemaleC)

#Age - insignificant

meta_Spondy_Age <- metareg(meta_Spondylodiscitis_Relapse,

~ `Age`)

print(meta_Spondy_Age)

#Females - insignificant

meta_Spondy_Female <- metareg(meta_Spondylodiscitis_Relapse,

~ `Females`)

print(meta_Spondy_Female)

#Diabetes - insignificant

meta_Spondy_Diabetes <- metareg(meta_Spondylodiscitis_Relapse,

~ `Diabetes`)

print(meta_Spondy_Diabetes)

#IVDU - significant - -0.0175 - means better outcomes with surgery

meta_Spondy_IVDU <- metareg(meta_Spondylodiscitis_Relapse,

~ `IVDU`)

print(meta_Spondy_IVDU)

#Nephropathy - insignificant

meta_Spondy_Nephro <- metareg(meta_Spondylodiscitis_Relapse,

~ `Nephropathy`)

print(meta_Spondy_Nephro)

#epidural abscess - insignificant

meta_Spondy_EA <- metareg(meta_Spondylodiscitis_Relapse,

~ `Epidural abscess`)

print(meta_Spondy_EA)

#Date of publication - insignificant

meta_Spondy_Date <- metareg(meta_Spondylodiscitis_Relapse,

~ `Date`)

print(meta_Spondy_Date)

######################## Meta-analysis: Mortality ########################################

library(readxl)

Meta_analysis_Spondylodiscitis_Mortality <- read_excel("Desktop/Cambridge/Spondylodiscitis /Meta-analysis Spondylodiscitis Mortality.xlsx")

#meta-analysis

meta_Spondylodiscitis_Mortality <- metagen(TE = Meta_analysis_Spondylodiscitis_Mortality$TE,

seTE = Meta_analysis_Spondylodiscitis_Mortality$seTE,

studlab = Meta_analysis_Spondylodiscitis_Mortality$Author,

title = "Relative Risk: Relapse",

data = Meta_analysis_Spondylodiscitis_Mortality,

sm = "",

fixed = FALSE,

random = TRUE,

method.tau = "DL",

lower = Meta_analysis_Spondylodiscitis_Mortality$`lower `,

upper = Meta_analysis_Spondylodiscitis_Mortality$upper,

pval = Meta_analysis_Spondylodiscitis_Mortality$pval,

hakn = TRUE,

n.e = Meta_analysis_Spondylodiscitis_Mortality$`Sample `,

text.random = "Relative Risk",

label.e = "",

prediction = TRUE,

backtransf = TRUE)

forest.meta(meta_Spondylodiscitis_Mortality,

lab.e = "",

JAMA.pval = FALSE,

test.overall.random = TRUE,

fs.hetstat = 9)

#find outliers -> no outliers detected for mortality

find.outliers(meta_Spondylodiscitis_Mortality) #Hohenberg et al. is outlier, pitaro maybe too?

Spondy_Mort <- InfluenceAnalysis(meta_Spondylodiscitis_Mortality)

plot(Spondy_Mort)

#Meta-analysis Mortality without Alas

library(readxl)

Meta_analysis_Spondylodiscitis_Mortality_without_Alas <- read_excel("Desktop/Cambridge/Spondylodiscitis /Meta-analysis Spondylodiscitis Mortality without Alas.xlsx")

#meta-analysis

meta_Spondylodiscitis_Mortality_without_Alas <- metagen(TE = Meta_analysis_Spondylodiscitis_Mortality_without_Alas$TE,

seTE = Meta_analysis_Spondylodiscitis_Mortality_without_Alas$seTE,

studlab = Meta_analysis_Spondylodiscitis_Mortality_without_Alas$Author,

title = "Relative Risk: Relapse",

data = Meta_analysis_Spondylodiscitis_Mortality_without_Alas,

sm = "",

fixed = FALSE,

random = TRUE,

method.tau = "DL",

lower = Meta_analysis_Spondylodiscitis_Mortality_without_Alas$`lower `,

upper = Meta_analysis_Spondylodiscitis_Mortality_without_Alas$upper,

pval = Meta_analysis_Spondylodiscitis_Mortality_without_Alas$pval,

hakn = TRUE,

n.e = Meta_analysis_Spondylodiscitis_Mortality_without_Alas$`Sample `,

text.random = "Relative Risk",

label.e = "",

prediction = TRUE,

backtransf = TRUE)

forest.meta(meta_Spondylodiscitis_Mortality_without_Alas,

lab.e = "",

JAMA.pval = FALSE,

test.overall.random = TRUE,

fs.hetstat = 9)

#Meta-analysis Mortality without Alas

library(readxl)

Meta_analysis_Spondylodiscitis_Mortality_without_Lee <- read_excel("Desktop/Cambridge/Spondylodiscitis /Meta-analysis Spondylodiscitis Mortality without Lee.xlsx")

#meta-analysis

meta_Spondylodiscitis_Mortality_without_Lee <- metagen(TE = Meta_analysis_Spondylodiscitis_Mortality_without_Lee$TE,

seTE = Meta_analysis_Spondylodiscitis_Mortality_without_Lee$seTE,

studlab = Meta_analysis_Spondylodiscitis_Mortality_without_Lee$Author,

title = "Relative Risk: Relapse",

data = Meta_analysis_Spondylodiscitis_Mortality_without_Lee,

sm = "",

fixed = FALSE,

random = TRUE,

method.tau = "DL",

lower = Meta_analysis_Spondylodiscitis_Mortality_without_Lee$`lower `,

upper = Meta_analysis_Spondylodiscitis_Mortality_without_Lee$upper,

pval = Meta_analysis_Spondylodiscitis_Mortality_without_Lee$pval,

hakn = TRUE,

n.e = Meta_analysis_Spondylodiscitis_Mortality_without_Lee$`Sample `,

text.random = "Relative Risk",

label.e = "",

prediction = TRUE,

backtransf = TRUE)

forest.meta(meta_Spondylodiscitis_Mortality_without_Lee,

lab.e = "",

JAMA.pval = FALSE,

test.overall.random = TRUE,

fs.hetstat = 9)

#Meta-analysis Mortality without Alas

library(readxl)

Meta_analysis_Spondylodiscitis_Mortality_without_Alas_and_Lee <- read_excel("Desktop/Cambridge/Spondylodiscitis /Meta-analysis Spondylodiscitis Mortality without Alas and Lee.xlsx")

#meta-analysis

meta_analysis_Spondylodiscitis_Mortality_without_Alas_and_Lee <- metagen(TE = Meta_analysis_Spondylodiscitis_Mortality_without_Alas_and_Lee$TE,

seTE = Meta_analysis_Spondylodiscitis_Mortality_without_Alas_and_Lee$seTE,

studlab = Meta_analysis_Spondylodiscitis_Mortality_without_Alas_and_Lee$Author,

title = "Relative Risk: Relapse",

data = Meta_analysis_Spondylodiscitis_Mortality_without_Alas_and_Lee,

sm = "",

fixed = FALSE,

random = TRUE,

method.tau = "DL",

lower = Meta_analysis_Spondylodiscitis_Mortality_without_Alas_and_Lee$`lower `,

upper = Meta_analysis_Spondylodiscitis_Mortality_without_Alas_and_Lee$upper,

pval = Meta_analysis_Spondylodiscitis_Mortality_without_Alas_and_Lee$pval,

hakn = TRUE,

n.e = Meta_analysis_Spondylodiscitis_Mortality_without_Alas_and_Lee$`Sample `,

text.random = "Relative Risk",

label.e = "",

prediction = TRUE,

backtransf = TRUE)

forest.meta(meta_analysis_Spondylodiscitis_Mortality_without_Alas_and_Lee,

lab.e = "",

JAMA.pval = FALSE,

test.overall.random = TRUE,

fs.hetstat = 9)

### Meta-regression: Mortality ################

#variables only included if half of included studies reported on it

#sample size - insignificant

meta_Spondy_SampleM <- metareg(meta_Spondylodiscitis_Mortality,

~ `Sample size`)

print(meta_Spondy_SampleM)

#study type - insignificant

meta_Spondy_StudyTypeM <- metareg(meta_Spondylodiscitis_Mortality,

~ `Study type`)

print(meta_Spondy_StudyTypeM)

#Study design - insignificant

meta_Spondy_StudyDesignM <- metareg(meta_Spondylodiscitis_Mortality,

~ `Study design`)

print(meta_Spondy_StudyDesignM)

#Age [S] - insignificant

meta_Spondy_AgeSM <- metareg(meta_Spondylodiscitis_Mortality,

~ `Age [S]`)

print(meta_Spondy_AgeSM)

#Age [C] - insignificant

meta_Spondy_AgeCM <- metareg(meta_Spondylodiscitis_Mortality,

~ `Age [C]`)

print(meta_Spondy_AgeCM)

#Females [S] - insignificant

meta_Spondy_FemaleSM <- metareg(meta_Spondylodiscitis_Mortality,

~ `Females [S]`)

print(meta_Spondy_FemaleSM)

#Females [C] - insignificant

meta_Spondy_FemaleCM <- metareg(meta_Spondylodiscitis_Mortality,

~ `Females [C]`)

print(meta_Spondy_FemaleCM)

#Age - insignificant

meta_Spondy_AgeM <- metareg(meta_Spondylodiscitis_Mortality,

~ `Age`)

print(meta_Spondy_AgeM)

#Females - insignificant

meta_Spondy_FemaleM <- metareg(meta_Spondylodiscitis_Mortality,

~ `Females`)

print(meta_Spondy_FemaleM)

#Diabetes - insignificant

meta_Spondy_DiabetesM <- metareg(meta_Spondylodiscitis_Mortality,

~ `Diabetes`)

print(meta_Spondy_DiabetesM)

#IVDU - significant - insignificant

meta_Spondy_IVDUM <- metareg(meta_Spondylodiscitis_Mortality,

~ `IVDU`)

print(meta_Spondy_IVDUM)

#Nephropathy - insignificant

meta_Spondy_NephroM <- metareg(meta_Spondylodiscitis_Mortality,

~ `Nephropathy`)

print(meta_Spondy_NephroM)

#epidural abscess - insignificant

meta_Spondy_EAM <- metareg(meta_Spondylodiscitis_Mortality,

~ `Epidural abscess`)

print(meta_Spondy_EAM)

#Date of publication - insignificant

meta_Spondy_DateM <- metareg(meta_Spondylodiscitis_Mortality,

~ `Date`)

print(meta_Spondy_DateM)

#Location of infection: Cervical- insignificant

meta_Spondy_Cervical <- metareg(meta_Spondylodiscitis_Mortality,

~ `Location of infection: Cervical`)

print(meta_Spondy_Cervical)

#Location of infection: Thoracic - insignificant

meta_Spondy_Thorax <- metareg(meta_Spondylodiscitis_Mortality,

~ `Location of infection: Thoracic`)

print(meta_Spondy_Thorax)

#Location of infection: Lumbar- insignificant

meta_Spondy_Lumbar <- metareg(meta_Spondylodiscitis_Mortality,

~ `Location of infection: Lumbar`)

print(meta_Spondy_Lumbar)

####################### Imaging characteristics graphs ##################

#Imaging modality

studyradiotable <- table(Heatmap$`Imaging modality`)

data4 <- data.frame(

name=c( "1.5T MRI", "1.5T or 0.5T MRI", "3T MRI",

"0.5-3T MRI", "Undefined MRI", "CTC",

"CT or MRI", "SPECT", "PEG"),

value=c(8, 1,5,2,1,2,4,2,3)

)

library(RColorBrewer)

coul <- brewer.pal(11, "PiYG")

barplot(height = data4$value,

names.arg = data4$name,

xlab = "Imaging modality",

ylab = "Number of studies",

col = c("palegreen", "seagreen1", "seagreen2", "seagreen3", "seagreen4",

"slateblue1", "plum2", "blue", "salmon"),

ylim = NULL, xpd = TRUE)

######### sensitivity analysis ############

# Load Total set dataset from dmetar (or download and open manually)

library(readxl)

Spondylo_Sens <- read_excel("Desktop/Cambridge/Spondylodiscitis /Spondylodiscitis Sensitivity analysis.xlsx")

# Use metcont to pool results.

meta_Total_sens_Spondy <- metagen(Spondylo_Sens$TE,

Spondylo_Sens$seTE,

data = Spondylo_Sens,

subset = NULL,

exclude = NULL,

id = NULL,

sm = "",

level = gs("level"),

level.ma = gs("level.ma"),

fixed = gs("fixed"),

hakn = gs("hakn"),

adhoc.hakn = gs("adhoc.hakn"),

method.tau = gs("method.tau"),

method.tau.ci = gs("method.tau.ci"),

tau.preset = NULL,

TE.tau = NULL,

tau.common = gs("tau.common"),

detail.tau = "",

prediction = gs("prediction"),

level.predict = gs("level.predict"),

null.effect = 0,

method.bias = gs("method.bias"),

n.e = NULL,

n.c = NULL,

Spondylo_Sens$pval,

level.ci = 0.95,

method.mean = "Luo",

method.sd = "Shi",

backtransf = gs("backtransf"),

pscale = 1,

irscale = 1,

irunit = "person-years",

text.fixed = gs("text.fixed"),

text.random = gs("text.random"),

text.predict = gs("text.predict"),

text.w.fixed = gs("text.w.fixed"),

text.w.random = gs("text.w.random"),

title = gs("title"),

complab = gs("complab"),

outclab = "",

label.e = gs("label.e"),

label.c = gs("label.c"),

label.left = gs("label.left"),

label.right = gs("label.right")

)

#numerical visualisation of meta_ttau (note that SR are the experimental group so reference group, so minus result means it is less in them)

print(meta_Total_sens_Spondy)

#make the plot

forest.meta(meta_Total_sens_Spondy,

sortvar = TE,

lab.e = "",

lab.c = "",

JAMA.pval = FALSE,

test.overall.random = TRUE,

label.test.overall.random = "Overall statistical result of model: ")

#Eggers (Publication bias) calculation

eggersplot <- metabias(

meta_Total_sens_Spondy,

method.bias = meta_Total_sens_Spondy$method.bias,

plotit = TRUE,

correct = FALSE,

k.min = 1

)

print(eggersplot)

print(eggersplot,

digits = gs("digits"),

digits.stat = gs("digits.stat"),

digits.pval = max(gs("digits.pval"), 2),

digits.se = gs("digits.se"),

digits.tau2 = gs("digits.tau2"),

scientific.pval = gs("scientific.pval"),

big.mark = gs("big.mark"),

zero.pval = gs("zero.pval"),

JAMA.pval = gs("JAMA.pval"),

text.tau2 = gs("text.tau2"))

#Eggers (Publication bias) plot

eggersplot2 <-metabias(

meta_Total_sens_Spondy,

method.bias = meta_Total_sens_Spondy$method.bias,

plotit = TRUE,

correct = FALSE,

k.min = 1

)

funnel.meta(meta_Total_sens_Spondy,

xlim = c(-100, 100),

studlab = FALSE,

method.bias = "linreg")

################ correlation plot ##############

library(readxl)

Correlation_Spondy <- read_excel("Desktop/Cambridge/Spondylodiscitis /Correlation analysis August.xlsx")

install.packages("corrplot")

library("corrplot")

CorRadio <- cor(Correlation_Spondy, use="pairwise.complete.obs")

#option 1

testRes = cor.mtest(CorRadio, conf.level = 0.95)

corrplot(CorRadio, p.mat = testRes$p, method = "circle", type = "lower", insig="blank",

addCoef.col ='black', number.cex = 0.5, order = "AOE", diag=FALSE)

#option 2

corrplot(CorRadio, p.mat = testRes$p, method = 'color', diag = FALSE, type = 'upper',

sig.level = c(0.001, 0.01, 0.05), pch.cex = 0.9, number.cex = 0.7, tl.col="black",

insig = 'label_sig', pch.col = 'grey20', order = 'AOE', col = COL2('RdYlBu'))

#option 3

library(PerformanceAnalytics)

install.packages("psych")

library(psych)

corpl(CorRadio$Age, CorRadio$EI)

pairs.panels(CorRadio,

smooth = TRUE, # If TRUE, draws loess smooths

scale = FALSE, # If TRUE, scales the correlation text font

density = TRUE, # If TRUE, adds density plots and histograms

ellipses = TRUE, # If TRUE, draws ellipses

method = "pearson", # Correlation method (also "spearman" or "kendall")

pch = 10, # pch symbol

lm = FALSE, # If TRUE, plots linear fit rather than the LOESS (smoothed) fit

cor = TRUE, # If TRUE, reports correlations

jiggle = FALSE, # If TRUE, data points are jittered

factor = 2, # Jittering factor

hist.col = 4, # Histograms color

stars = TRUE, # If TRUE, adds significance level with stars

ci = TRUE,

cex.labels = 0.4) # If TRUE, adds confidence intervals

install.packages("car")

library(car)

scatterplotMatrix(~ Sample + Age + Females + DM + HTN + Gaitdef + Cognitiondef + Urinedef + MMSE + EI + CA + SR + Complic, data = CorRadio,

diagonal = FALSE, # Remove kernel density estimates

regLine = list(col = "green", # Linear regression line color

lwd = 3), # Linear regression line width

smooth = list(col.smooth = "red", # Non-parametric mean color

col.spread = "blue",

legend.plot = TRUE)) # Non-parametric variance color

Supplementary Table 8 shows the complete R code that was utilised to compute the meta-analyses and associated figures, ROB graphs, as well as the meta-regressions and influence analyses.

Supplementary File 1: A detailed account of the statistical methodology employed in meta-analyses, meta-regressions and influence analyses.

The complete R code is shown in Supplementary Table 7. Firstly, a proportional meta-analysis was performed for mortality and relapse/failure, assessing the pooled proportion of mortality and relapse/failure among patients treated with early surgery and conversative treatment. A random-effects sub-group meta-analysis was conducted for each of relapse/failure, mortality and length of stay, comparing early surgical management (treatment group) to conservative management (control group). Studies must have included the following information: sample size for surgical management group and conservative management group; the number of positive and negative outcomes (relapse/failure or mortality) or length of stay in the early surgical management group and conservative management group, respectively. These values were needed to calculate the treatment effect sizes, namely the relative risk and mean difference. The inverse variance method was used for pooling effect sizes. The Hartung-Knapp method was used to adjust test statistics and confidence intervals. The Restricted maximum-likelihood estimator was used to analyse variance between studies. The *t*-test was used to calculate the overall statistical result of each meta-analysis with the associated p-value. Heterogeneity was estimated using the chi-squared statistic (*I*^2^) with the associated p-value. A statistical significance was assumed for p < 0.05. A sensitivity analysis was performed in two steps. Firstly, if included studies for each radiological marker included in the meta-analysis were rated at “serious” or “critical” overall risk of bias according to ROBINS-I tool, an additional sub-group random-effects meta-analysis without these studies was performed. Secondly, a multivariate mixed-effects meta-regression model was built and calculated. The following regression equation was employed:

#

$${\hat{\boldsymbol{\theta}}}_{\boldsymbol{k}}\boldsymbol{= \theta+}\boldsymbol{\beta}_{\boldsymbol{1}}\boldsymbol{x}_{\boldsymbol{k}}\boldsymbol{+}\boldsymbol{\epsilon}_{\boldsymbol{k}}\boldsymbol{+}\boldsymbol{\zeta}_{\boldsymbol{k}}$$

Reading the equation left to right, $\hat{\theta}_{k}$ denotes the observed effect size of each study ($k$) and acts as the dependent variable. $\theta$ denotes the y-axis intercept, and $\beta_{1}x_{k}$ is the independent variable, an arm-level covariate vector. The variables $\epsilon_{k}$and $\zeta_{k}$ denote two independent error variables. $\zeta_{k}$ explains that even the measured true effect size of each study is merely sampled from an overarching effect size distribution, which implies that heterogeneity variance exists between studies. The error term $\epsilon_{k}$describes the underlying independent sampling error which causes the effect size of a study to deviate from the true effect size. In this study, the following explanatory variables model was chosen to explain and represent the error term $\epsilon_{k}$:

#

$$\boldsymbol{\epsilon}_{\boldsymbol{k}}\boldsymbol{=}\left( {{\boldsymbol{\beta}_{\boldsymbol{2}}}_{\boldsymbol{Sample size}}\boldsymbol{+\beta}}_{\boldsymbol{3}_{\boldsymbol{Study type}}}\boldsymbol{+ \beta}_{\boldsymbol{4}_{\boldsymbol{Study design}}}\boldsymbol{+}\boldsymbol{\beta}_{\boldsymbol{5}_{\boldsymbol{Age [S]}}}\boldsymbol{+}\boldsymbol{\beta}_{\boldsymbol{6}_{\boldsymbol{Age [C]}}}\boldsymbol{+}\boldsymbol{\beta}_{\boldsymbol{7}_{\boldsymbol{Age}}}\boldsymbol{+}\boldsymbol{\beta}_{\boldsymbol{8}_{\boldsymbol{Females}}}\boldsymbol{+}\boldsymbol{\beta}_{\boldsymbol{9}_{\boldsymbol{Females [C]}}}\boldsymbol{+ \beta}_{\boldsymbol{10}_{\boldsymbol{Females [S ]}}}{\boldsymbol{+ \beta}_{\boldsymbol{11}_{\boldsymbol{Diabetes}}\boldsymbol{+}\boldsymbol{\beta}_{\boldsymbol{12}_{\boldsymbol{Nephropathy}}}\boldsymbol{+ \beta}_{\boldsymbol{13}_{\boldsymbol{Epidural abscess.}}}}\boldsymbol{+ \beta}}_{\boldsymbol{14}_{\boldsymbol{IVDU}}}\boldsymbol{+ \beta}_{\boldsymbol{15}_{\boldsymbol{Date of publication}}}\boldsymbol{+ \beta}_{\boldsymbol{16}_{\boldsymbol{Cervical}}} \boldsymbol{+ \beta}_{\boldsymbol{17}_{\boldsymbol{Thoracic}}}\boldsymbol{+}\boldsymbol{+ \beta}_{\boldsymbol{18}_{\boldsymbol{Lumbar}}} \right)\boldsymbol{x}_{\boldsymbol{k}}$$

The error term $\epsilon_{k}$is hypothesized to be influenced by the sample size ($\beta_{2_{Sample size}})$, the study type ($\beta_{3_{Study type}})$, the study design ($\beta_{4_{Study design}})$, the mean age of the surgically managed patients ($\beta_{5_{Age [S]}})$, the mean age of the conservatively managed patients ($\beta_{6_{Age [C]}})$the mean overall age of the study sample ($\beta_{7_{Age}})$, the proportion of females in the study sample ($\beta_{8_{Age}}$), the mean proportion of females in the conservatively managed patient group ($\beta_{9_{Females [C]}}$), the proportion of females in the surgically managed patient group ($\beta_{{10}_{Females [S]}}$), the overall proportion of diabetics in the study sample ($\beta_{{11}_{Diabetes}}$), the proportion of patients with nephropathy ($\beta_{{12}_{Nephropathy}}$), the proportion of patients with an epidural abscess ($\beta_{{13}_{Depression}}$), the proportion of patients who are intravenous drugs users ($\beta_{{14}_{IVDU}}$), the date of publication ($\beta_{{15}_{Date of publication.}}$), the proportion of infections being in the cervical spine ($\beta_{{16}_{Cervical}}$), the proportion of infections being in the thoracic spine ($\beta_{{17}_{Thoracic}}$), the proportion of infections being in the lumbar spine ($\beta_{{18}_{Lumbar}}$). The different explanatory variables were calculated singularly as sole covariates in separate meta-regressions, and if significant coefficients were yielded, further regression analyses were performed by adding additional covariates to the sole covariate to assess if significance was retained. Finally, an additional meta-analysis was subsequently performed by removing the studies that caused the significant covariates. The significant studies were identified by examining influence of outliers in analysis.

Supplementary File 2: Correlation analysis findings.

The main significant negative correlations (p<0.001) are: mortality in conservatively treated patients and proportion of IVDU, cervical location of infection and thoracic & lumbar location of infection, cervical location of infection and proportion of nephropathy in surgically managed patients, cervical location and thoracic location of infection. Other notable significant negative correlations are: the proportion of IVDU and mean overall mortality (p<0.01), the proportion of IVDU and mean mortality in surgically treated patients (p<0.01), cervical location of infection and mean mortality in surgically treated patients (p<0.01), cervical location of infection and mean mortality in conservatively treated patients (p<0.01), dropout rate and relapse/failure in surgically managed patients (p<0.05), dropout rate and mean mortality in surgically managed patients (p<0.05),

The main significant positive correlations, ranked by statistical significance (p<0.001), are: mean mortality in surgically treated patients and mean mortality in conservatively treated patients, mean overall relapse/failure rate and relapse/failure in surgically managed patients, relapse/failure in conservatively managed patients and mean overall mortality, the proportion of epidural abscess and the proportion of diabetics, the proportion of diabetics in conservatively treated patients and the proportion of diabetics in surgically treated patients, the proportion of patients with renal disease and the proportion of diabetics in surgically treated patients, thoracic location of infection and proportion of diabetics in surgically treated patients. Other notable positive significant correlations are: the proportion of epidural abscess and relapse/failure in conservatively treated patients (p<0.01), the proportion of diabetics and relapse/failure in surgically treated patients (p<0.01), mean overall relapse/failure rate and proportion of positive cultures (tissues and blood) (p<0.01), relapse/failure in conservatively treated patients and mean mortality in surgically treated patients (p<0.01), relapse/failure in surgically treated patients and mean mortality in surgically treated patients (p<0.01), thoracic location of infection and mean mortality in surgically treated patients (p<0.01), mean mortality in surgically managed patients and proportion of diabetics in surgically managed patients (p<0.01), the proportion of epidural abscess and relapse/failure in surgically treated patients (p<0.05), mean overall relapse/failure rate and mean age of study population (p<0.05), the proportion of positive cultures (tissues and blood) and relapse/failure in conservatively treated patients (p<0.05), the proportion of positive cultures (tissues and blood) and mean overall mortality (p<0.05), relapse/failure in conservatively treated patients and mean age of study population (p<0.05), relapse/failure in surgically treated patients and thoracic location of infection (p<0.05), the proportion of diabetics in conservatively treated patients and mean mortality in surgically managed patients (p<0.05), the proportion of renal disease and mean mortality in surgically managed patients (p<0.05), thoracic location of infection and mean overall mortality (p<0.05), mean overall mortality and proportion of diabetics in surgically treated patients (p<0.05) and mean mortality in surgically managed patients and thoracic & lumbar location of infection (p<0.05).

# References

1. Baujat B, Mahé C, Pignon JP, Hill C. A graphical method for exploring heterogeneity in meta‐analyses: application to a meta‐analysis of 65 trials. Statistics in medicine. 2002 Sep 30;21(18):2641-52.
2. Viechtbauer W, Cheung MW. Outlier and influence diagnostics for meta‐analysis. Research synthesis methods. 2010 Apr;1(2):112-25.
3. Pitaro NL, Tang JE, Arvind V, Cho BH, Geng EA, Amakiri UO, Cho SK, Kim JS. Readmission and Associated Factors in Surgical Versus Non-Surgical Management of Spinal Epidural Abscess: A Nationwide Readmissions Database Analysis. Global Spine Journal. 2021 Dec 5:21925682211039185.
4. Adogwa O, Karikari IO, Carr KR, Krucoff M, Ajay D, Fatemi P, Perez EL, Cheng JS, Bagley CA, Isaacs RE. Spontaneous spinal epidural abscess in patients 50 years of age and older: a 15-year institutional perspective and review of the literature. Journal of Neurosurgery: Spine. 2014 Mar 1;20(3):344-9.
5. Lee JH, Kim J, Kim TH. Clinical outcomes in older patients aged over 75 years who underwent early surgical treatment for pyogenic vertebral osteomyelitis. Journal of clinical medicine. 2021 Nov 22;10(22):5451.
6. Berwick BW, Luo TD, Sun KW, Sharp RA, Birkedal JP, O'Gara TJ. Epidural Abscess in the Lumbar Spine: A Single Institution's Experience With Nonsurgical and Surgical Management. Journal of Surgical Orthopaedic Advances. 2019 Jan 1;28(3):224-31.
7. Giampaolini N, Berdini M, Rotini M, Palmisani R, Specchia N, Martiniani M. Non-specific spondylodiscitis: a new perspective for surgical treatment. European Spine Journal. 2022 Feb;31(2):461-72.
8. Alas H, Fernando H, Baker JF, Brown AE, Bortz C, Naessig S, Pierce KE, Ahmad W, Diebo BG, Passias PG. Comparative outcomes of operative relative to medical management of spondylodiscitis accounting for frailty status at presentation. Journal of Clinical Neuroscience. 2020 May 1;75:134-8.
9. Karikari IO, Powers CJ, Reynolds RM, Mehta AI, Isaacs RE. Management of a spontaneous spinal epidural abscess: A single-center 10-year experience. Neurosurgery. 2009 Nov 1;65(5):919-24.
10. Canouï E, Zarrouk V, Canouï-Poitrine F, Desmoulin U, Leflon V, Allaham W, de Lastours V, Guigui P, Fantin B. Surgery is safe and effective when indicated in the acute phase of hematogenous pyogenic vertebral osteomyelitis. Infectious Diseases. 2019 Apr 3;51(4):268-76.
11. Hasan GA, Raheem HQ, Qutub A, Wais YB, Katran MH, Shetty GM. Management of pyogenic spondylodiscitis following nonspinal surgeries: A tertiary care center experience. International Journal of Spine Surgery. 2021 Jun 1;15(3):591-9.
12. Jin Y, Liu A, Overbey JR, Medikonda R, Feghali J, Krishnan S, Ishida W, Pairojboriboon S, Gokaslan ZL, Wolinsky JP, Theodore N. Risk factors for surgical intervention in patients with primary spinal infection on initial presentation. Journal of Neurosurgery: Spine. 2022 Feb 4;1(aop):1-9.
13. Hohenberger C, Schmidt NO, Doenitz C, Ullrich OW, Schebesch KM. Infectious Spondylodiscitis of the Lumbar Spine: Conservative Antibiotic Therapy vs. Antibiotic Therapy with Surgery, and the Time of Surgery. Neurology India. 2022 Jan 1;70(1):155.
14. Do Kim S, Melikian R, Ju KL, Zurakowski D, Wood KB, Bono CM, Harris MB. Independent predictors of failure of nonoperative management of spinal epidural abscesses. The Spine Journal. 2014 Aug 1;14(8):1673-9.
15. Farber SH, Murphy KR, Suryadevara CM, Babu R, Yang S, Feng L, Xie J, Perfect JR, Lad SP. Comparing outcomes of early, late, and non-surgical management of intraspinal abscess. Journal of Clinical Neuroscience. 2017 Feb 1;36:64-71.
16. Lener S, Wipplinger C, Stocsits A, Hartmann S, Hofer A, Thomé C. Early surgery may lower mortality in patients suffering from severe spinal infection. Acta Neurochirurgica. 2020 Nov;162(11):2887-94.
17. Valancius K, Hansen ES, Høy K, Helmig P, Niedermann B, Bünger C. Failure modes in conservative and surgical management of infectious spondylodiscitis. European Spine Journal. 2013 Aug;22(8):1837-44.
18. Kreutzträger M, Lübstorf T, Ekkernkamp A, Blex C, Schwab JM, Kopp MA, Auhuber T, Wüstner G, Liebscher T. Spinal infection with intraspinal abscess or empyema and acute myelopathy: comparative analysis of diagnostics, therapy, complications and outcome in primary care. European Journal of Trauma and Emergency Surgery. 2022 Jun 3:1-0.
19. Sur A, Tsang K, Brown M, Tzerakis N. Management of adult spontaneous spondylodiscitis and its rising incidence. The Annals of The Royal College of Surgeons of England. 2015 Sep 1;97(6):451-5.
20. Uchida K, Nakajima H, Yayama T, Sato R, Kobayashi S, Chen KB, Mwaka ES, Baba H. Epidural abscess associated with pyogenic spondylodiscitis of the lumbar spine; evaluation of a new MRI staging classification and imaging findings as indicators of surgical management: a retrospective study of 37 patients. Archives of orthopaedic and trauma surgery. 2010 Jan;130(1):111-8.
21. Tsai TT, Yang SC, Niu CC, Lai PL, Lee MH, Chen LH, Chen WJ. Early surgery with antibiotics treatment had better clinical outcomes than antibiotics treatment alone in patients with pyogenic spondylodiscitis: a retrospective cohort study. BMC musculoskeletal disorders. 2017 Dec;18(1):1-7.
22. Jung N, Ernst A, Joost I, Yagdiran A, Peyerl-Hoffmann G, Grau S, Breuninger M, Hellmich M, Kubosch DC, Klingler JH, Seifert H. Vertebral osteomyelitis in patients with Staphylococcus aureus bloodstream infection: Evaluation of risk factors for treatment failure. Journal of Infection. 2021 Sep 1;83(3):314-20.
23. Sterne JA, Hernán MA, Reeves BC, Savović J, Berkman ND, Viswanathan M, Henry D, Altman DG, Ansari MT, Boutron I, Carpenter JR. ROBINS-I: a tool for assessing risk of bias in non-randomised studies of interventions. bmj. 2016 Oct 12;355.
